# Supplementary material for: Characterization, Diversity, and Structure-Activity Relationship Study of Lipoamino Acids from Pantoea sp. and Synthetic Analogues
Source: Int J Mol Sci. 2019 Mar 2;20(5):1083. doi: 10.3390/ijms20051083 (PMC6429151; doi:10.3390/ijms20051083)
Supplement: Supplementary file 1 [file ijms-20-01083-s001.pdf]

SUPPORTING INFORMATION FOR :

Characterization, diversity, and structure-activity relationship study of lipoamino acids from *Pantoea* sp. and synthetic analogues

Seindé Touré [1], Sandy Desrat [1], Léonie Pellissier [2], Pierre-Marie Allard [2], Jean-Luc Wolfender [2], Isabelle Dusfour [3], Didier Stien [4], Véronique Eparvier[1,*]

[1] CNRS, Institut de Chimie des Substances Naturelles, UPR 2301, Université Paris-Saclay, 1 avenue de la Terrasse, 91198 Gif-sur-Yvette, France; seindet@gmail.com, sandy.desrat@cnrs.fr

[2] School of Pharmaceutical Sciences, EPGL, University of Geneva, University of Lausanne, Rue Michel Servet 1, CH-1211 Geneva 4, Switzerland; leonie.pellissier@unige.ch, pierre-marie.allard@unige.ch, jean-luc.wolfender@unige.ch

[3] Institut Pasteur de la Guyane (IPG), Unité de Contrôle et Adaptation des Vecteurs, 97306 Cayenne, Guyane française; isabelle.dusfour@pasteur.fr

[4] Sorbonne Université, CNRS, Laboratoire de Biodiversité et Biotechnologie Microbienne, LBBM, Observatoire Océanologique, 66650 Banyuls-sur-mer, France ; didier.stien@cnrs.fr

* Correspondence: veronique.eparvier@cnrs.fr; Tel.: +33-(0)16-982-3679

Table des matières

Table S1. Full NMR spectroscopic data for compound 1 in CD_3OD 4
Figure S1. ^1H NMR spectrum of 1 recorded at 500 MHz in CD_3OD. 5
Figure S2. ^{13}C NMR spectrum of 1 recorded at 125 MHz in CD_3OD. 5
Figure S3. COSY NMR spectrum of 1 recorded at 500 MHz in CD_3OD. 6
Figure S4. HSQC NMR spectrum of 1 recorded at 500 MHz in CD_3OD. 6
Figure S5. HMBC NMR spectrum of 1 recorded at 500 MHz in CD_3OD. 7
Figure S6. HRMS of compound 1 in MeOH. 7
Table S2. Full NMR spectroscopic data for compound 2 (VECD14BF5-17) in CD_3OD 9
Figure S7. ^1H NMR spectrum of 2 recorded at 500 MHz in CD_3OD. 9
Figure S8. 13C NMR spectrum of 2 recorded at 125 MHz in CD_3OD. 9
Figure S9. COSY NMR spectrum of 2 recorded at 500 MHz in CD_3OD. 10
Figure S10. HSQC NMR spectrum of 2 recorded at 500 MHz in CD_3OD 10
Figure S11. HMBC NMR spectrum of 2 recorded at 500 MHz in CD_3OD. 11
Figure S12. HRMS of compound 2 in MeOH. 11
Table S3. Full NMR spectroscopic data for compound 3 in CD_3OD 12
Figure S13. ^1H NMR spectrum of 3 recorded at 500 MHz in CD3OD. 13
Figure S14. ^{13}C NMR spectrum of 3 recorded at 125 MHz in CD3OD 13
Figure S15. COSY NMR spectrum of 3 recorded at 500MHz in CD3OD. 14
Figure S16. HSQC spectrum of 3 recorded at 500MHz in CD3OD 14
Figure S17. HMBC spectrum of 3 recorded at 500MHz in CD3OD. 15
Figure S18. HRMS of compound 3 recorded in MeOH. 15
Figure S19. ^1H spectrum of 4 recorded at 500 MHz in CD3OD. 16
Figure S20. ^{13}C spectrum of 4 recorded at 125 MHz in CD_3OD. 16
Figure S21. HRMS of compound 4 recorded in MeOH. 17
Figure S24. HRMS of compound 3-OMe recorded in MeOH. 17
Figure S25. ^1H spectrum of 5 recorded at 500 MHz in CD_3OD. 18
Figure S26. ^{13}C NMR spectrum for compound 5 in CD_3OD 18
Figure S27. HRMS of compound 5 recorded in MeOH. 19
Figure S28. 1H NMR spectrum for compound 6 in CD_3OD 19
Figure S30. HRMS of compound 6 in MeOH 20
Figure S31. 1H NMR spectrum for compound 7 in CD_3OD 21
Figure S32. ^{13}C NMR spectrum for compound 7 in CD_3OD 21
Figure S33. HRMS of compound 7 in MeOH 22
Figure S34. 1H NMR spectrum for compound 8 in CD_3OD 22
Figure S35. ^{13}C NMR spectrum for compound 8 in CD_3OD 23
Figure S36. HRMS of compound 8 in MeOH 23
Figure S37. 1H NMR spectrum for compound 9 in CD_3OD 24
Figure S38. ^{13}C NMR spectrum for compound 9 in CD_3OD 24
Figure S39. HRMS of compound 9 in MeOH 25
Figure S40. 1H NMR spectrum for compound 10 in CD_3OD 25
Figure S41. ^{13}C NMR spectrum for compound 10 in CD_3OD 26
Figure S42. HRMS of compound 10 in MeOH 26
Figure S43. 1H NMR spectrum for compound 11 in CD_3OD 27
Figure S44. ^{13}C NMR spectrum for compound 11 in CD_3OD 27
Figure S45. HRMS of compound 11 in MeOH 28
Figure S46. 1H NMR spectrum for compound *ent*-3OMe in CD_3OD 28
Figure S47. ^{13}C NMR spectrum for compound *ent*-3OMe in CD_3OD 29
Figure S48. HRMS of compound *ent*-3OMe in MeOH 29
Figure S49. ^1H NMR spectrum for compound 12 in CD_3OD 30
Figure S50. ^{13}C NMR spectrum for compound 12 in CD_3OD 30
Figure S51. HRMS of compound 12 in MeOH 31
Figure S52. 1H NMR spectrum for compound 13 in CD_3OD 31
Figure S53. ^{13}C NMR spectrum for compound 13 in CD_3OD 32
Figure S54. HRMS of compound 13 in MeOH 32
Figure S55. 1H NMR spectrum for compound 14 in CD_3OD 33
Figure S57. HRMS of compound 14 in MeOH 34
Figure S58. 1H NMR spectrum for compound 15 in CD_3OD 34
Figure S59. ^{13}C NMR spectrum for compound 15 in CD_3OD 35
Figure S60. HRMS of compound 15 in MeOH 35
Figure S61. 1H NMR spectrum for compound 16 in CD_3OD 36
Figure S62. ^{13}C NMR spectrum for compound 16 in CD_3OD 36
Figure S63. HRMS of compound 16 in MeOH 37
Figure S64. 1H NMR spectrum for compound 17 in CD_3OD 37
Figure S65. ^{13}C NMR spectrum for compound 17 in CD_3OD 38
Figure S66. HRMS of compound 17 in MeOH 38
Figure S67. 1H NMR spectrum for compound 18 in CD_3OD 39
Figure S69. HRMS of compound 18 in MeOH 40
Figure S72. HRMS of compound 19 in MeOH 41
Figure S75. HRMS of compound 20 in MeOH 43
Figure S76. 1H NMR spectrum for compound 3 in CD_3OD 43
Figure S78. HRMS of compound 3 in MeOH 44
Figure S80. ^{13}C NMR spectrum for compound 21 in CD_3OD 45

Figure S81. HRMS of compound 21 in MeOH **46**
Figure S82. 1H NMR spectrum for compound 22 in CD_3OD **46**
Figure S83. ^{13}C NMR spectrum for compound 22 in CD_3OD **47**
Figure S84. HRMS of compound 22 in MeOH **47**
Figure S85. 1H NMR spectrum for compound 23 in CD_3OD **48**
Figure S86. ^{13}C NMR spectrum for compound 23 in CD_3OD **48**
Figure S87. HRMS of compound 23 in MeOH **49**
Figure S88. 1H NMR spectrum for compound 24 in CD_3OD **49**
Figure S89. ^{13}C NMR spectrum for compound 24 in CD_3OD **50**
Figure S90. HRMS of compound 24 in MeOH **50**
Figure S93. HRMS of compound 25 in MeOH **52**
Figure S94. 1H NMR spectrum for compound 26 in CD_3OD **52**
Figure S95. ^{13}C NMR spectrum for compound 26 in CD_3OD **53**
Figure S96. HRMS of compound 26 in MeOH **53**
Figure S99. HRMS of compound 27 in MeOH **55**
Figure S102. HRMS of compound *ent*-3 in MeOH **56**
Figure S104. ^{13}C NMR spectrum for compound 28 in CD_3OD **57**
Figure S105. HRMS of compound 28 in MeOH **58**
Figure S106. 1H NMR spectrum for compound 29 in CD_3OD **58**
Figure S107. ^{13}C NMR spectrum for compound 29 in CD_3OD **59**
Figure S108. HRMS of compound 29 in MeOH **59**
Figure S111. HRMS of compound 30 in MeOH **61**
Figure S114. HRMS of compound 31 in MeOH **62**
Figure S117. HRMS of compound 32 in MeOH **64**
Figure S118. 1H NMR spectrum for compound 33 in CD_3OD **64**
Figure S120. HRMS of compound 33 in MeOH **65**
Figure S121. ^{1}H NMR spectrum for compound 34 in CD_3OD **66**
Figure S122. ^{13}C NMR spectrum for compound 34 in CD_3OD **66**
Figure S123. HRMS of compound 34 in MeOH **67**
Figure S124. 1H NMR spectrum for compound 35 in CD_3OD **67**
Figure S125. ^{13}C NMR spectrum for compound 35 in CD_3OD **68**
Figure S126. HRMS of compound 35 in MeOH **68**
Table S4. Annotation of possible adduct or complexes **69**
Table S5. Optical rotation of synthetic compounds **77**

Table S1. Full NMR spectroscopic data for compound 1 in CD_3OD

Position	δ_C type	δ_H m (*J* in Hz)	COSY	HMBC
1	176.02. C			
2	55.9. CH	4.6. m	H3a. H3b	C3. C4
3	38.7. CH_2	2.98. dd (13.9. 8.2)	H2. H3b	C2. C4. C5
		3.21. dd (13.9. 4.5)	H2. H3a	C1. C2. C4. C5
4	139.0. C			
5	130.5. CH	7.24. m		C3. C7
6	129.3. CH	7.25. m		C4. C7
7	127.6. CH	7.18. m		C5
1′	173.8. C			
2′	44.6. CH_2	2.29. dd (14.4. 5.2)		C1′. C3′. C4′
		2.34. dd (14.4. 7.4)		
3′	69.6. CH	3.85. m	H2′a. H4′	
4′	38.0. CH_2	1.40. m	H5′	
5′	30.9. CH_2	1.29. br s	H4′	
6′	30.9. CH_2	1.29. br s		
7′	30.9. CH_2	1.29. br s		
8′	30.9. CH_2	1.29. br s		
9′	30.9. CH_2	1.29. br s		
10′	30.9. CH_2	1.29. br s		
11′	30.9. CH_2	1.29. br s		
12′	30.9. CH_2	1.29. br s		
13′	23.6. CH_2	1.31. m	14′	
14′	14.4. CH_3	0.90. t (6.8)	13′	13′

Figure S1. ^{1}H **NMR spectrum of 1 recorded at 500 MHz in** CD_3OD**.**

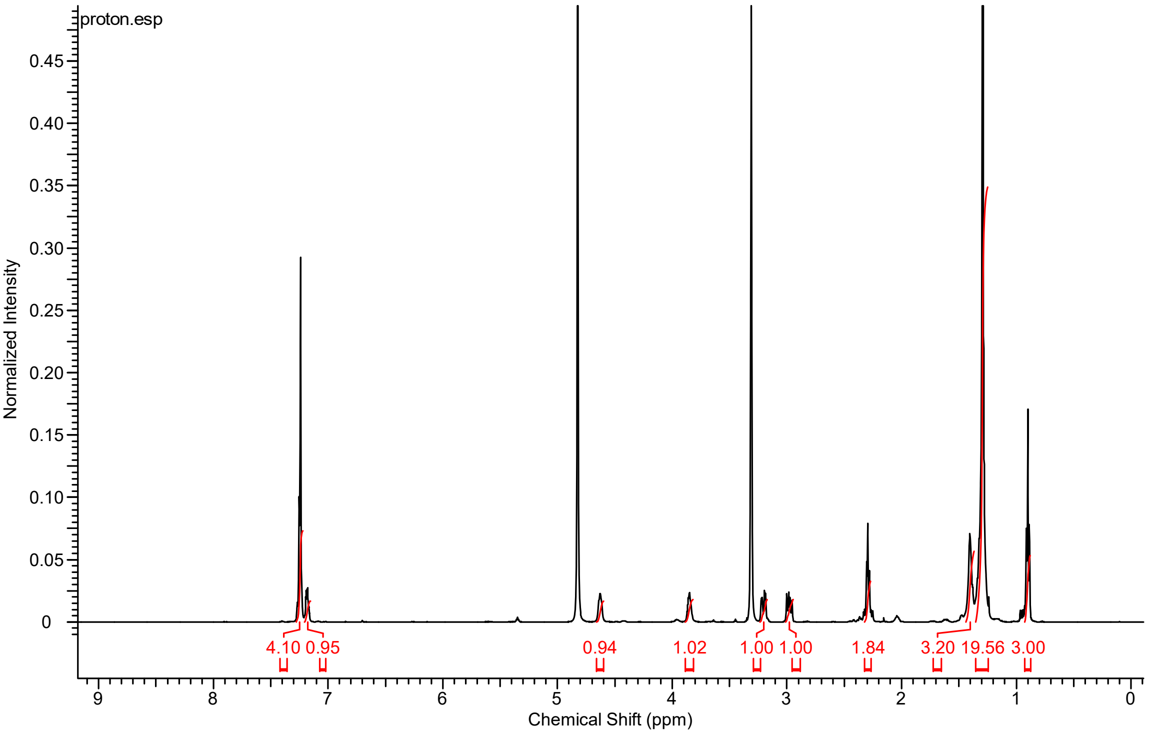

Figure S2. ^{13}C **NMR spectrum of 1 recorded at 125 MHz in** CD_3OD**.**

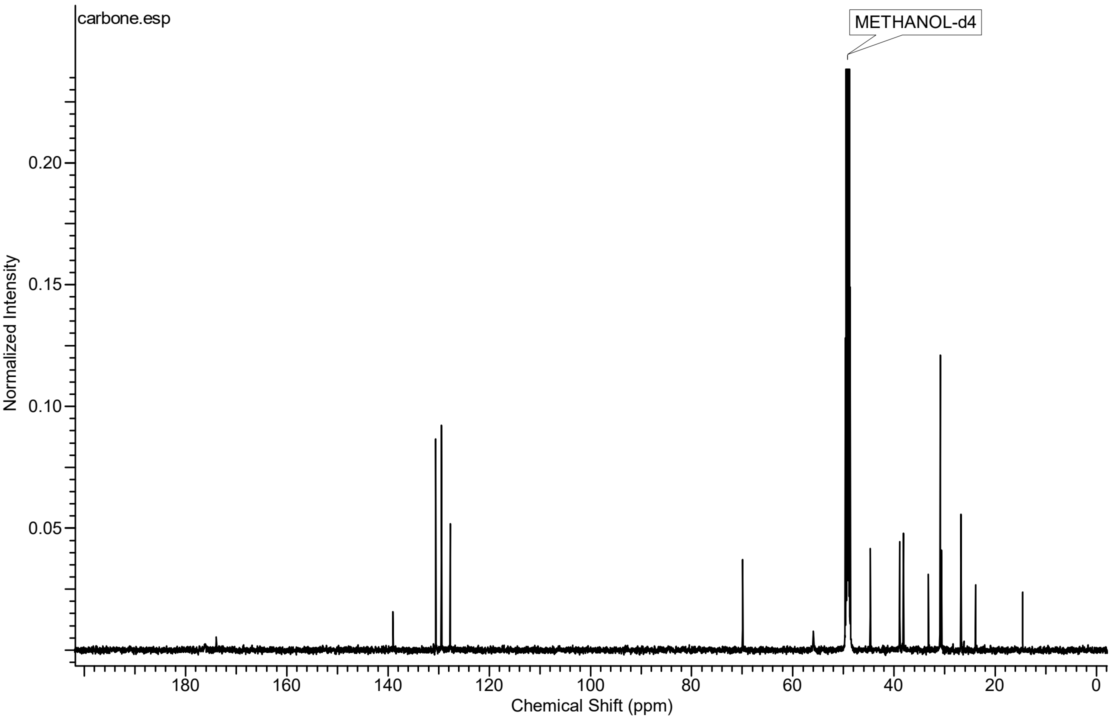

Figure S3. COSY NMR spectrum of 1 recorded at 500 MHz in CD_3OD.

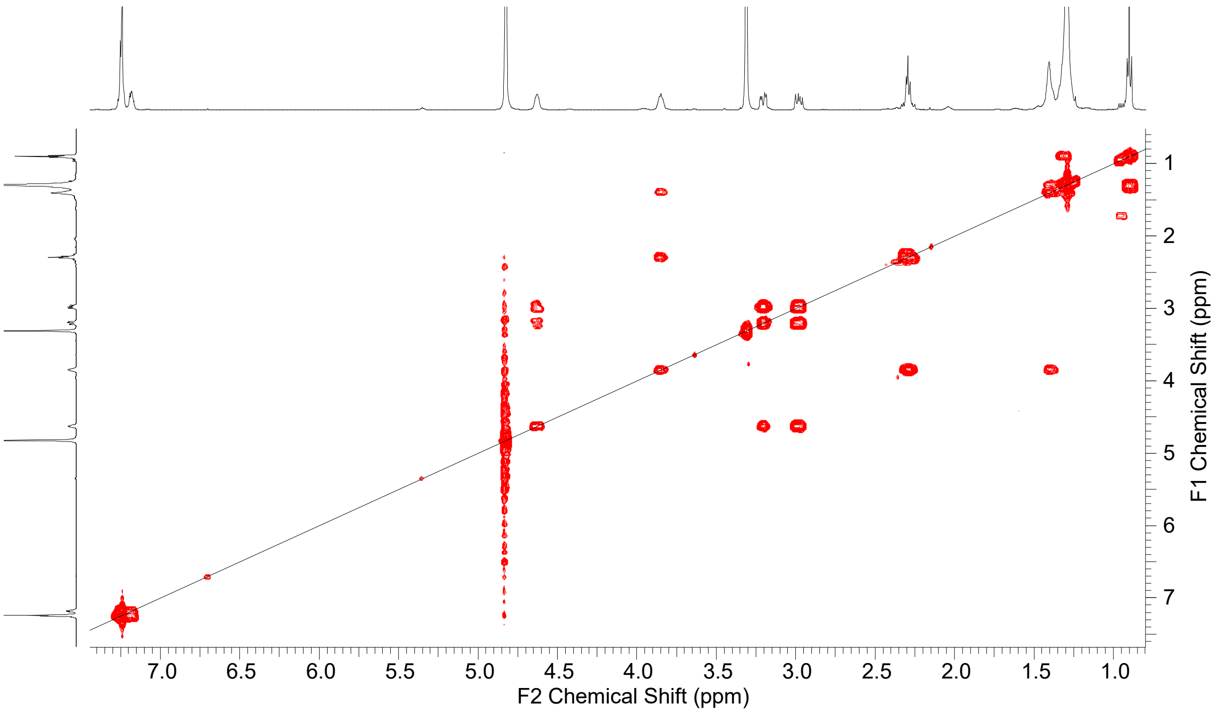

Figure S4. HSQC NMR spectrum of 1 recorded at 500 MHz in CD_3OD.

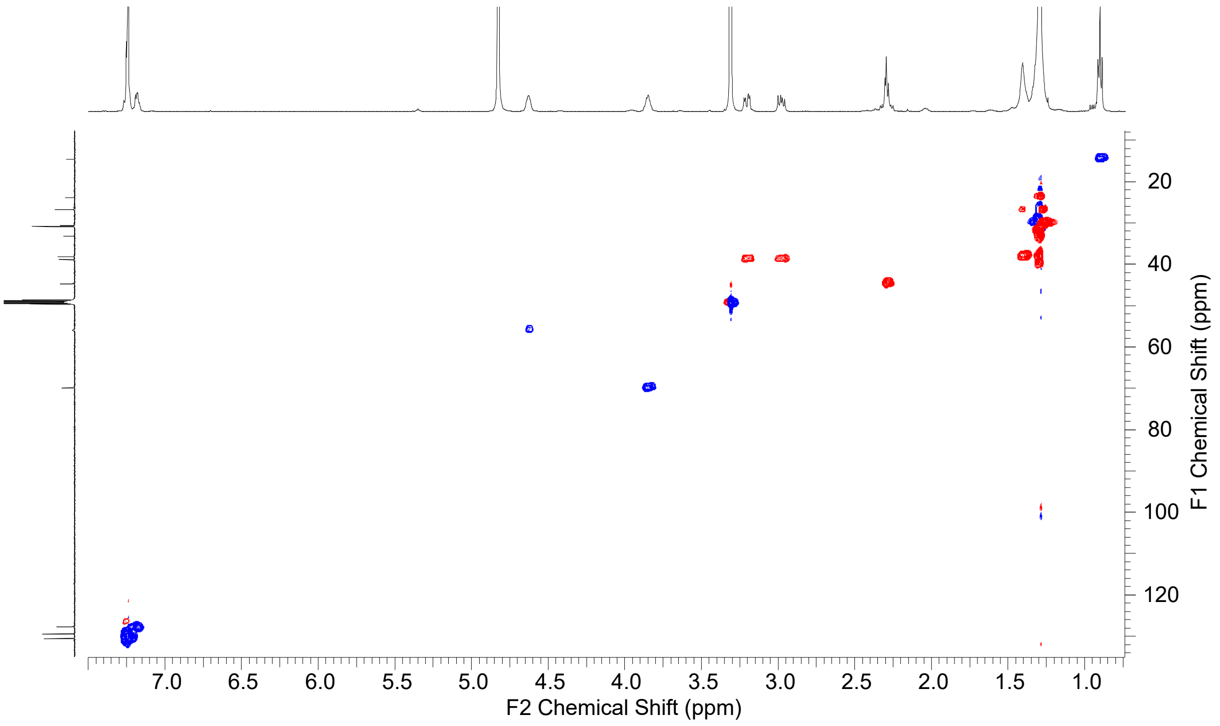

Figure S5. HMBC NMR spectrum of 1 recorded at 500 MHz in CD_3OD.

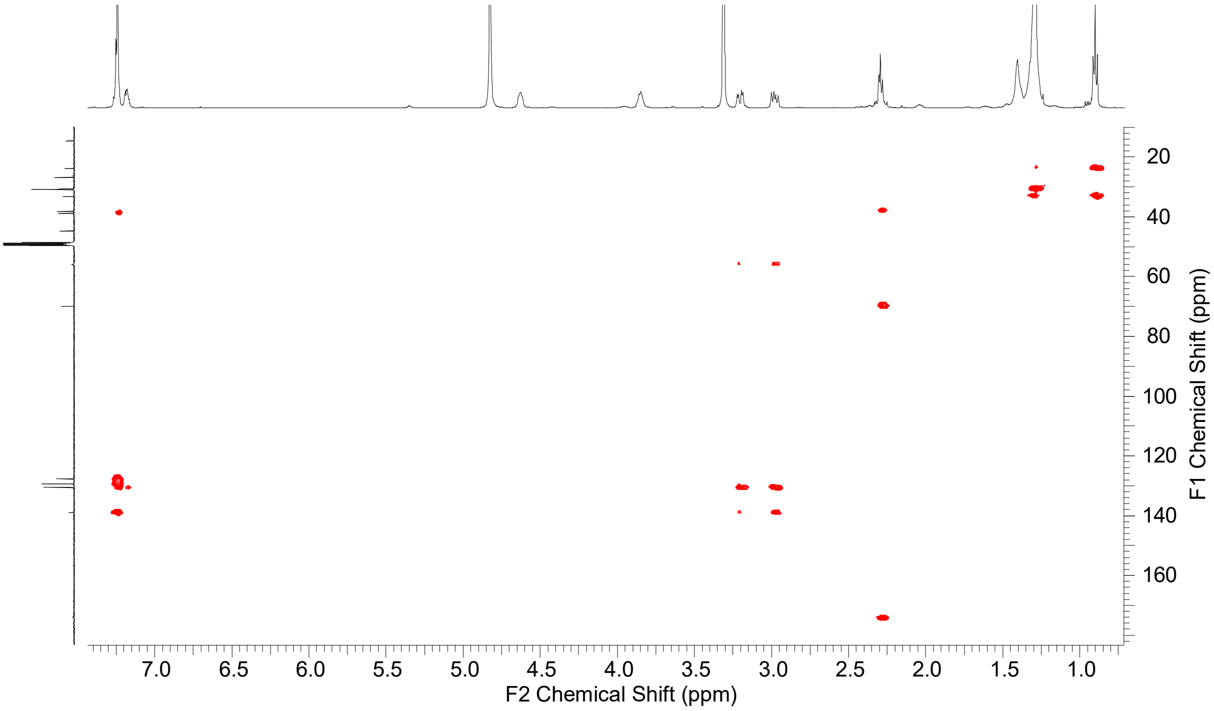

Figure S6. HRMS of compound 1 in MeOH.

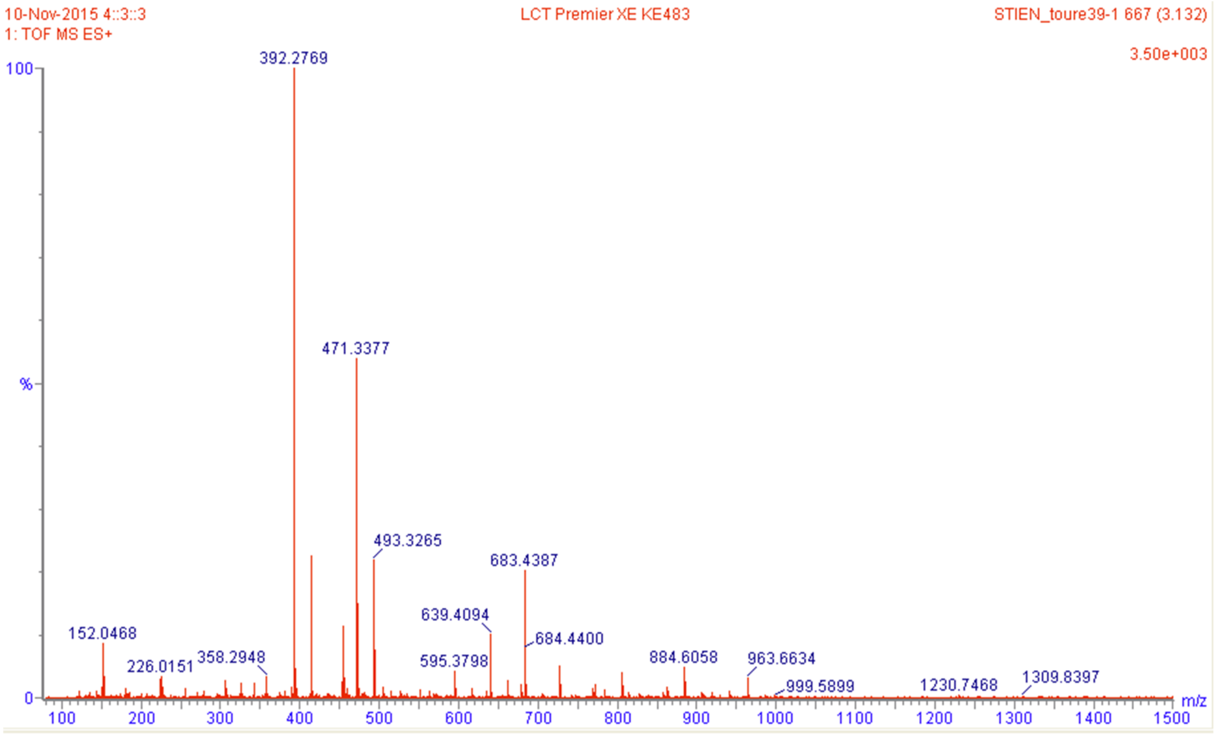

Table S2. Full NMR spectroscopic data for compound 2 (VECD14BF5-17) in CD_3OD

Position	δ_C type	δ_H m (*J* in Hz)	COSY	HMBC
1	176.3. C			
2	57.6. CH	4.53. m	H3	
3	39.4. CH_2	2.97. dd (14.0. 7.7)	H2	C2. C4. C5
		3.21. dd (14.0. 4.5)		
4	139.8. C			
5	130.7. CH	7.24. m	H6. H7	C3. C7
6	129.3. CH	7.22. m	H5. H7	C4. C7
7	127.3. CH	7.15. m	H5. H6	C5
1’	173.5. C			
2’	44.9. CH_2	2.25. dd (14.4. 7.8)	H3’	C1’. C3’. C4’
		2.30. dd (14.5. 4.9)		C1’. C3’. C4’
3’	67.9. CH	3.85. m	H2’. H4’	C1’
4’	38.2. CH_2	1.39. m	H3’	
5’	31.0. CH_2	1.30. m		C3’
6’	31.0. CH_2	1.30. m		
7’	31.0. CH_2	1.30. m		
8’	28.3. CH_2	2.04. m	H9’	C7’. C9’
9’	130.9. CH	5.4. m	H8’	C8’
10’	130.9. CH	5.4. m	H11’	C11’
11’	28.3. CH_2	2.04. m	H10’	C10’. C12’
12’	31.0. CH_2	1.30. m		
13’	31.0. CH_2	1.30. m		
14’	31.0. CH_2	1.30. m		
15’	23.9. CH_2	1.30. m	H16’	
16’	14.6. CH_3	0.90. t (6.8)	H15’	C14’. C15’

Figure S7. ^{1}H NMR spectrum of 2 recorded at 500 MHz in CD_3OD.

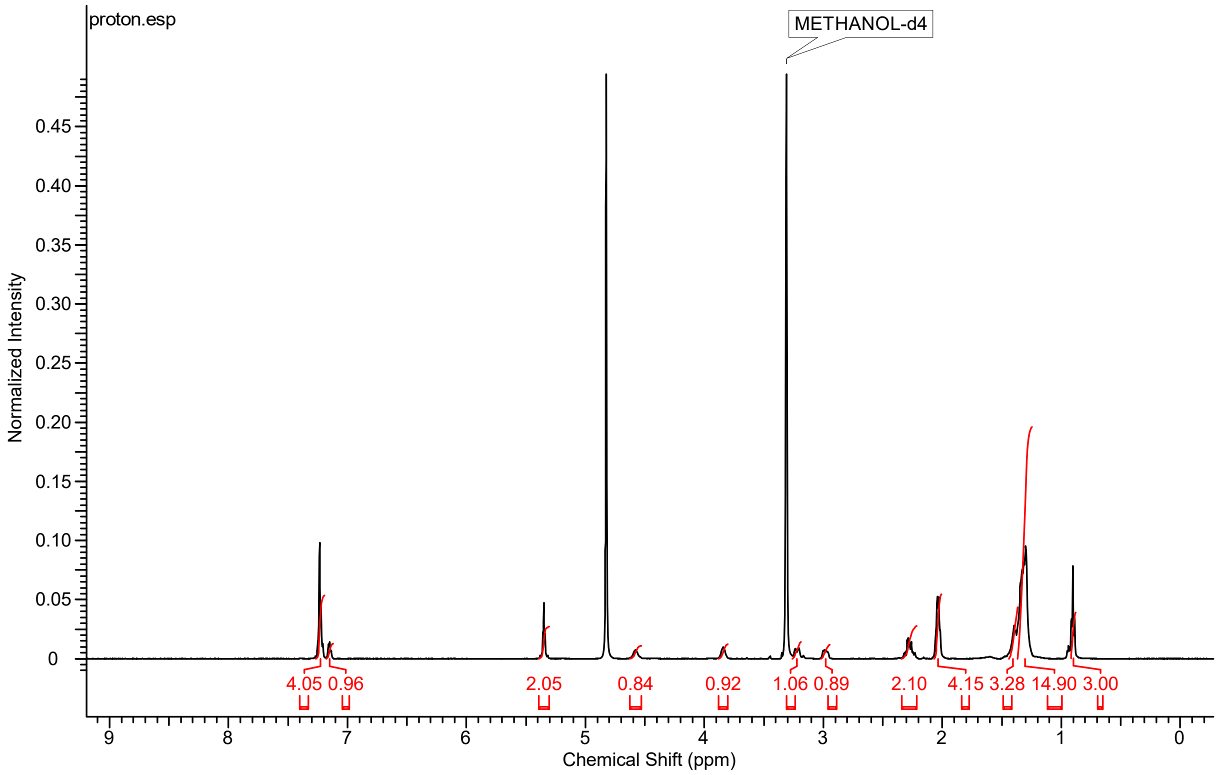

Figure S8. 13C NMR spectrum of 2 recorded at 125 MHz in CD_3OD.

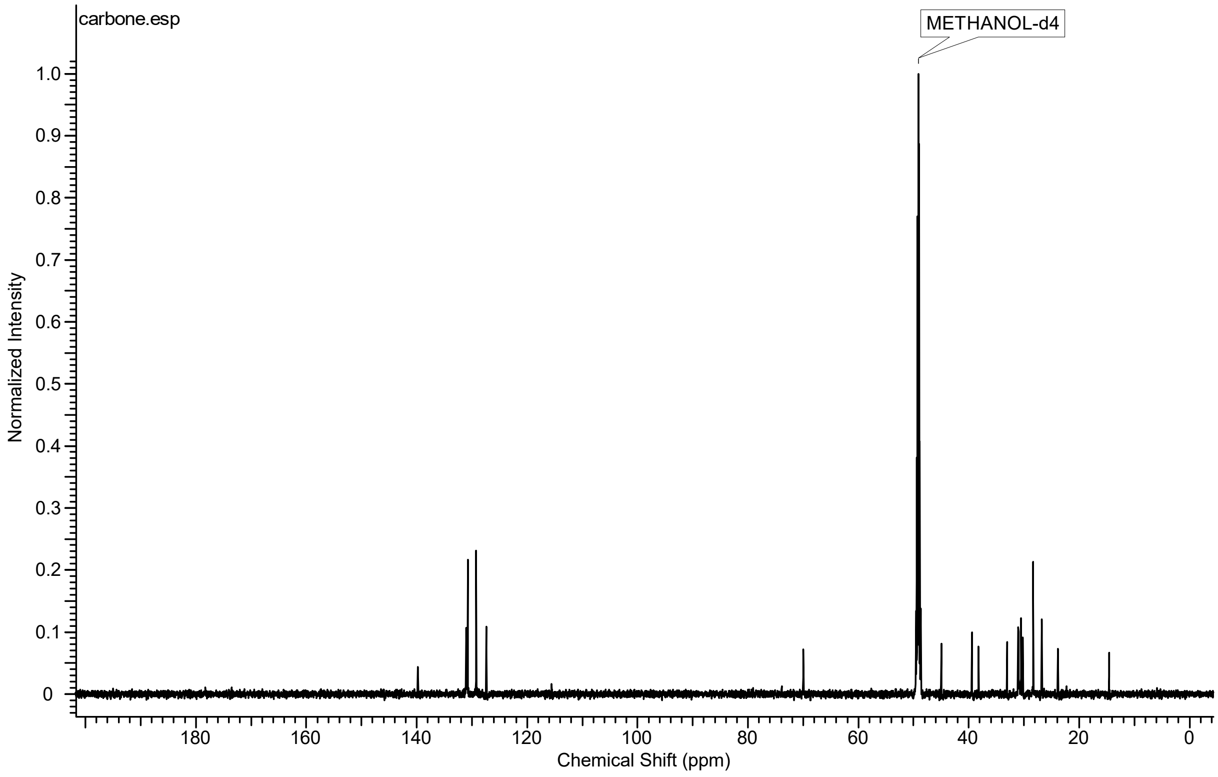

Figure S9. COSY NMR spectrum of 2 recorded at 500 MHz in CD_3OD.

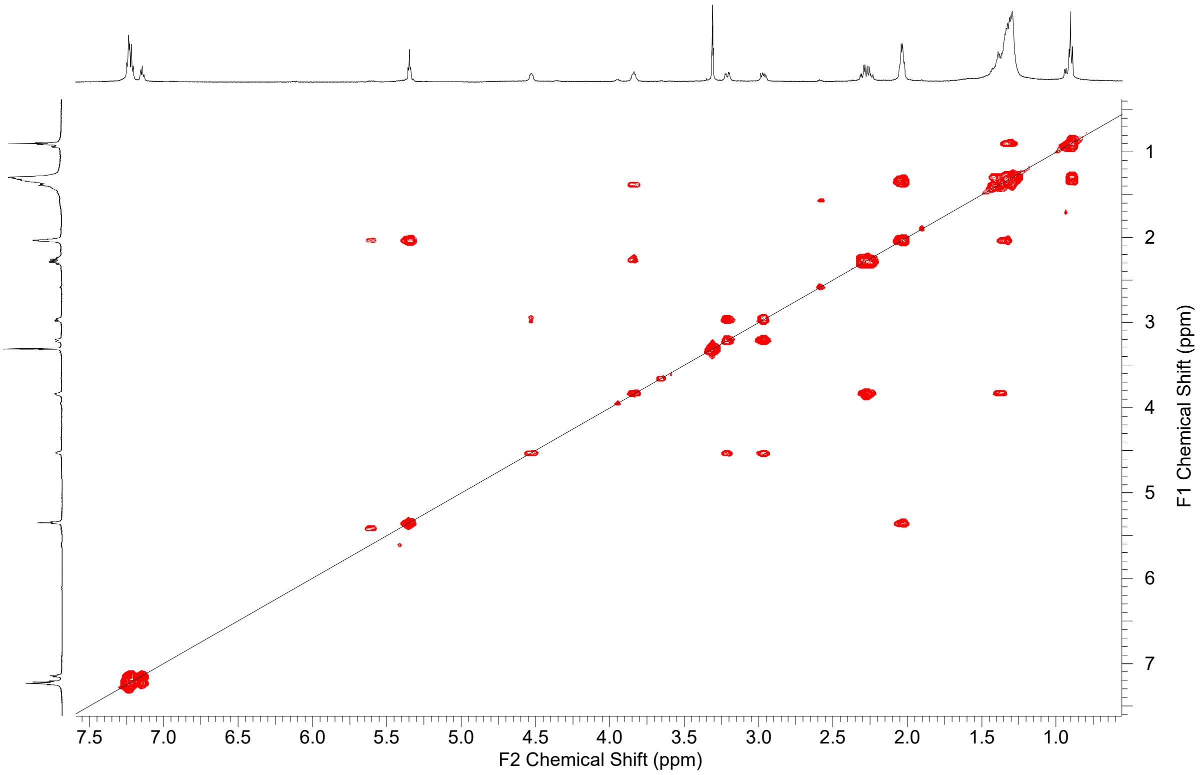

Figure S10. HSQC NMR spectrum of 2 recorded at 500 MHz in CD_3OD.

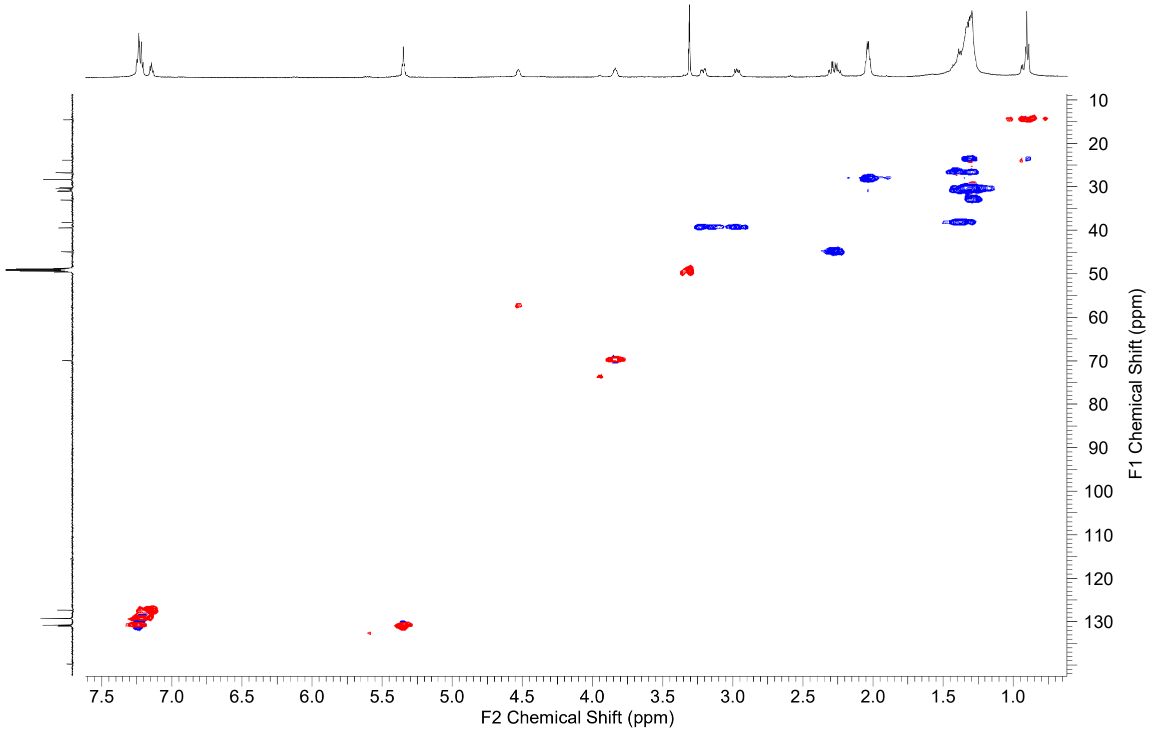

Figure S11. HMBC NMR spectrum of 2 recorded at 500 MHz in CD_3OD.

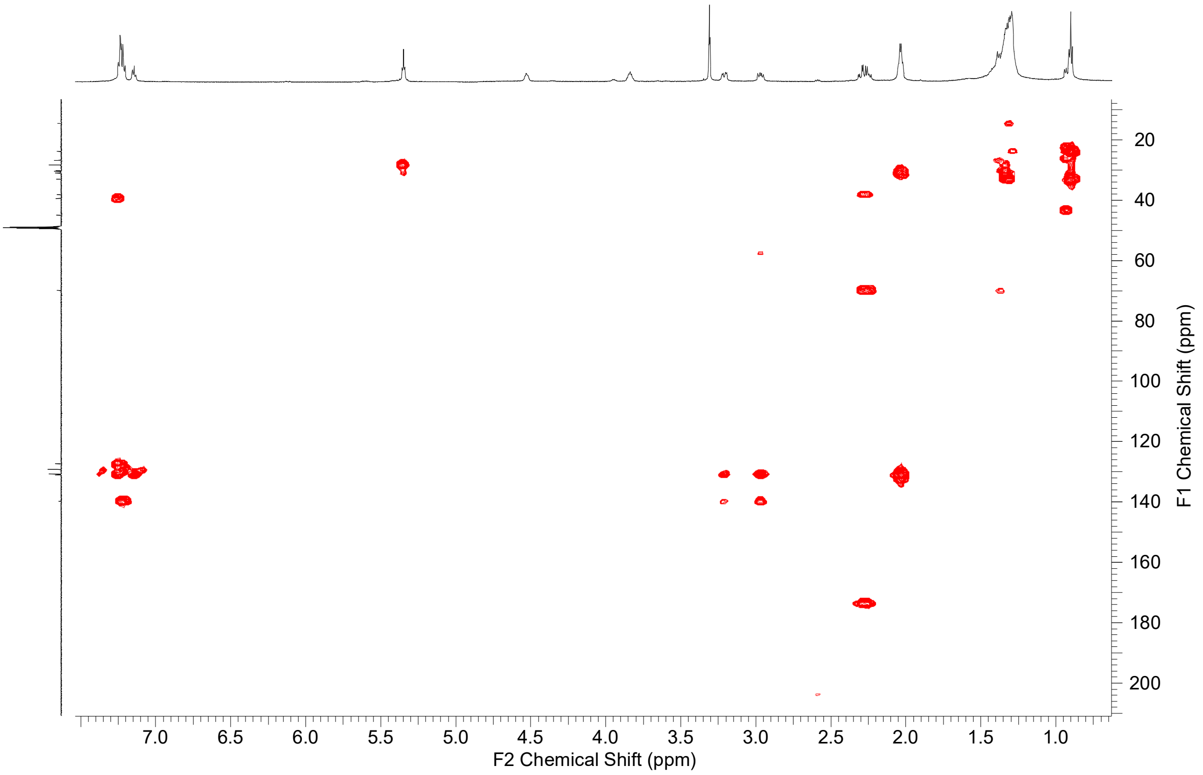

Figure S12. HRMS of compound 2 in MeOH.

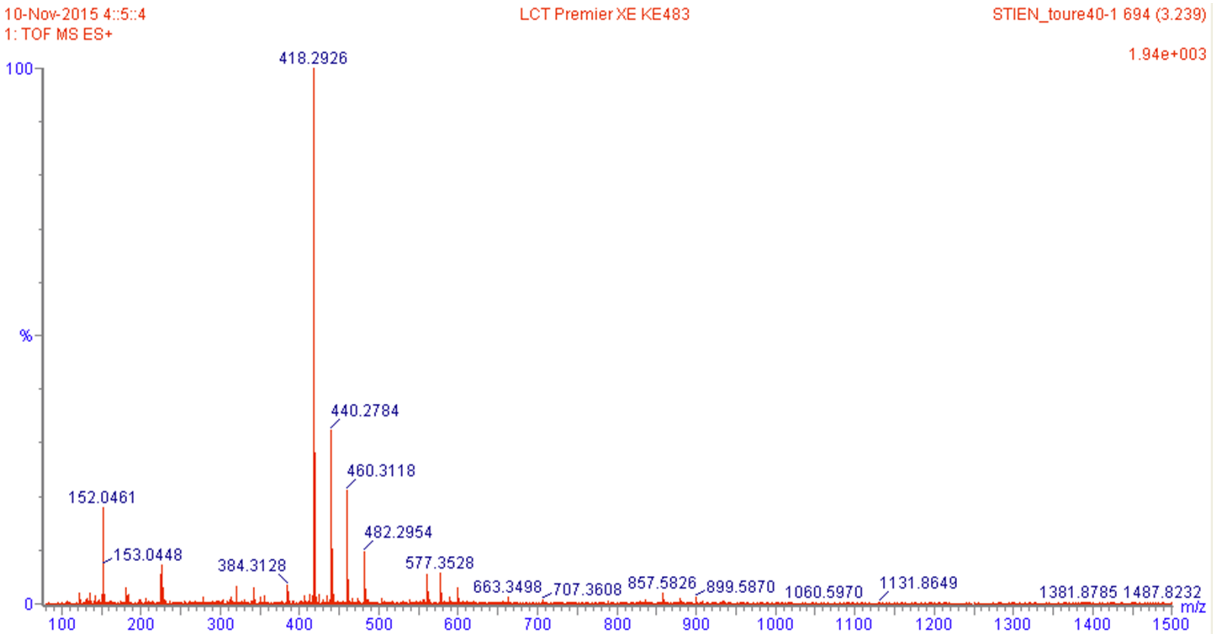

Table S3. Full NMR spectroscopic data for compound 3 in CD_3OD

Position	δ_C type	δ_H m (*J* in Hz)	COSY	HMBC
1	176.5. C			
2	56.02. CH	4.62. dd (8.0. 4.7)	H3a. H3b	C1. C3. C4. C1′
3	39.0. CH_2	2.94. dd (13.8. 8.7)	H2	C1. C2. C4. C5
		3.22. dd (13.8. 4.7)	H2	C1. C2. C4. C5
4	139.2. C			
5	130.5. CH	7.23. m		C3. C4. C7
6	129.4. CH	7.24. m		C4. C7
7	127.6. CH	7.17. m		C5
1′	175.8. C			
2′	37.2. CH_2	2.13. t (7.5)	H3′	C1′. C3′. C4′
		2.23. t (7.5)		C1′. C3′. C4′
3′	27.1. CH_2	1.48. m	H2′a. H4′	C1′. C2′. C4′
4′	30.3. CH_2	1.29. br s	H3′	
5′	30.3. CH_2	1.29. br s		
6′	30.3. CH_2	1.29. br s		
7′	30.3. CH_2	1.29. br s		
8′	30.3. CH_2	2.03. m	H9′	C7′. C9′
9′	131.0. CH	5.35. m	H8′	C7′. C8′
10′	131.0. CH	5.35. m	H11′	C11′. C12′
11′	30.3. CH_2	2.03. m	H10′. H12′	C10′
12′	30.5. CH_2	1.33. br s	H11′	
13′	30.5. CH_2	1.33. br s		
14′	30.5. CH_2	1.33. br s		
15′	23.8. CH_2	1.31. br s	H16′	
16′	14.6. CH_3	0.90. t (6.8)	H15′	C15′. C14′

Figure S13. ^{1}**H NMR spectrum of 3 recorded at 500 MHz in CD**$_3$**OD.**

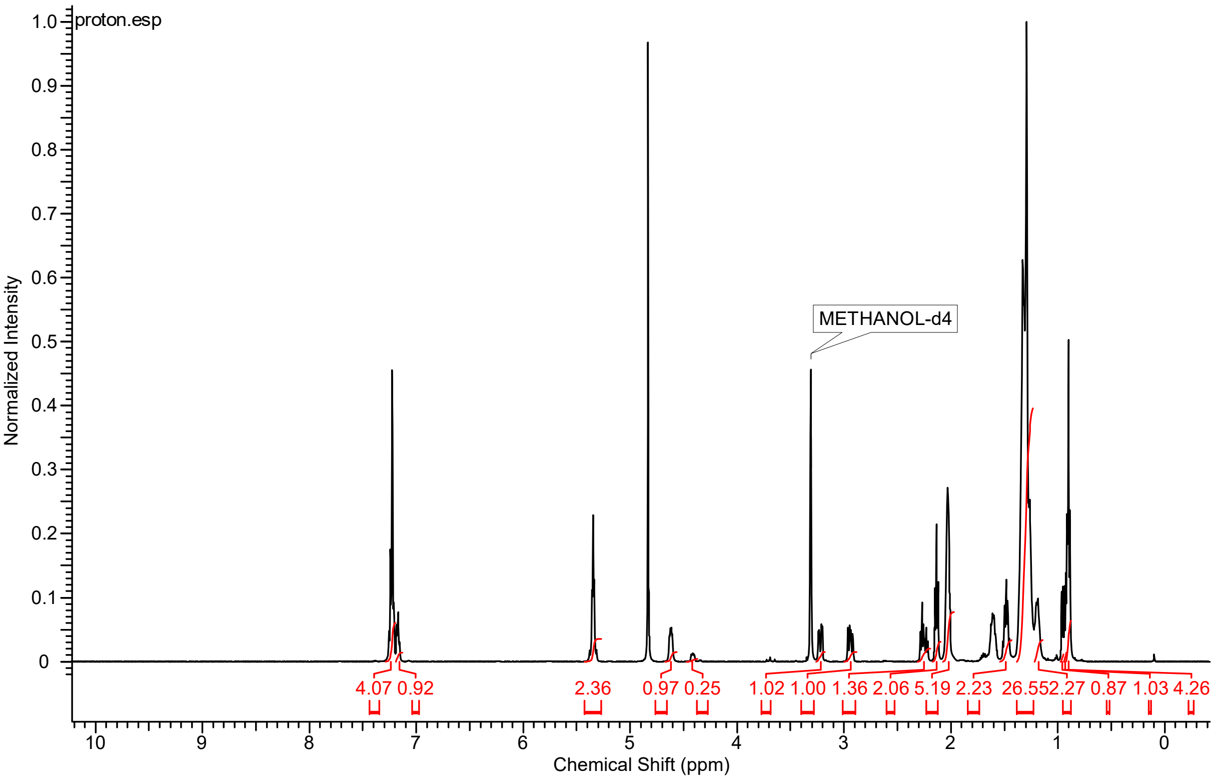

Figure S14. ^{13}**C NMR spectrum of 3 recorded at 125 MHz in CD**$_3$**OD.**

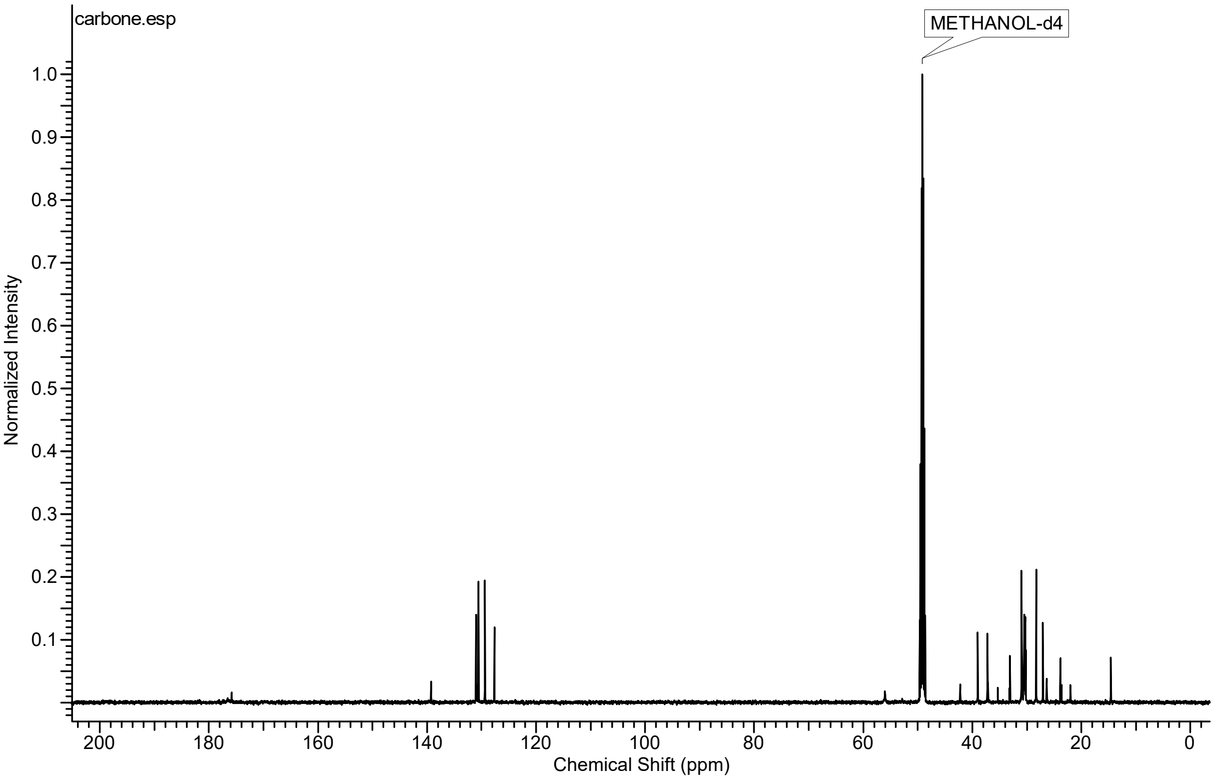

Figure S15. COSY NMR spectrum of 3 recorded at 500MHz in CD3OD.

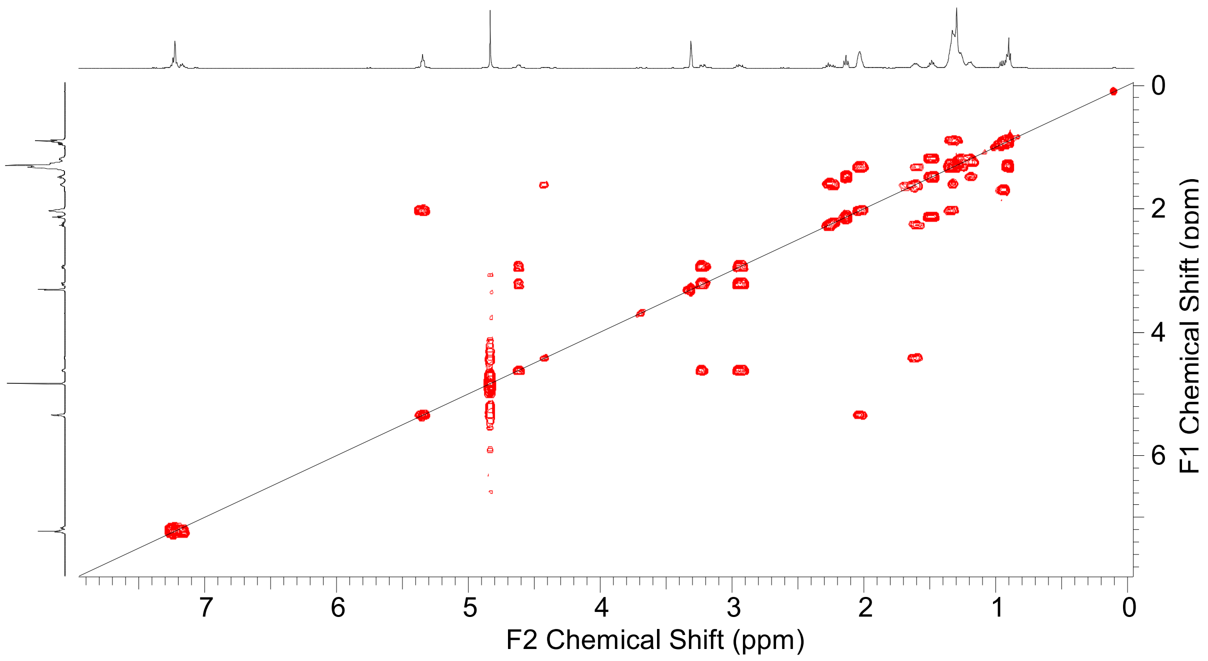

Figure S16. HSQC spectrum of 3 recorded at 500MHz in CD3OD.

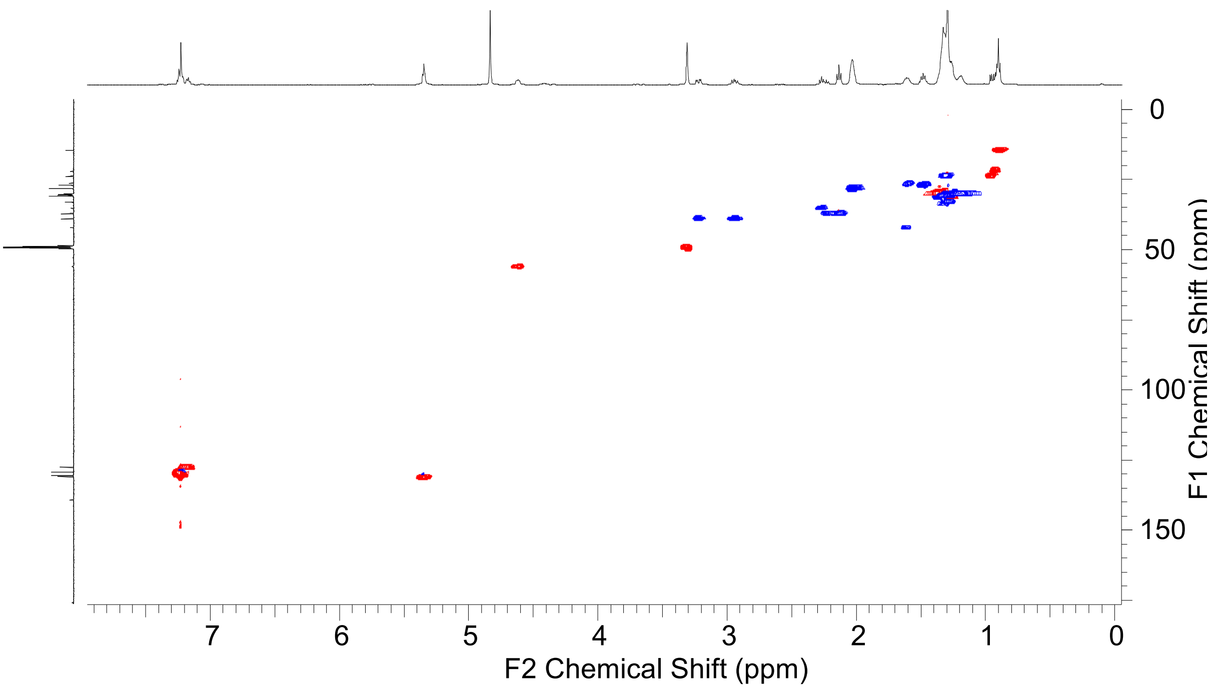

Figure S17. HMBC spectrum of 3 recorded at 500MHz in CD_3OD.

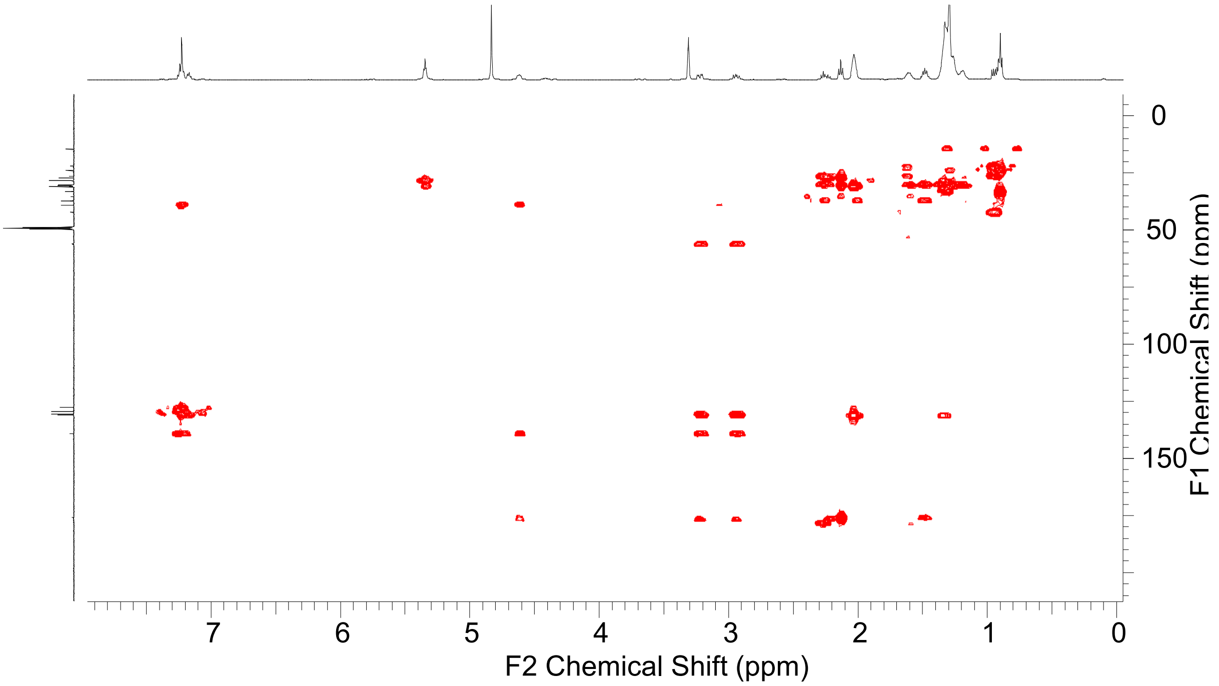

Figure S18. HRMS of compound 3 recorded in MeOH.

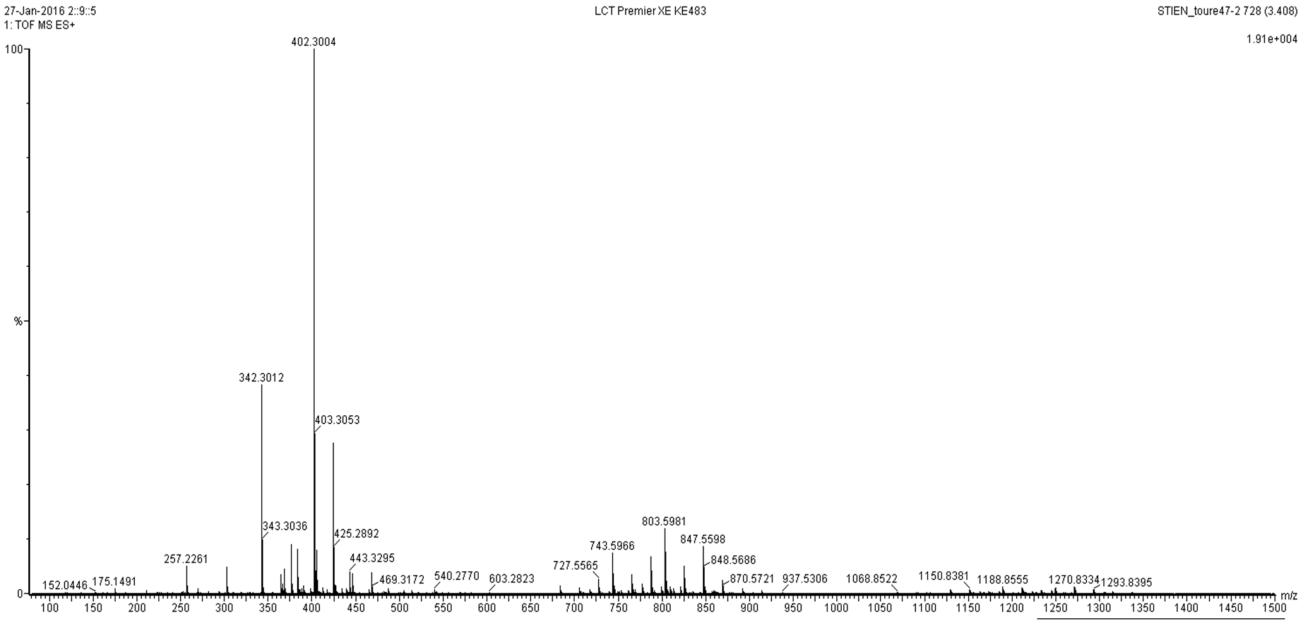

Figure S19. ^{1}H spectrum of 4 recorded at 500 MHz in CD_3OD.

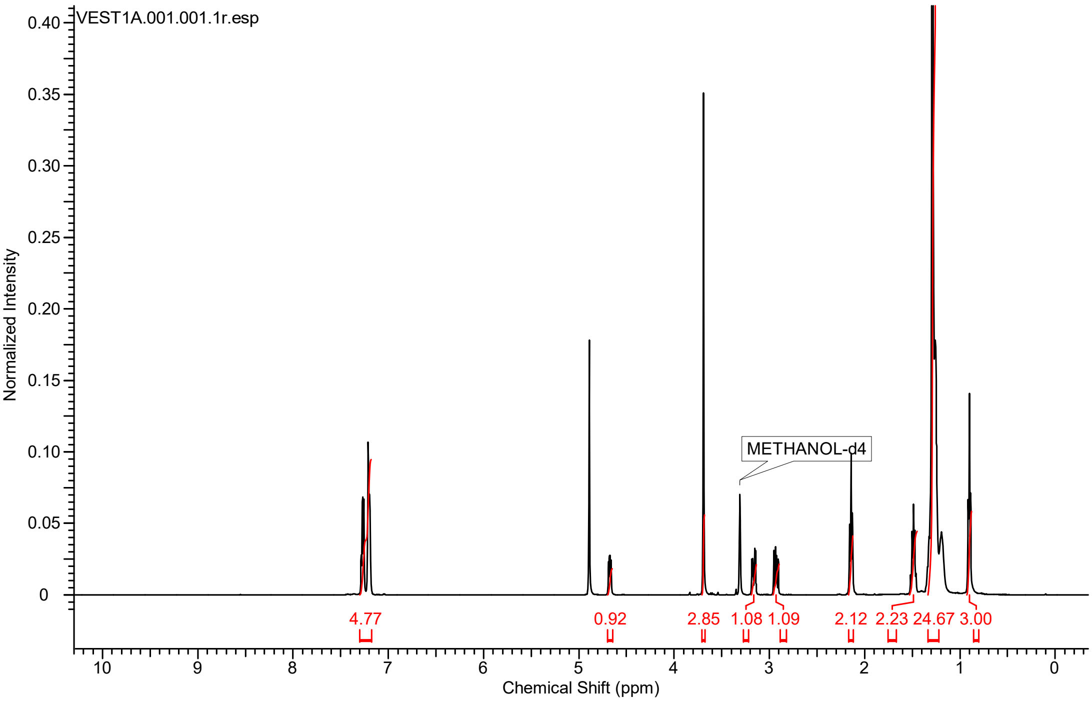

Figure S20. ^{13}C spectrum of 4 recorded at 125 MHz in CD_3OD.

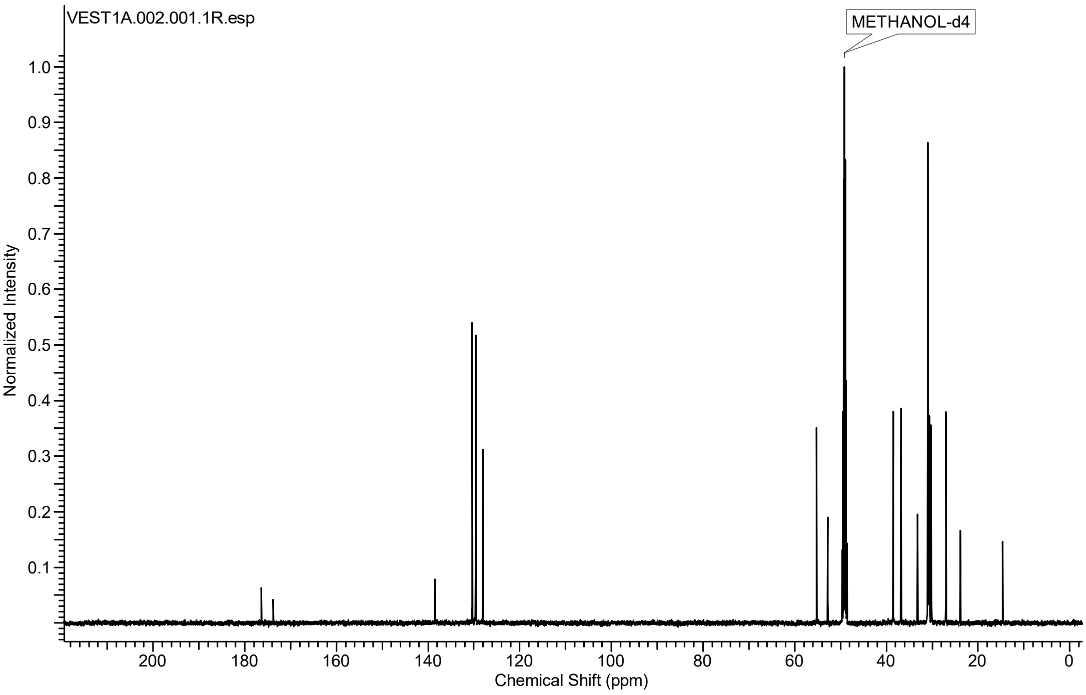

Figure S21. HRMS of compound 4 recorded in MeOH.

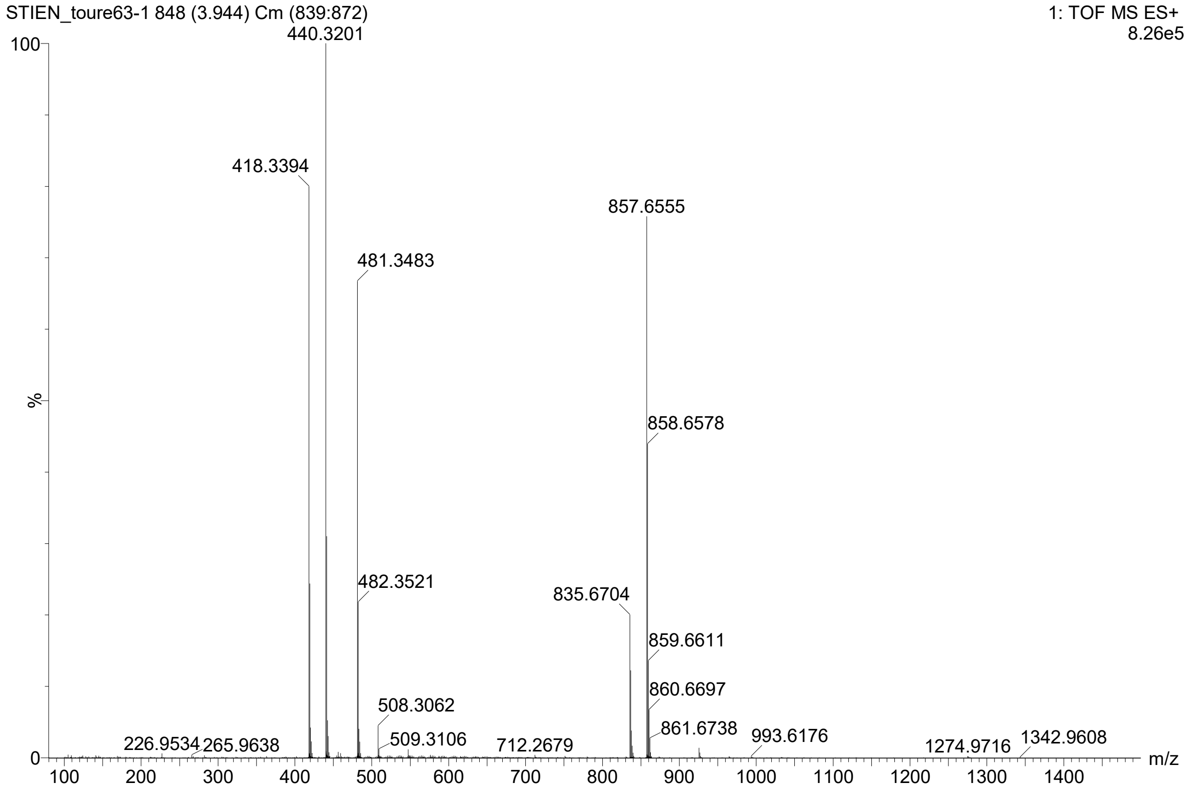

Figure S24. HRMS of compound 3-OMe recorded in MeOH.

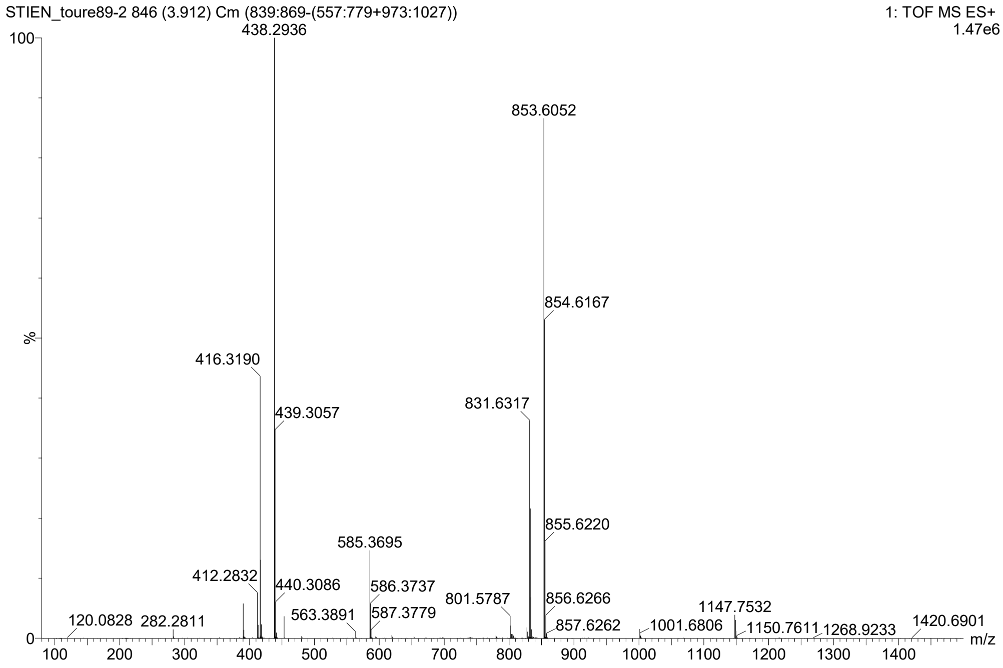

Figure S25. ^{1}H spectrum of 5 recorded at 500 MHz in CD_3OD.

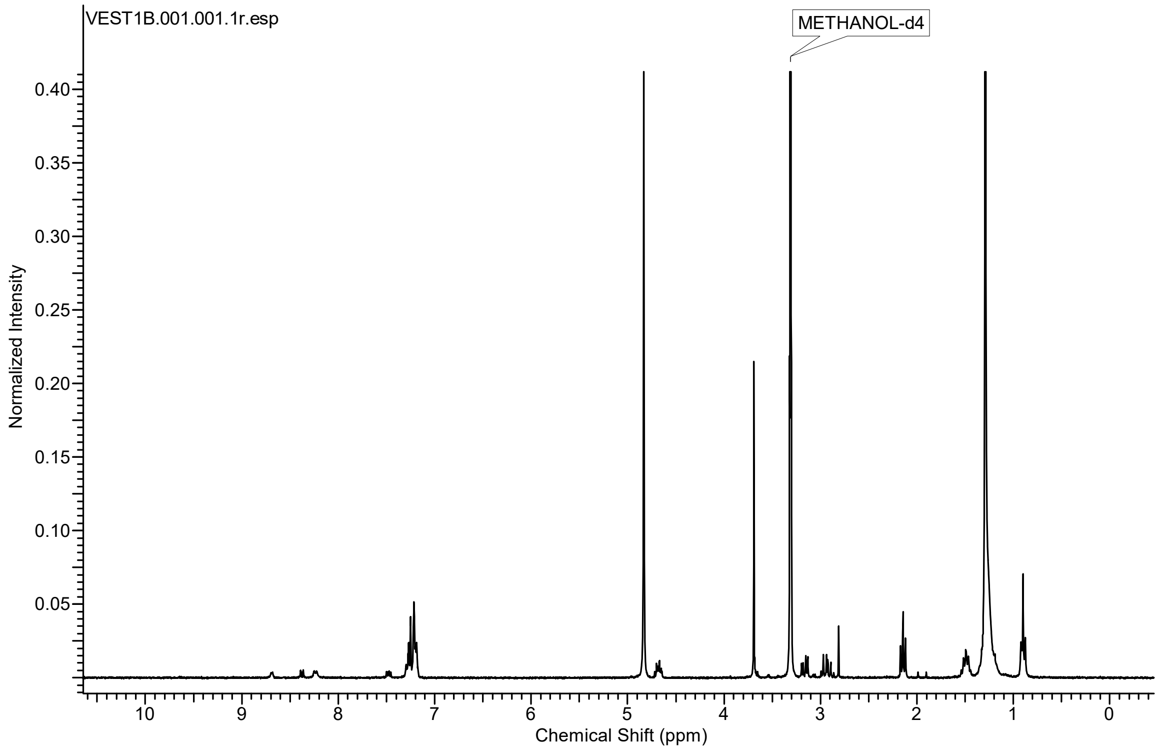

Figure S26. ^{13}C NMR spectrum for compound 5 in CD_3OD

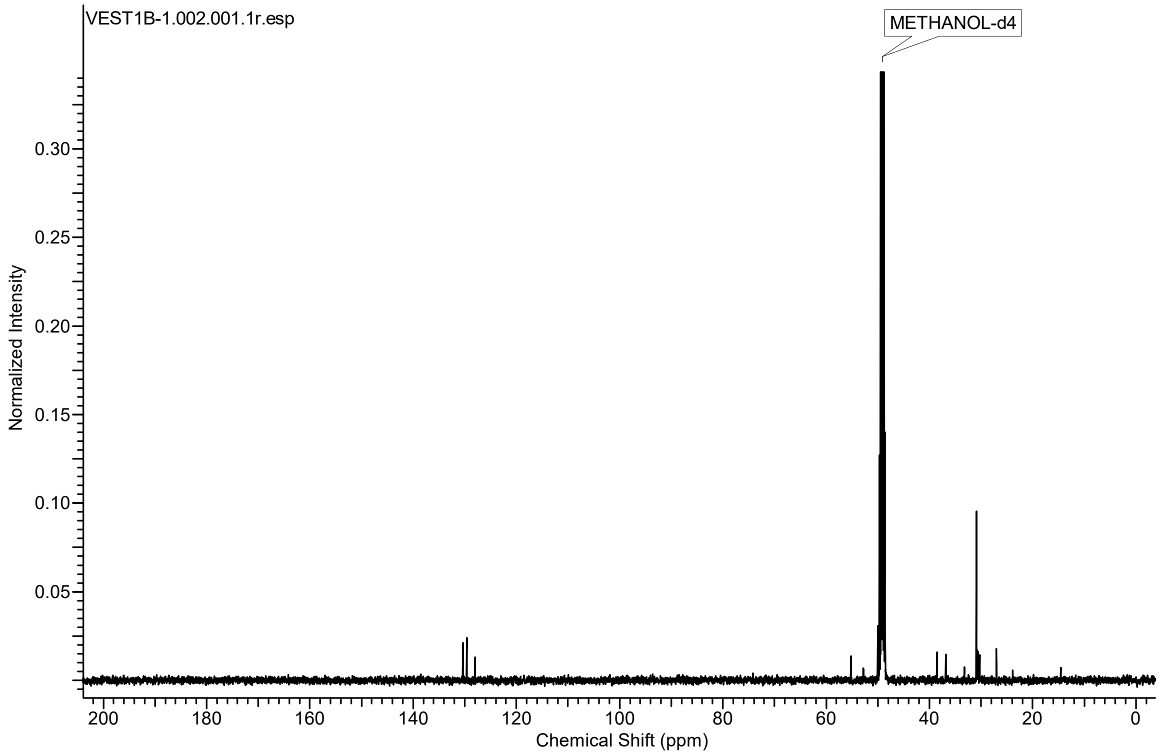

Figure S27. HRMS of compound 5 recorded in MeOH.

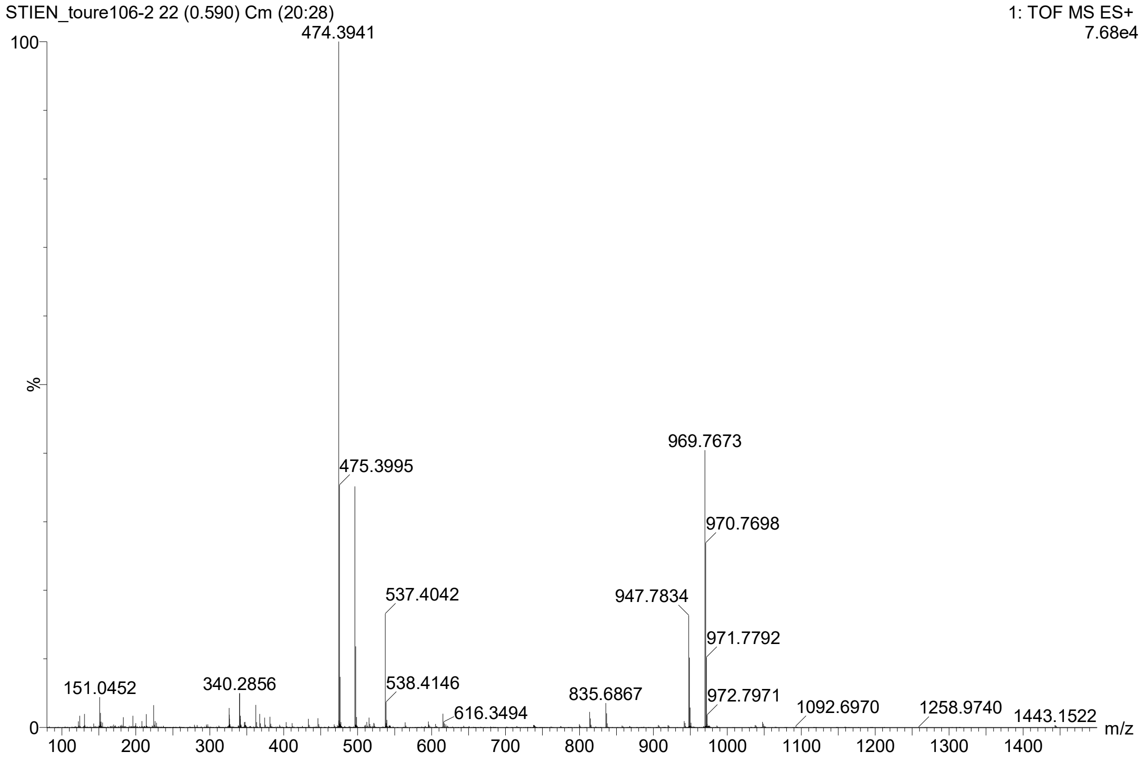

Figure S28. 1H NMR spectrum for compound 6 in CD3OD

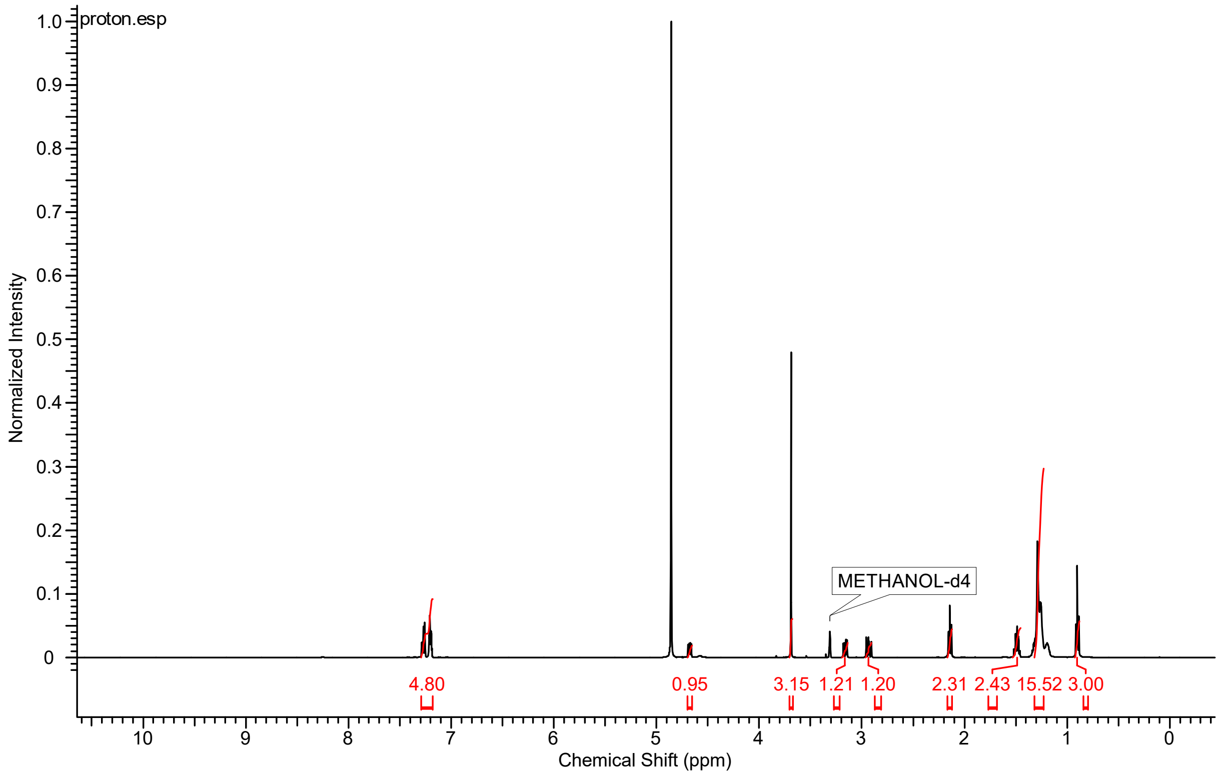

Figure S29. 13C NMR spectrum for compound 6 in CD_3OD

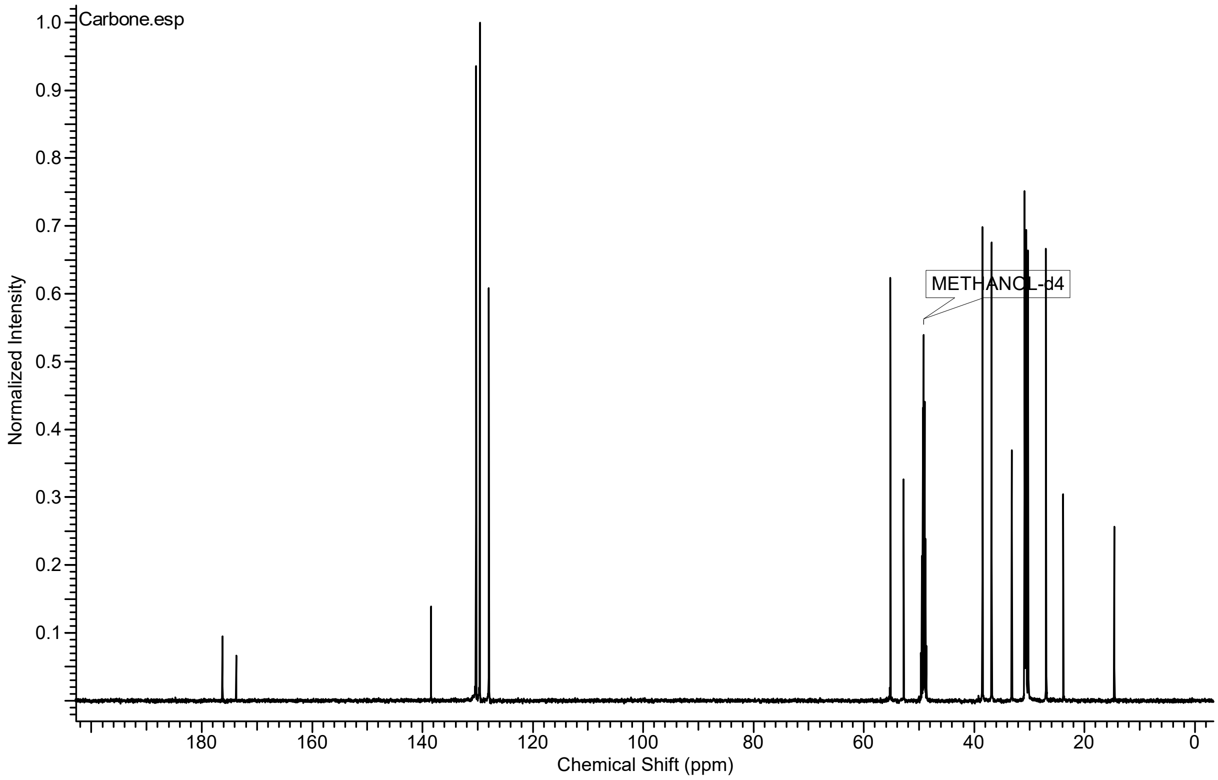

Figure S30. HRMS of compound 6 in MeOH

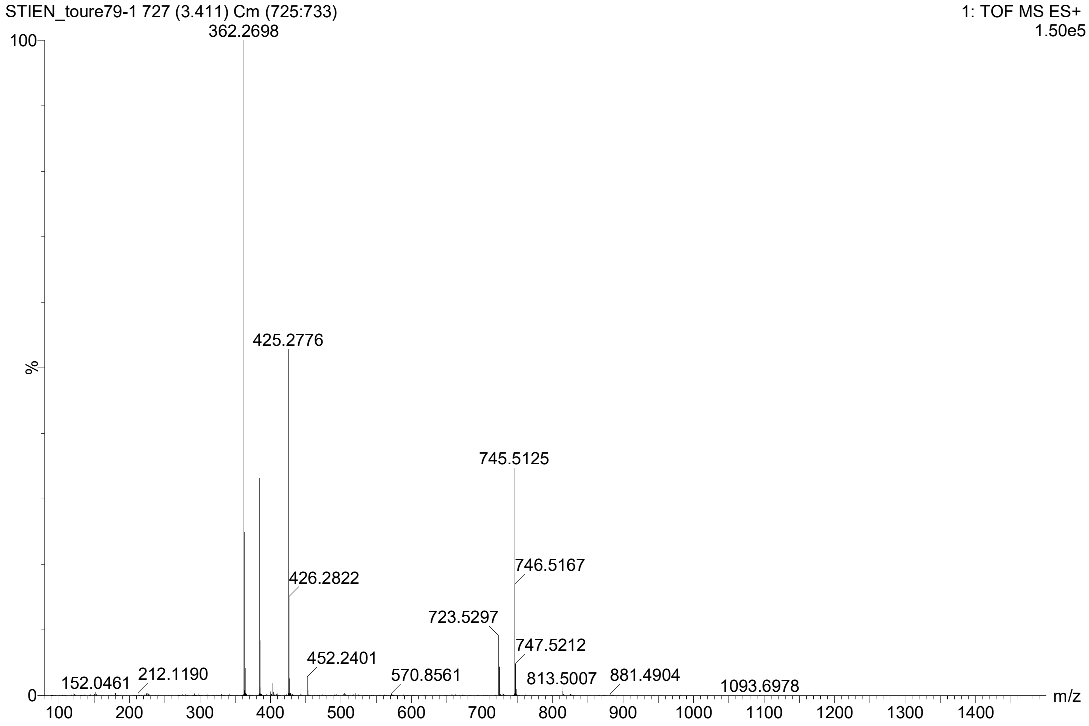

Figure S31. 1H NMR spectrum for compound 7 in CD3OD

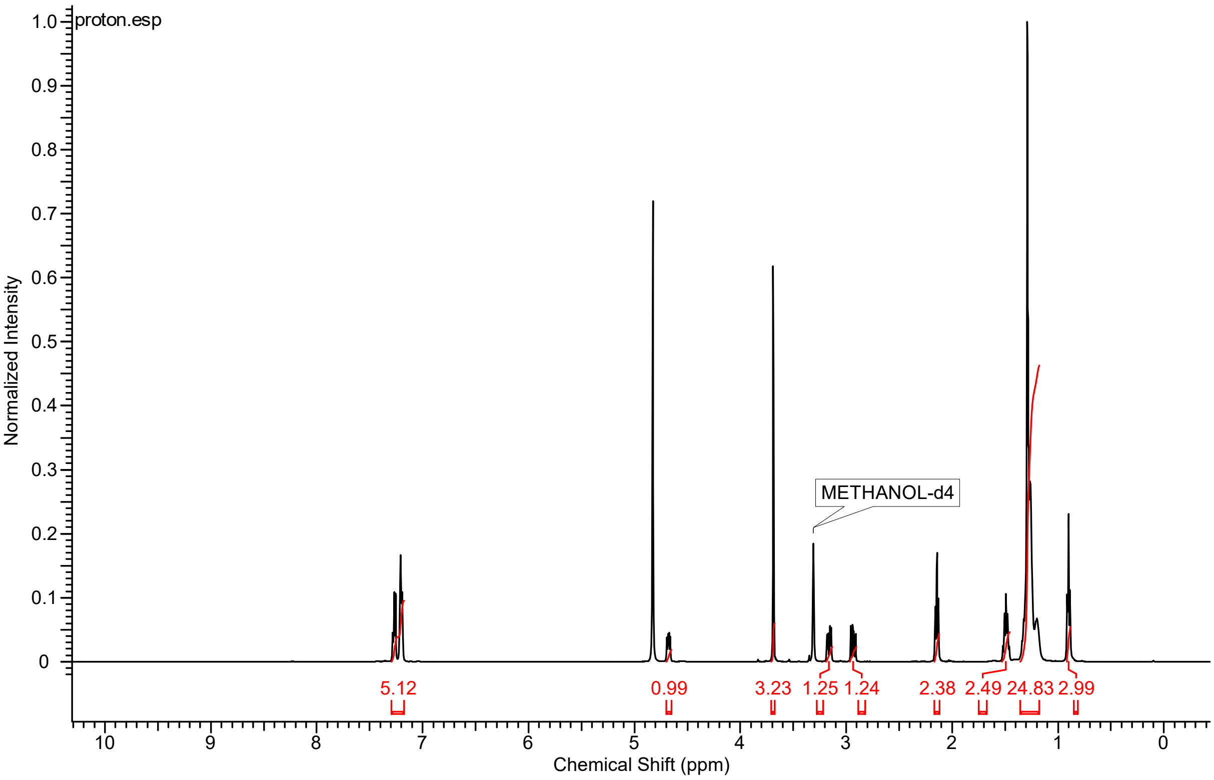

Figure S32. ^{13}C NMR spectrum for compound 7 in CD3OD

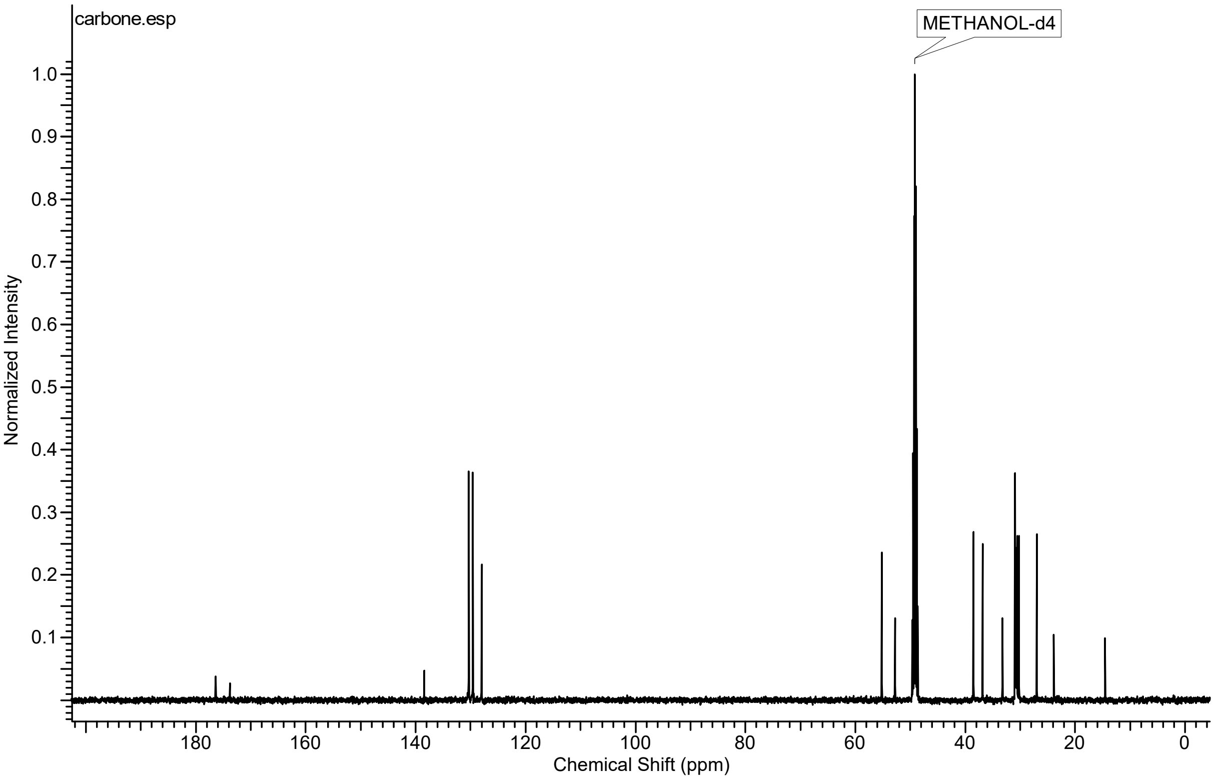

Figure S33. HRMS of compound 7 in MeOH

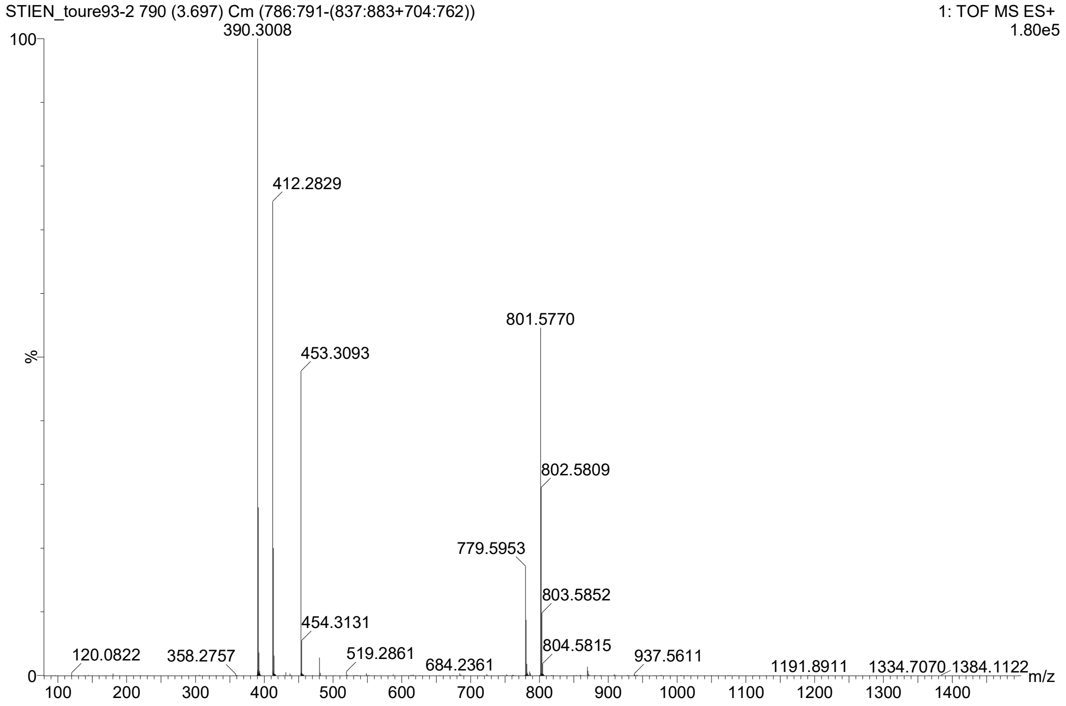

Figure S34. 1H NMR spectrum for compound 8 in CD_3OD

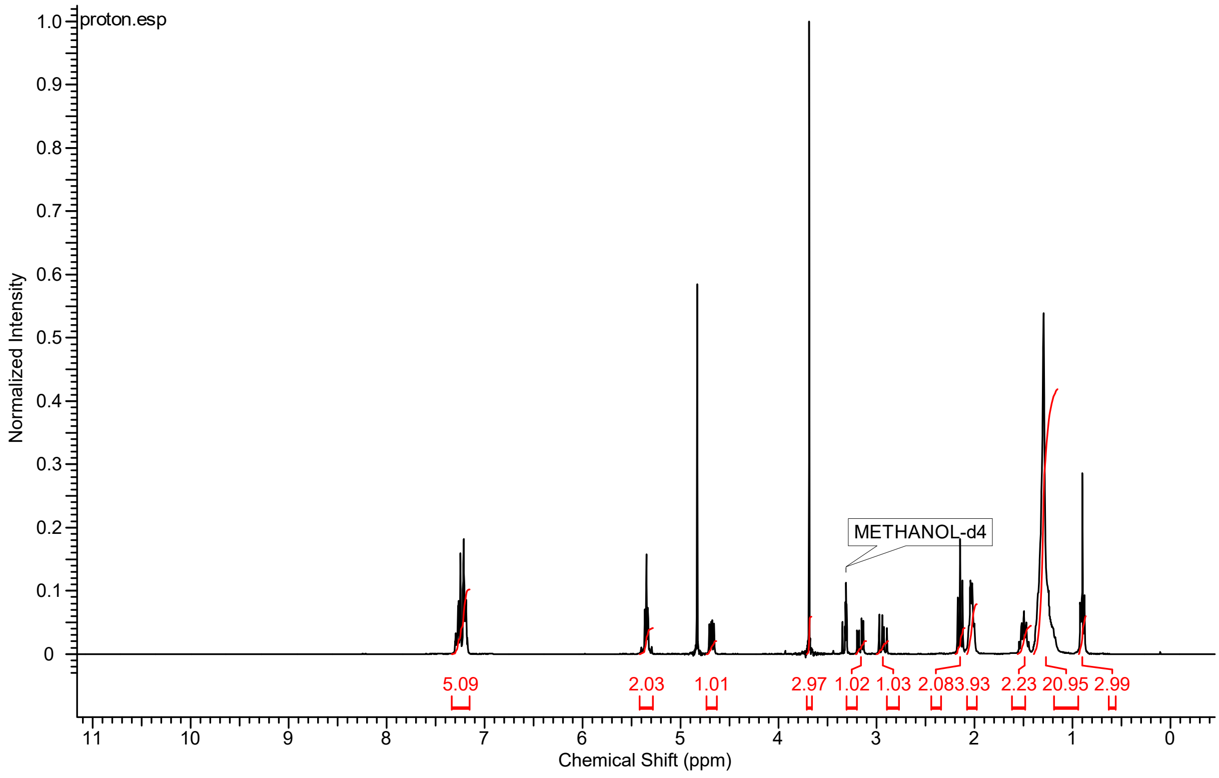

Figure S35. ^{13}C NMR spectrum for compound 8 in CD_3OD

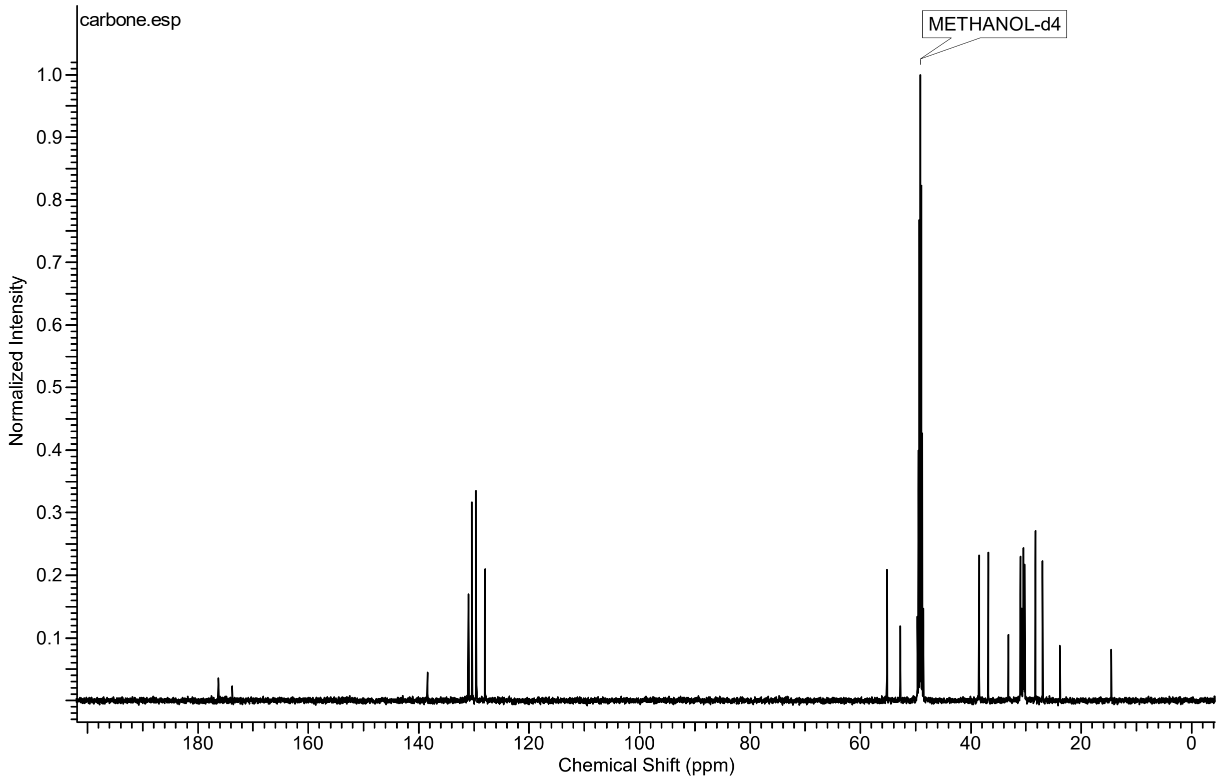

Figure S36. HRMS of compound 8 in MeOH

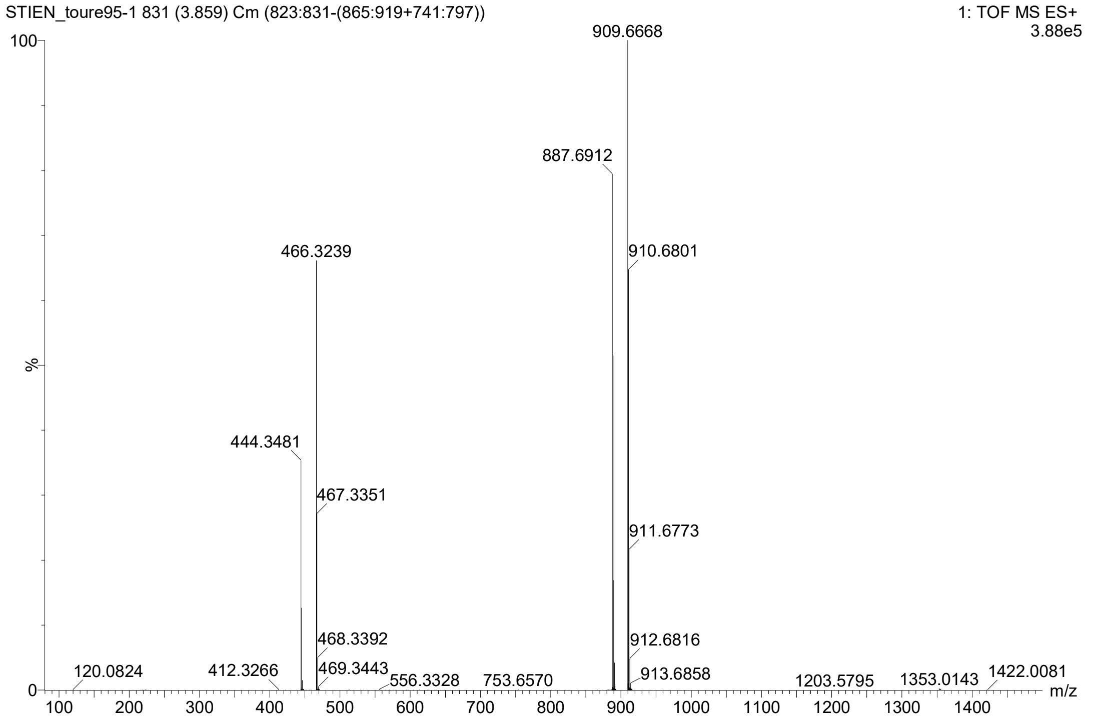

Figure S37. 1H NMR spectrum for compound 9 in CD3OD

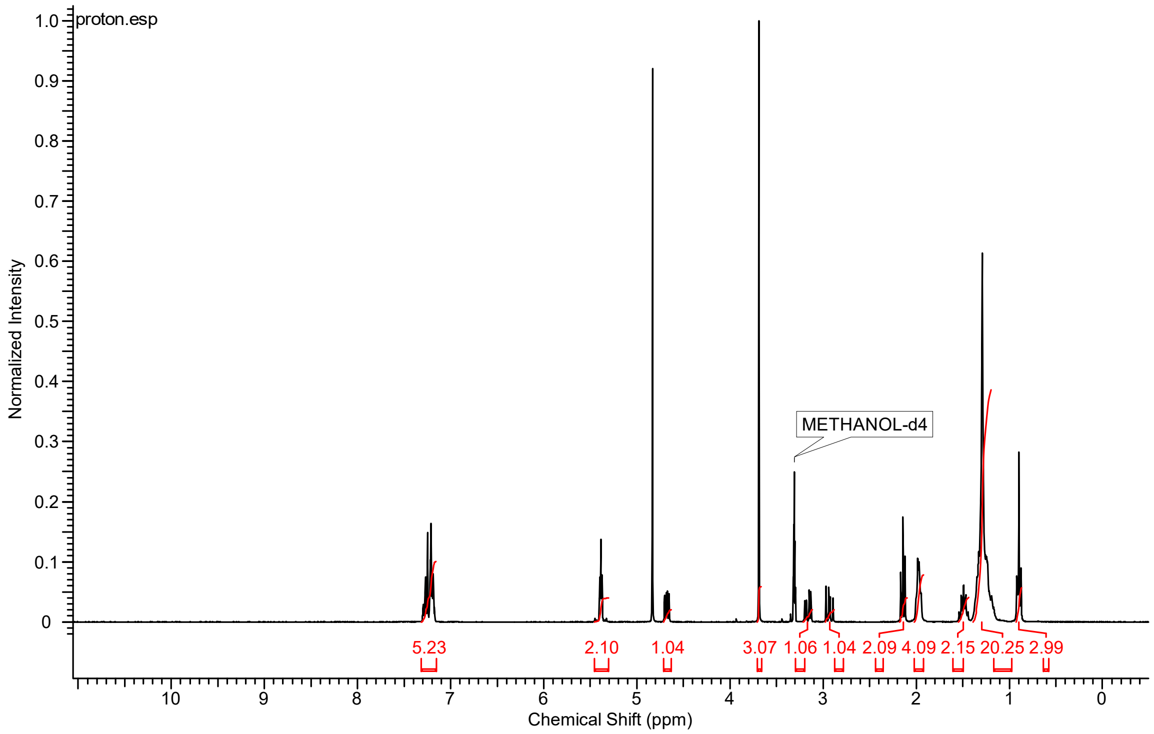

Figure S38. ^{13}C NMR spectrum for compound 9 in CD3OD

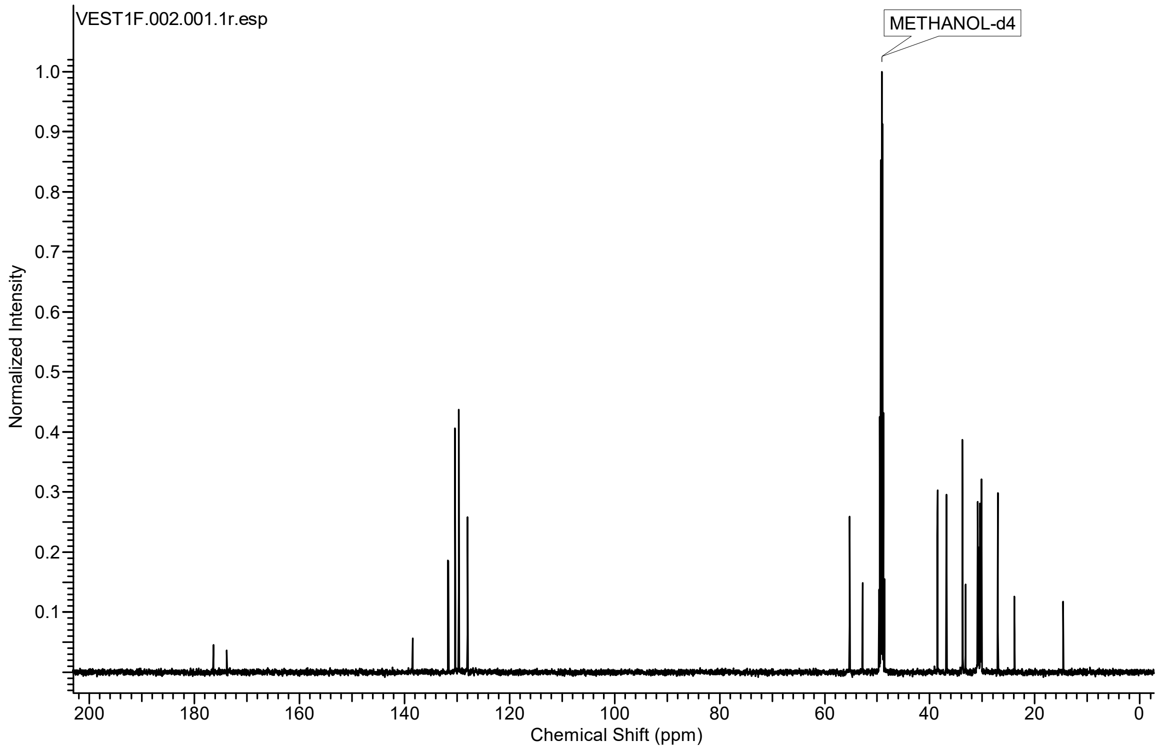

Figure S39. HRMS of compound 9 in MeOH

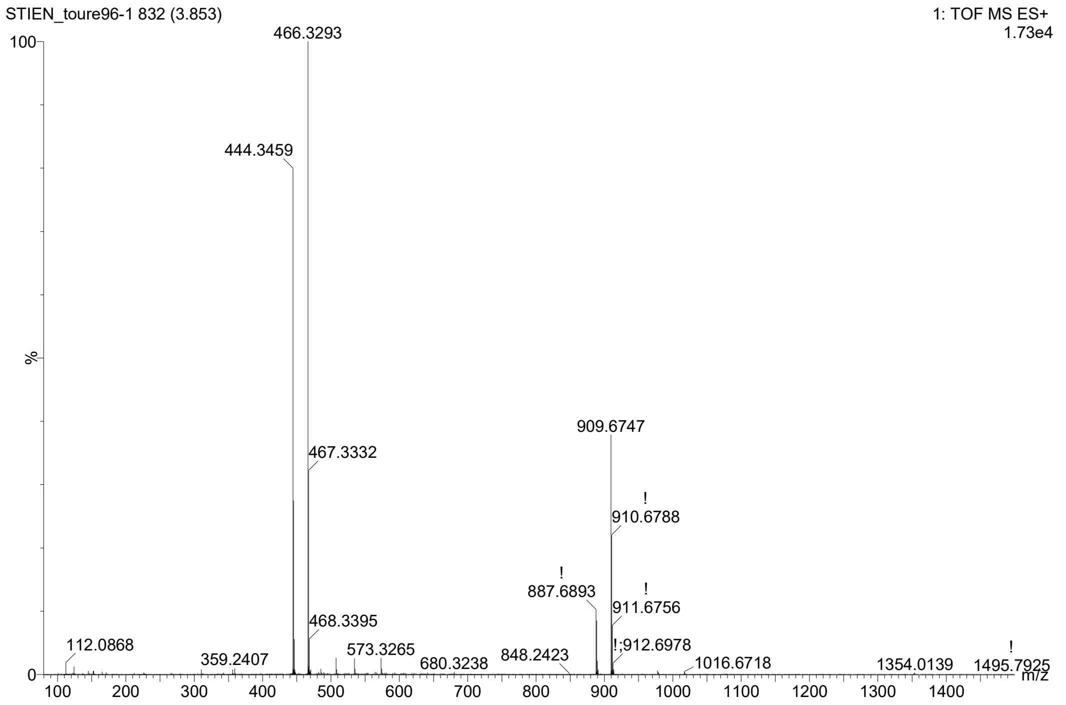

Figure S40. 1H NMR spectrum for compound 10 in CD_3OD

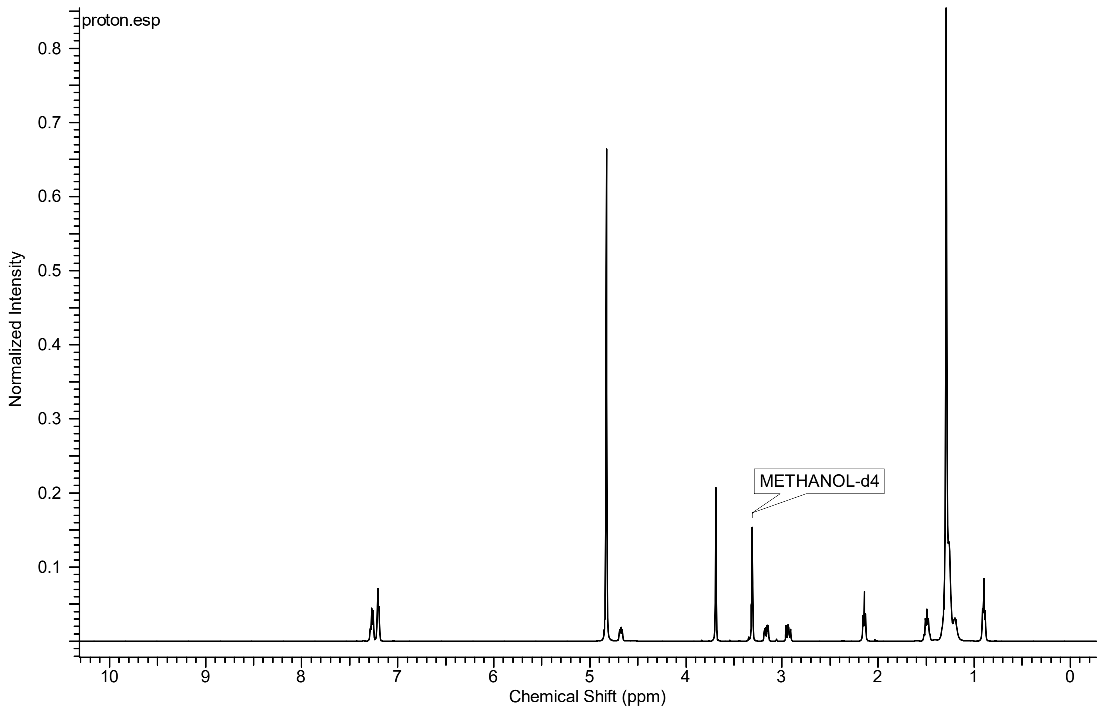

Figure S41. ^{13}C NMR spectrum for compound 10 in CD_3OD

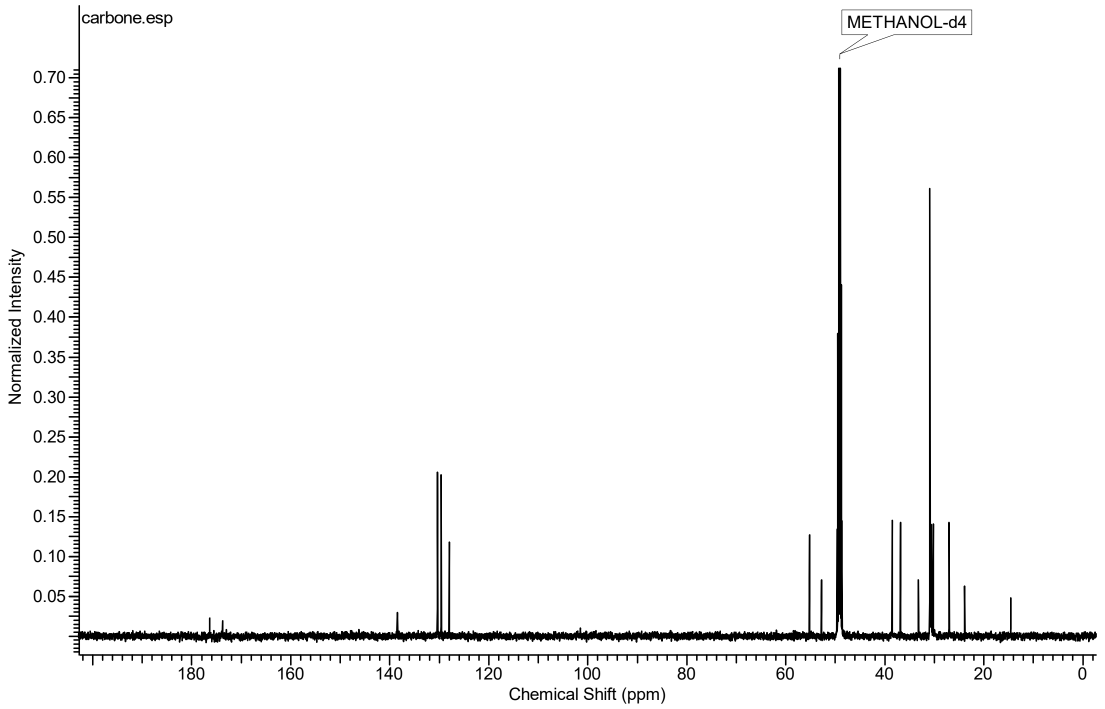

Figure S42. HRMS of compound 10 in MeOH

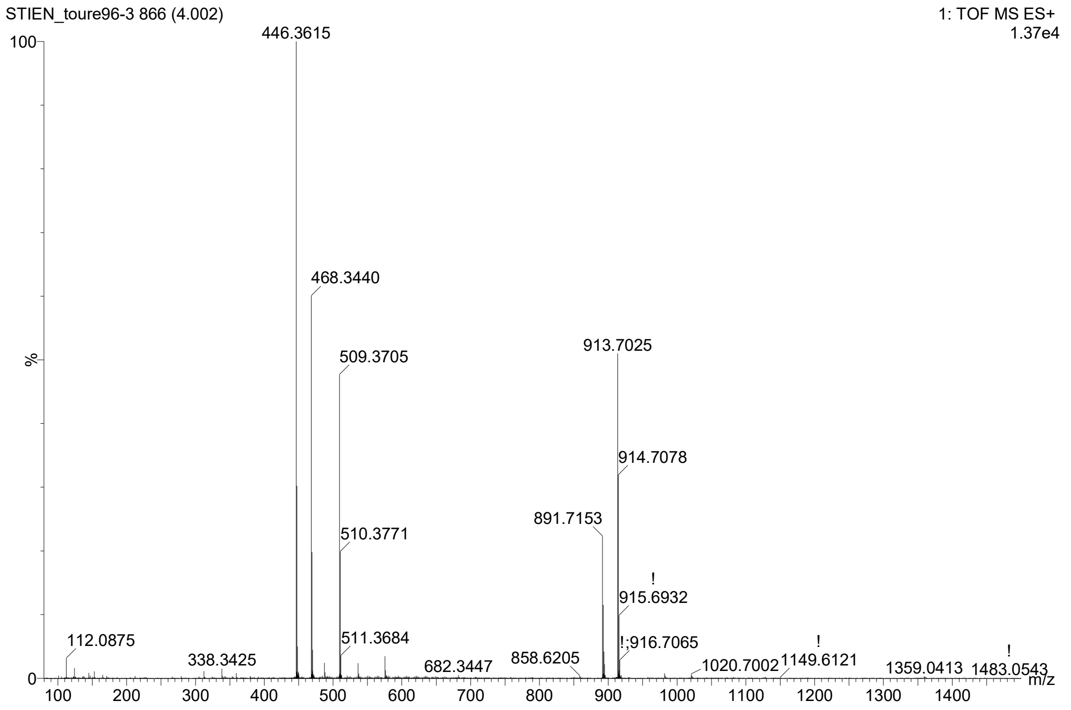

Figure S43. 1H NMR spectrum for compound 11 in CD_3OD

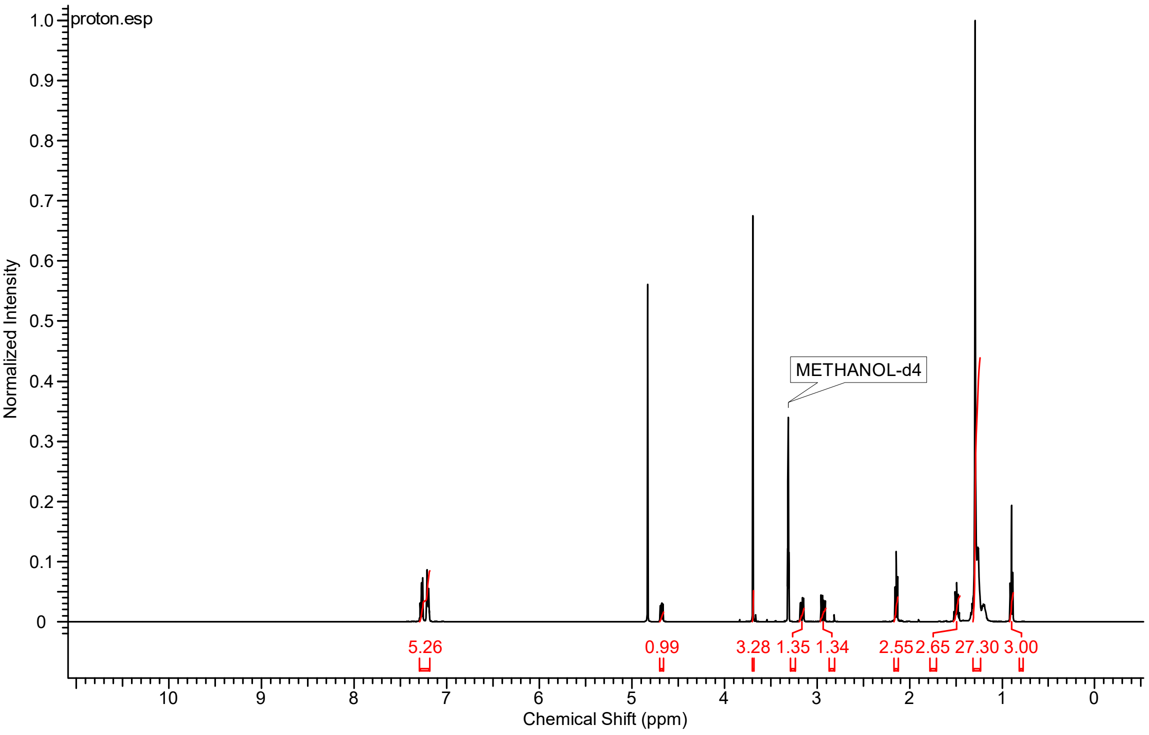

Figure S44. ^{13}C NMR spectrum for compound 11 in CD_3OD

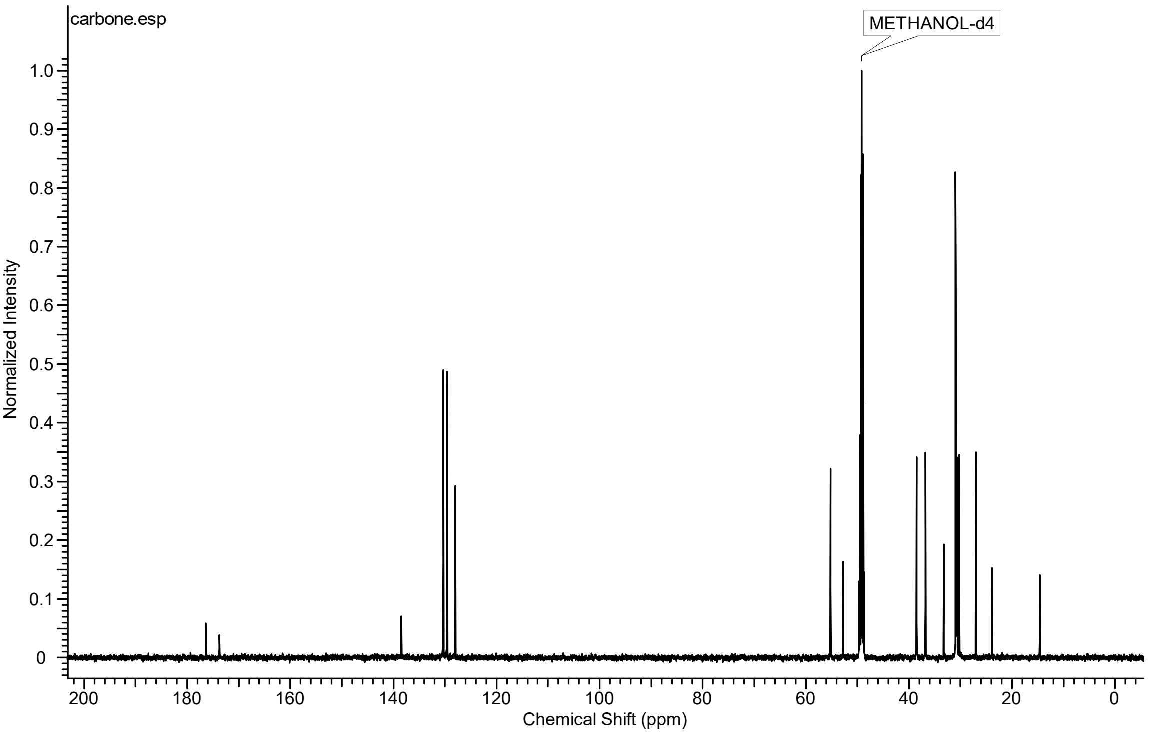

Figure S45. HRMS of compound 11 in MeOH

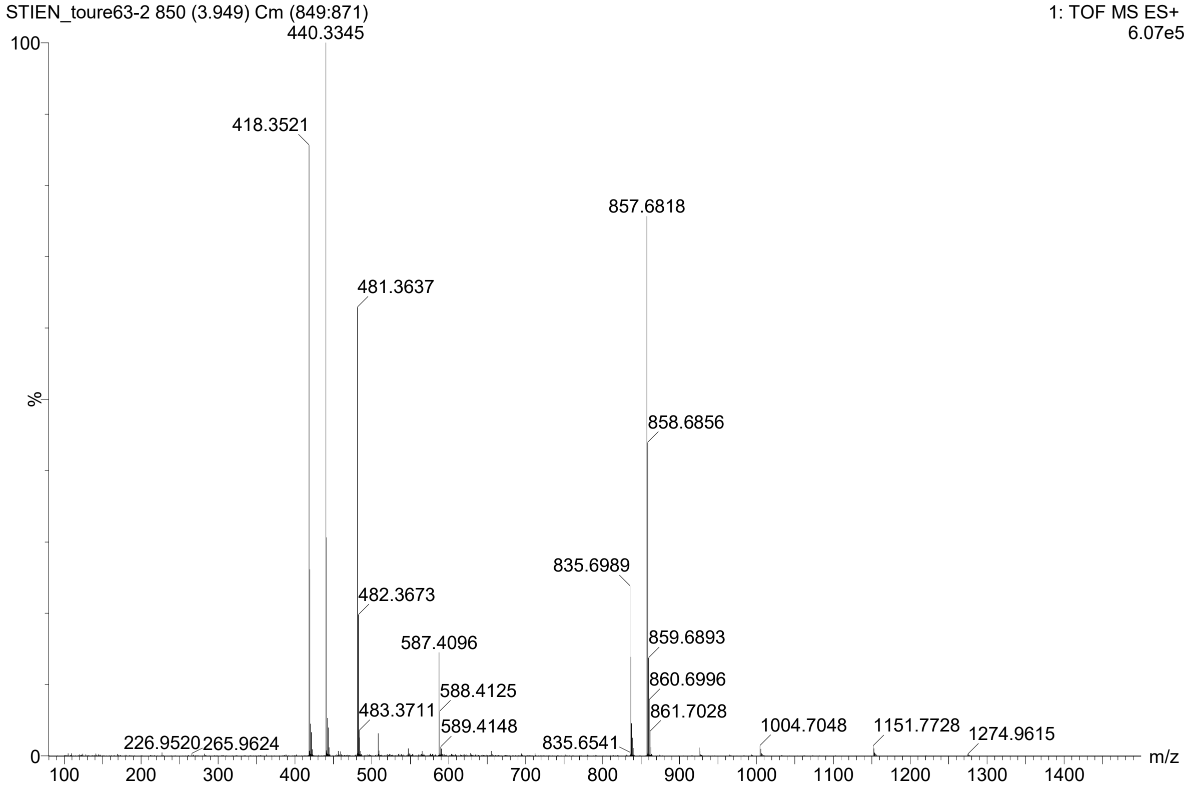

Figure S46. 1H NMR spectrum for compound *ent*-3OMe in CD_3OD

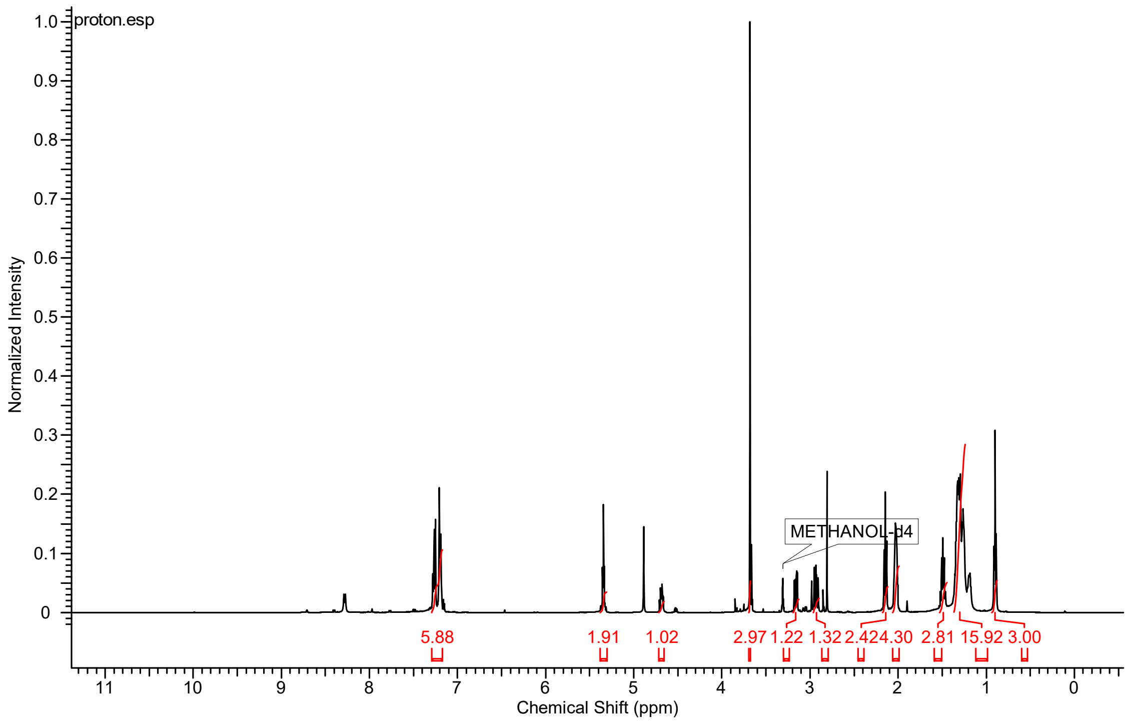

Figure S47. ^{13}C NMR spectrum for compound *ent*-3OMe in CD_3OD

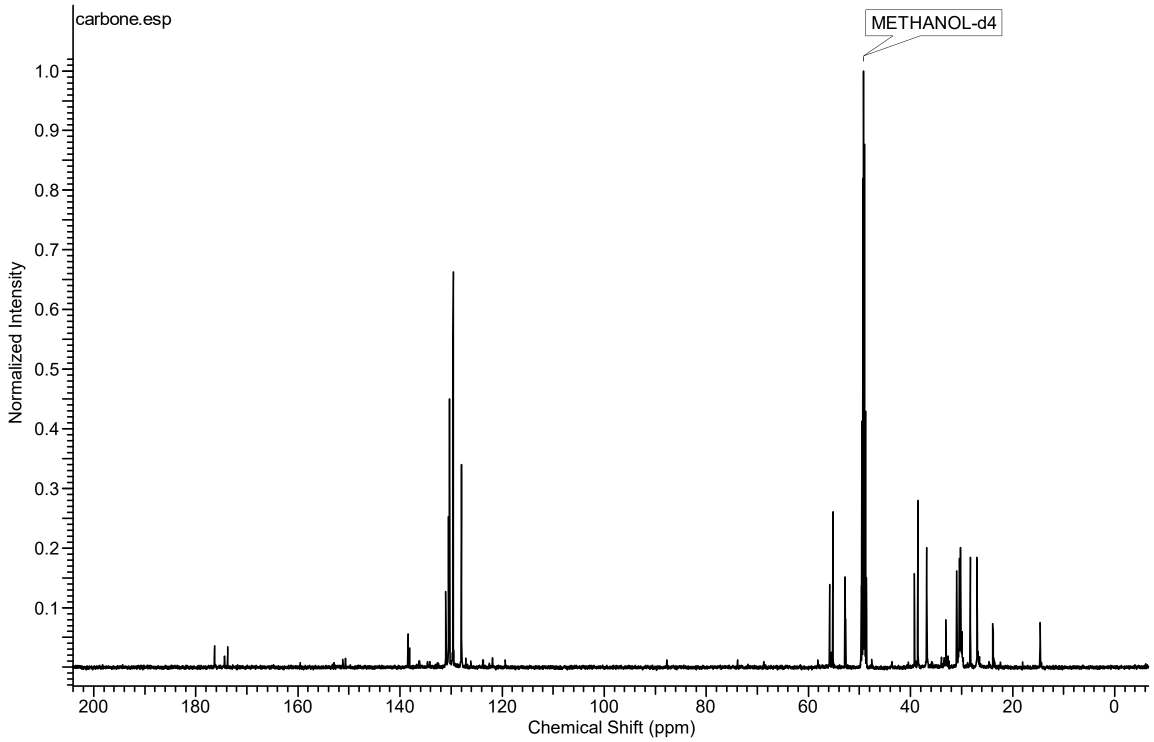

Figure S48. HRMS of compound *ent*-3OMe in MeOH

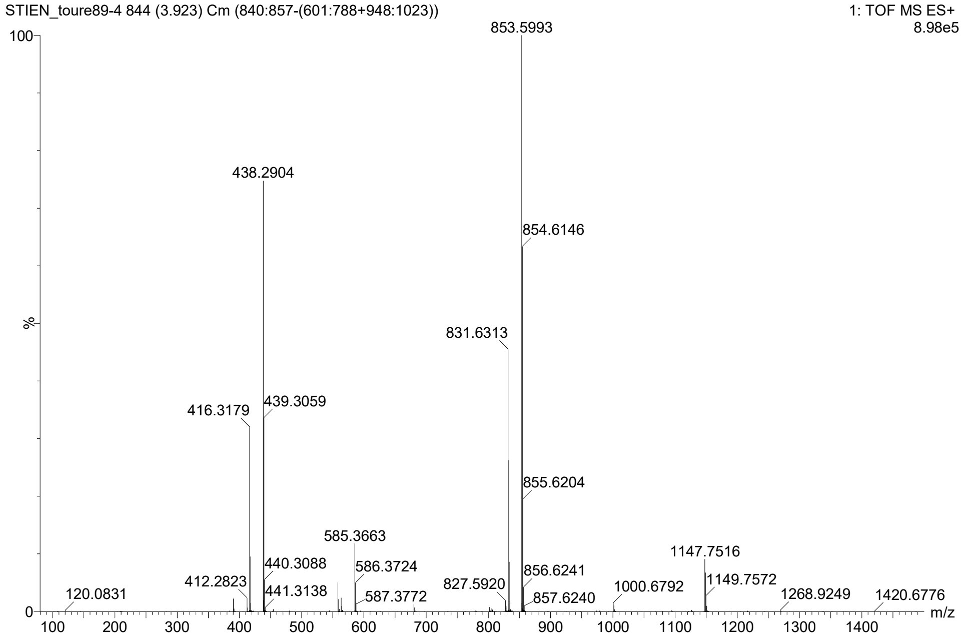

Figure S49. ^{1}H NMR spectrum for compound 12 in CD_3OD

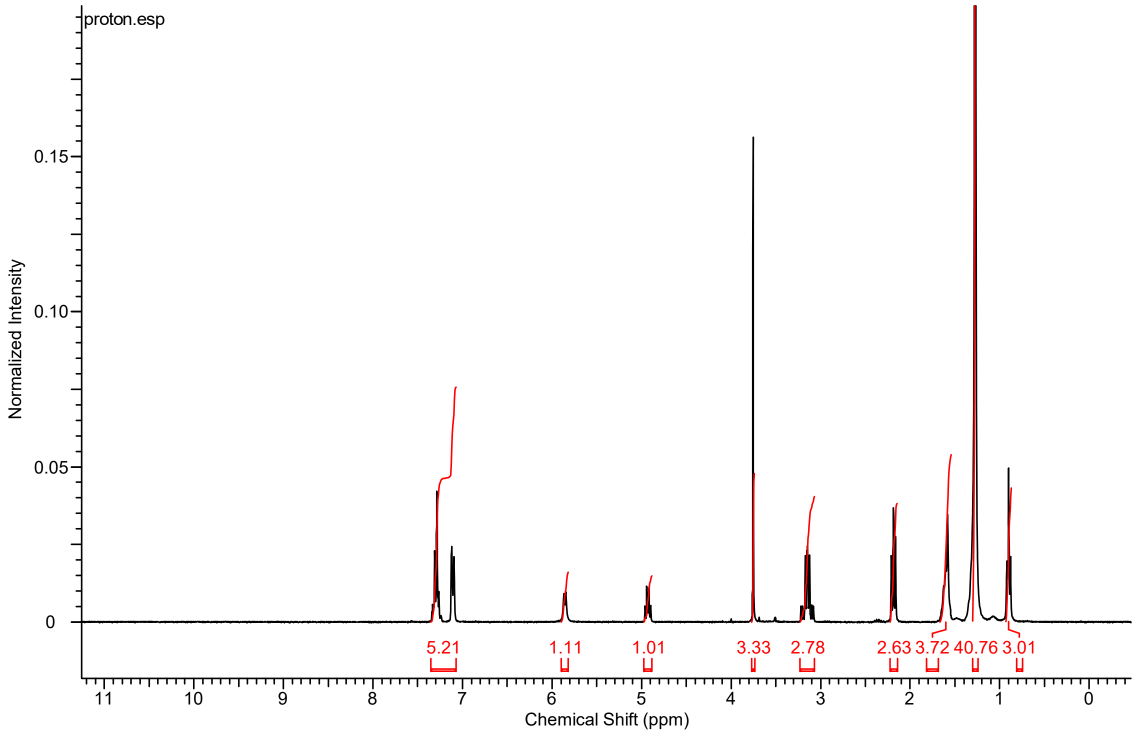

Figure S50. ^{13}C NMR spectrum for compound 12 in CD_3OD

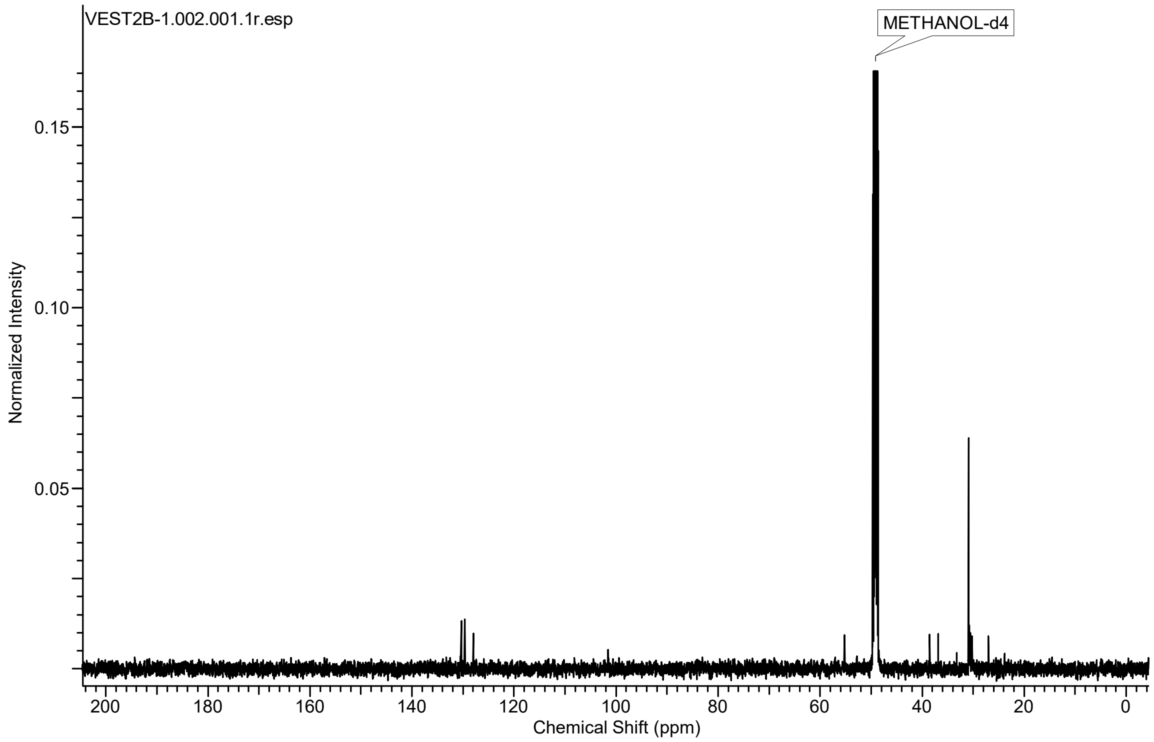

Figure S51. HRMS of compound 12 in MeOH

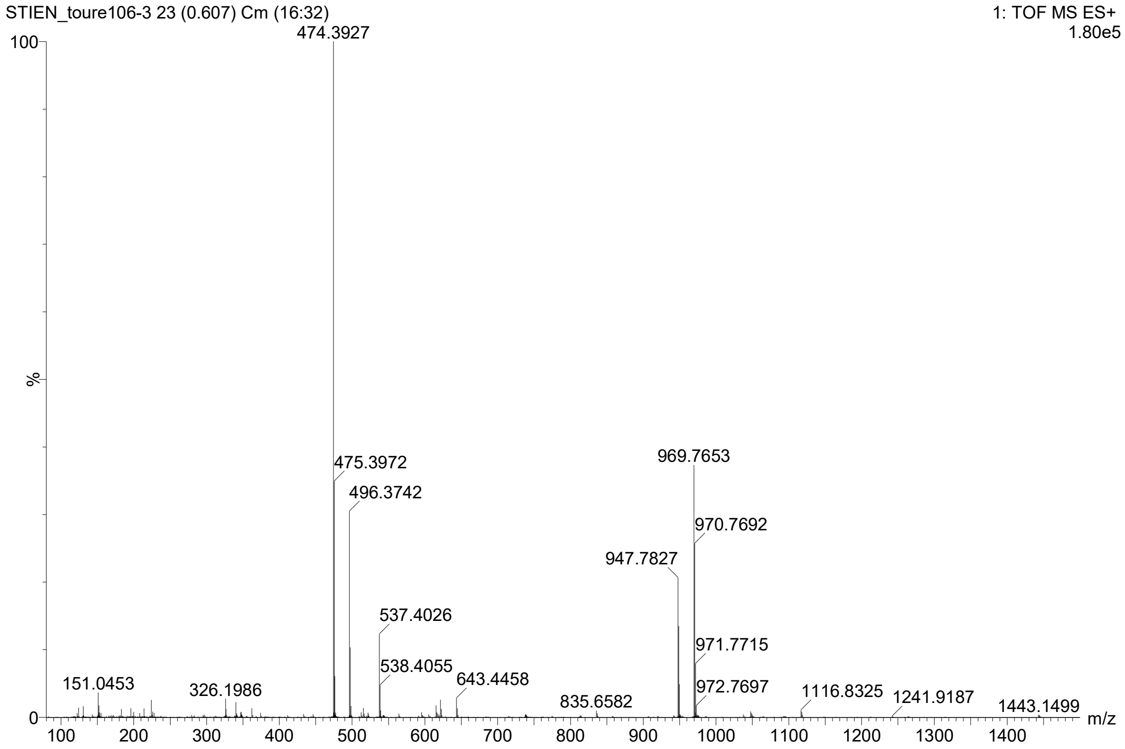

Figure S52. 1H NMR spectrum for compound 13 in CD3OD

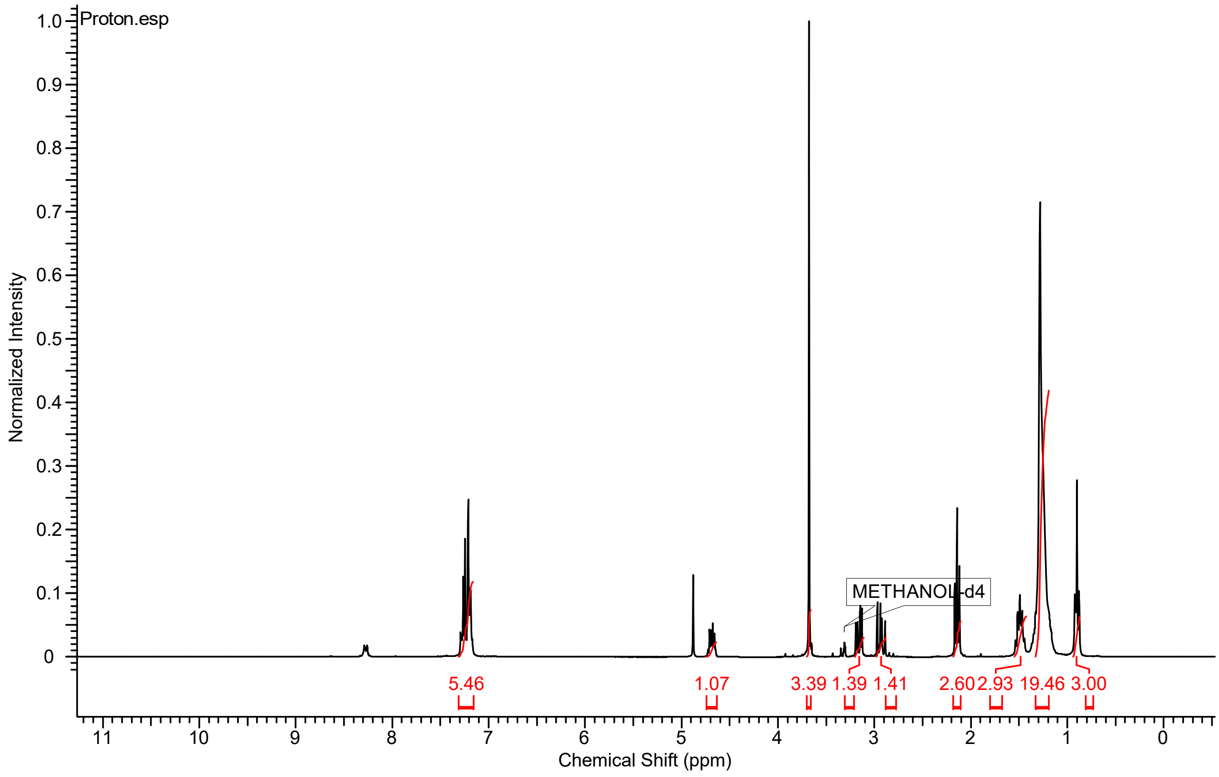

Figure S53. ^{13}C NMR spectrum for compound 13 in CD_3OD

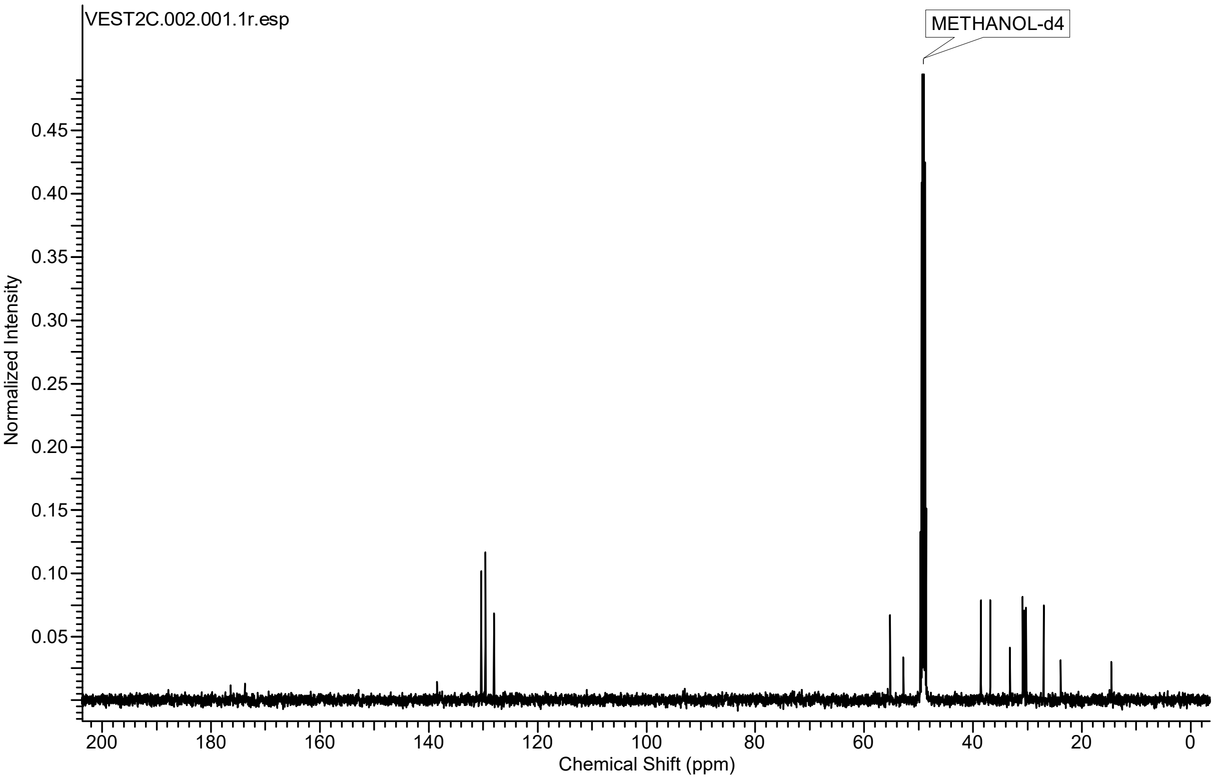

Figure S54. HRMS of compound 13 in MeOH

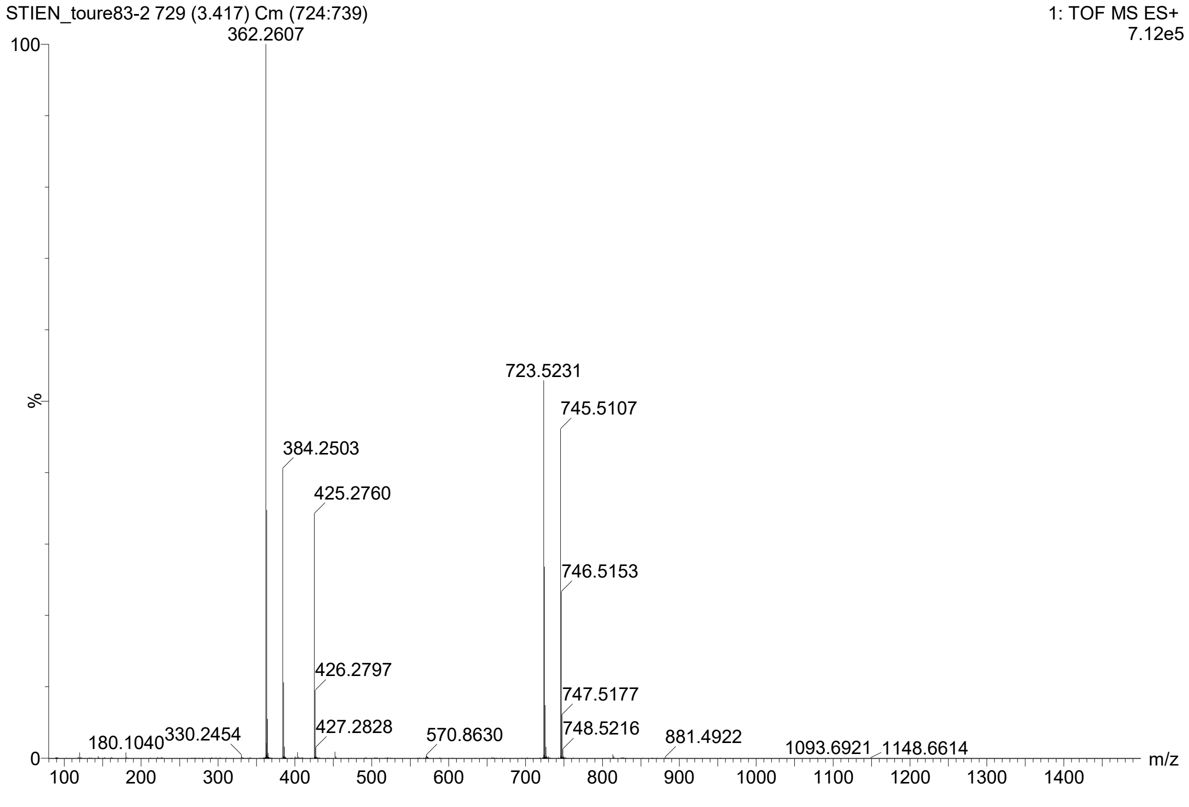

Figure S55. 1H NMR spectrum for compound 14 in CD_3OD

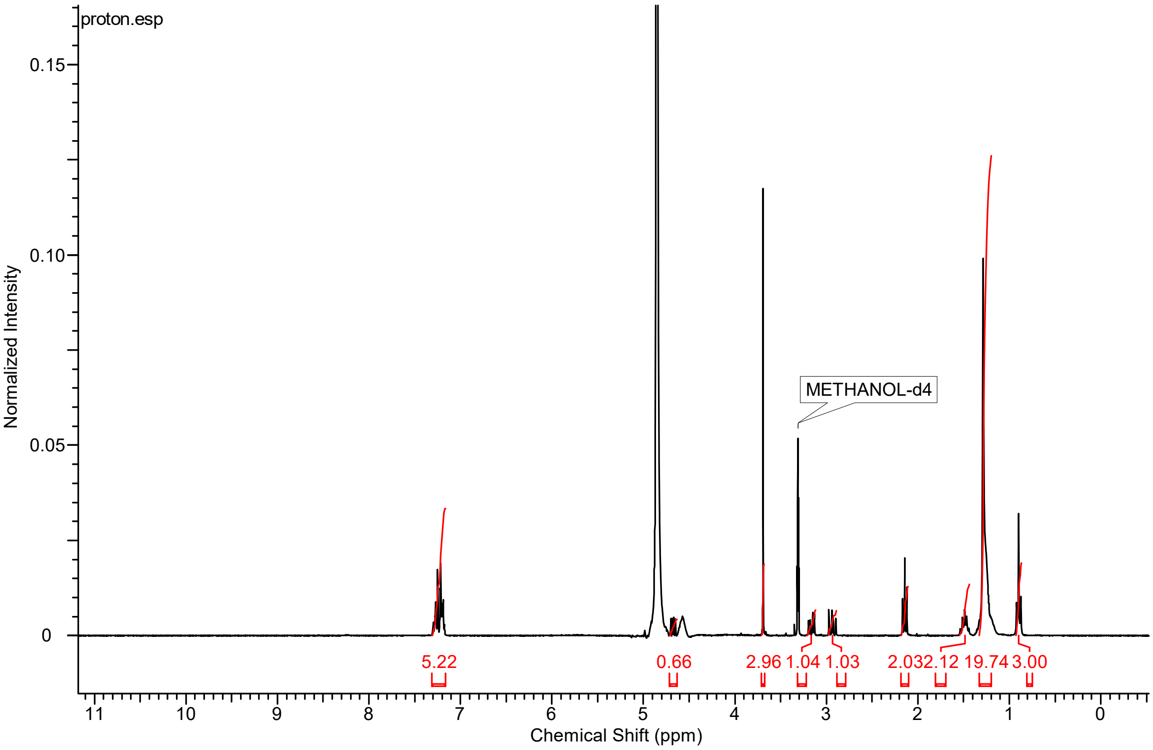

Figure S56. 13C NMR spectrum for compound 14 in CD3OD

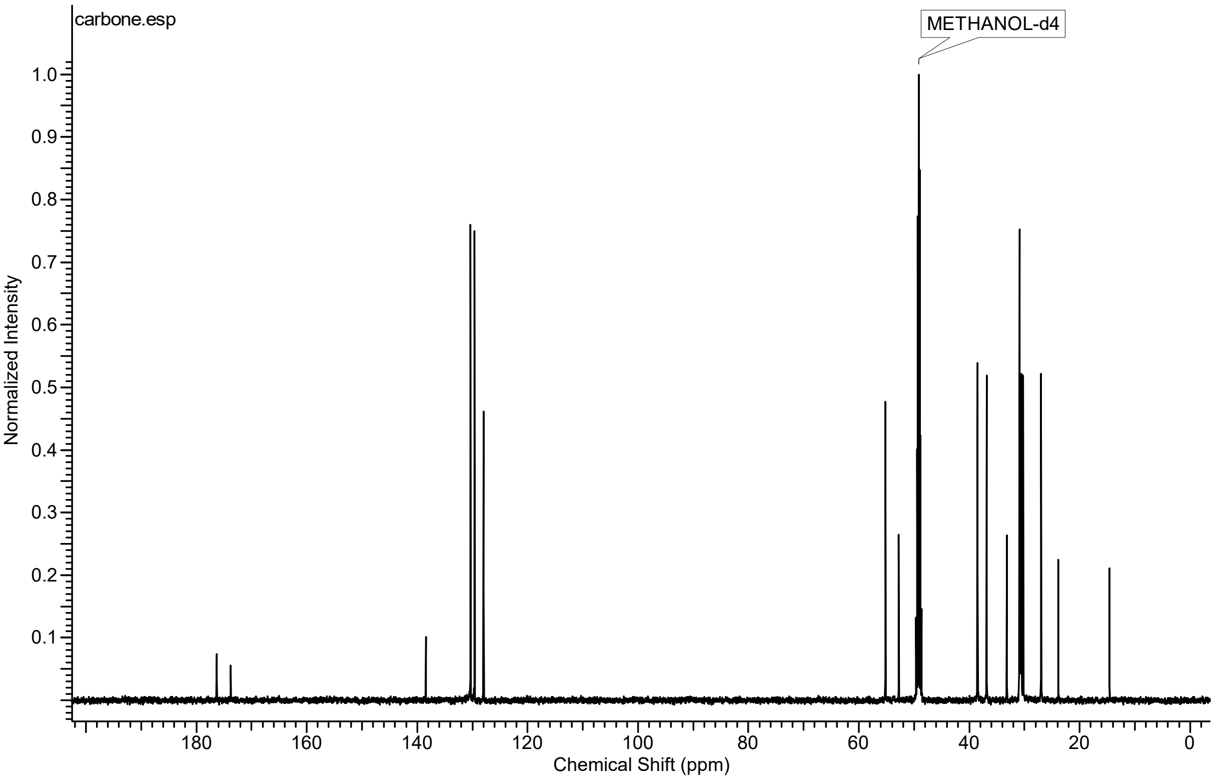

Figure S57. HRMS of compound 14 in MeOH

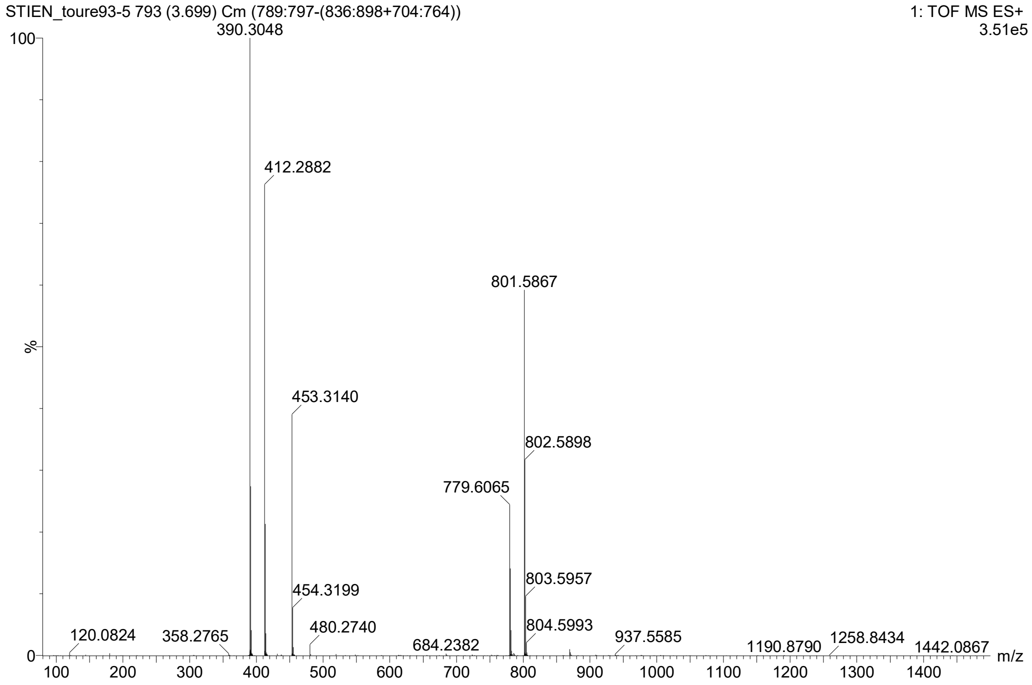

Figure S58. 1H NMR spectrum for compound 15 in CD3OD

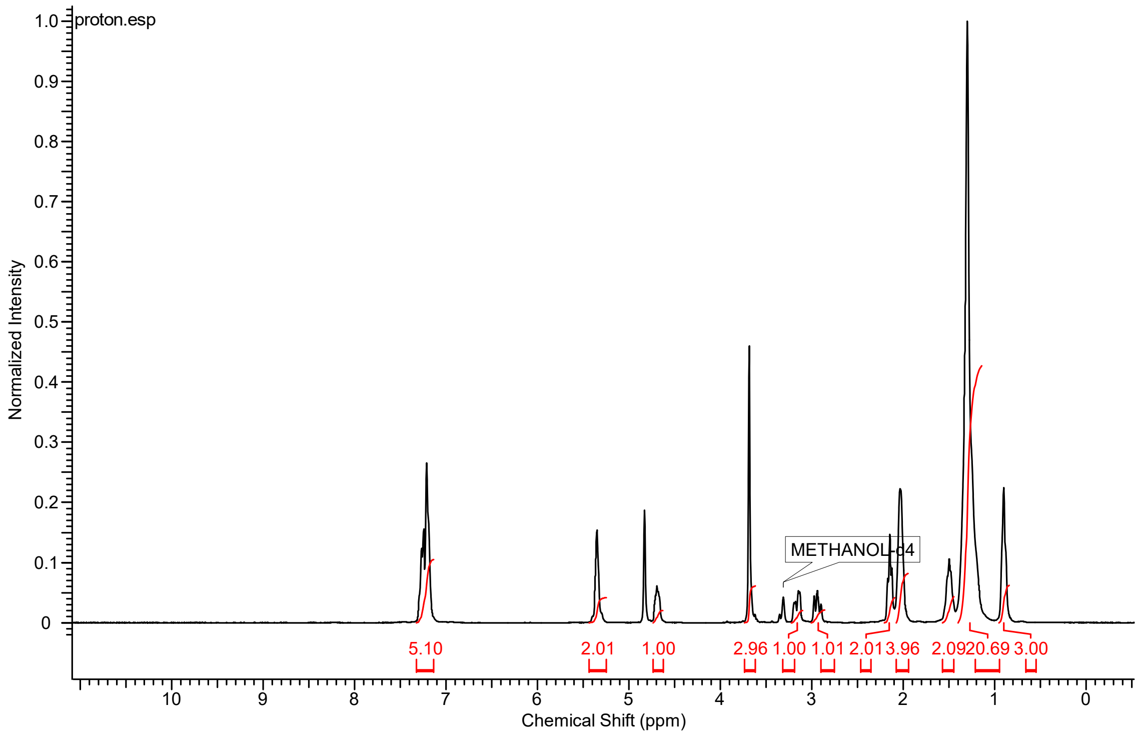

Figure S59. ^{13}C NMR spectrum for compound 15 in CD_3OD

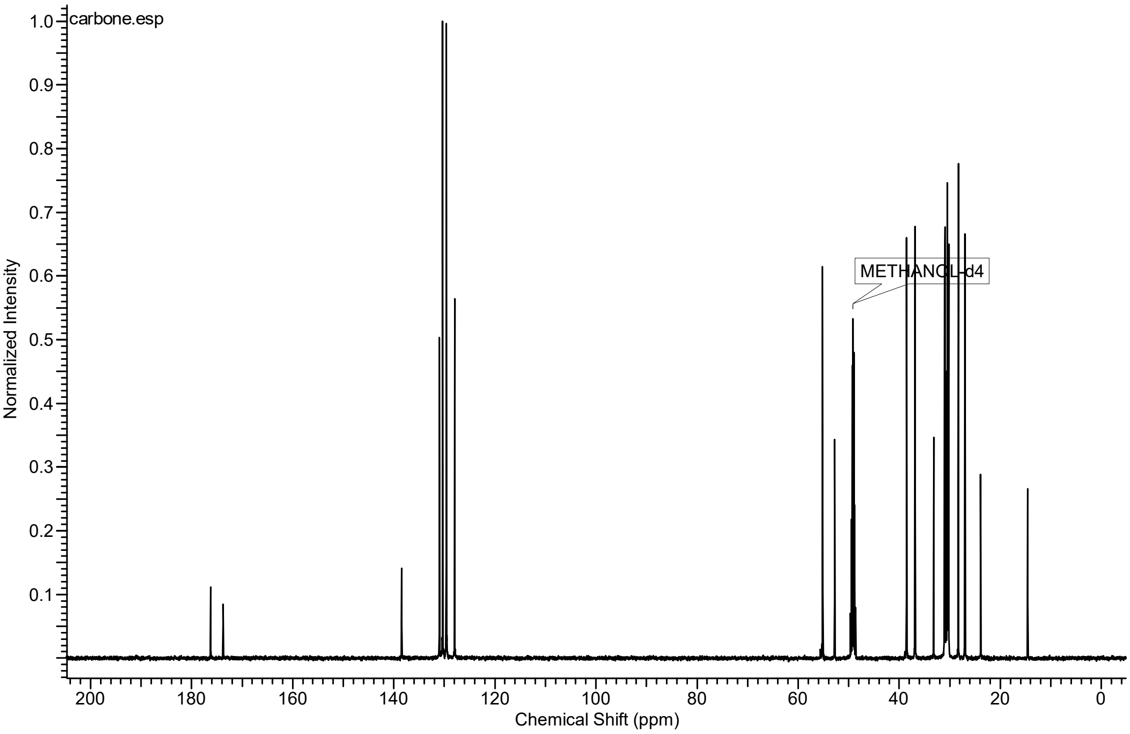

Figure S60. HRMS of compound 15 in MeOH

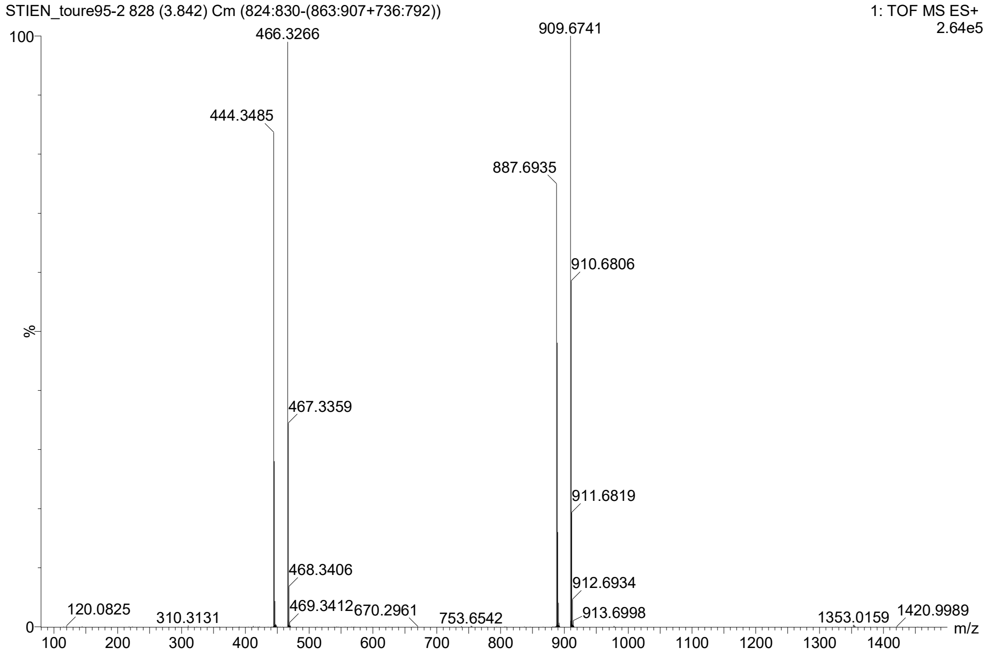

Figure S61. 1H NMR spectrum for compound 16 in CD3OD

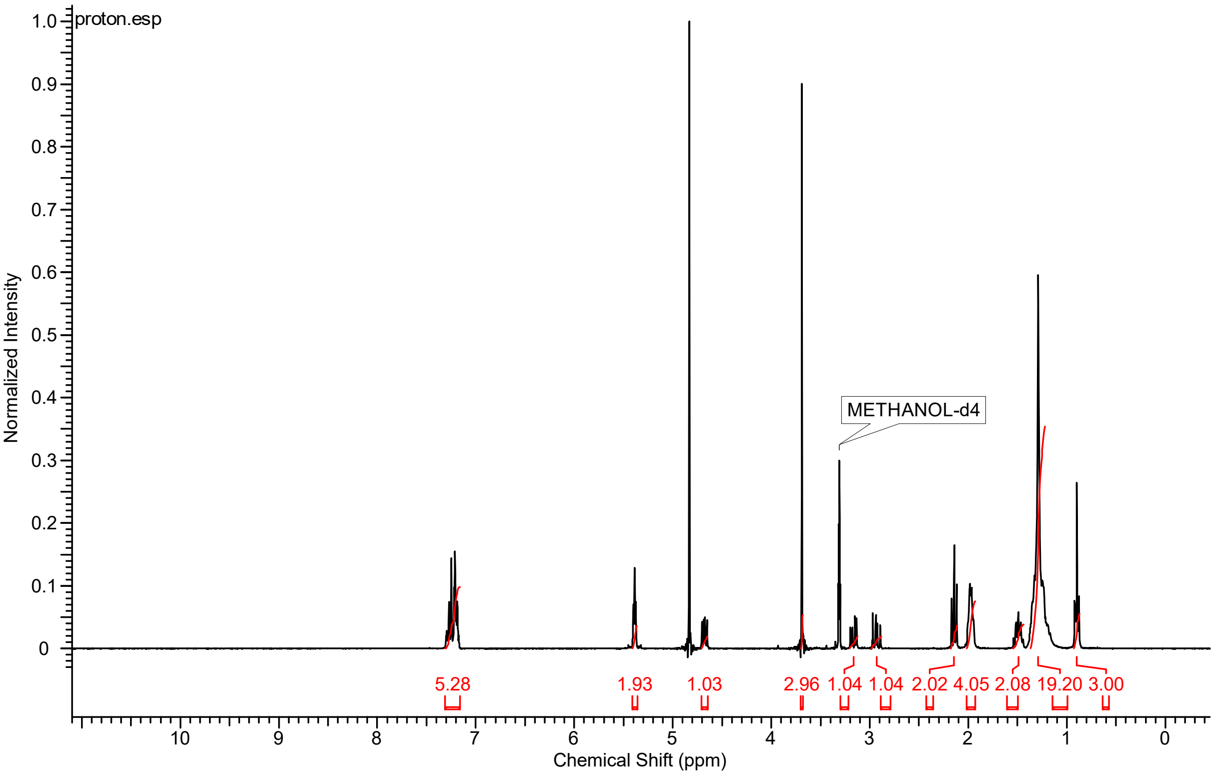

Figure S62. ^{13}C NMR spectrum for compound 16 in CD3OD

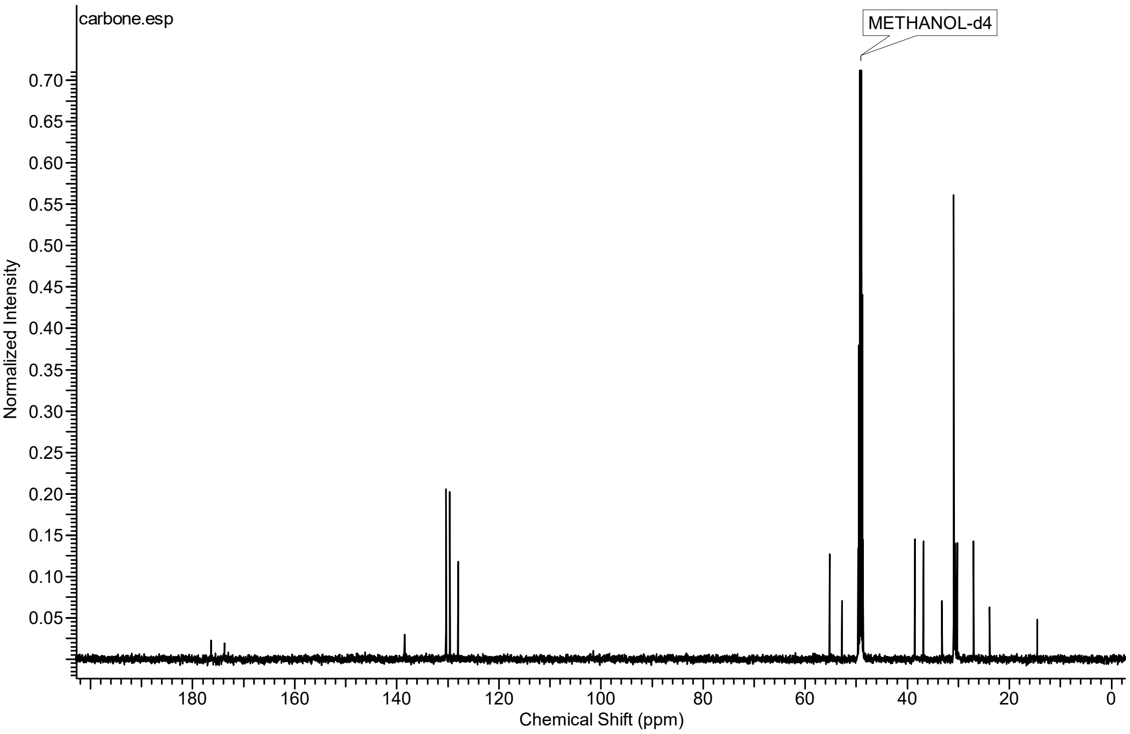

Figure S63. HRMS of compound 16 in MeOH

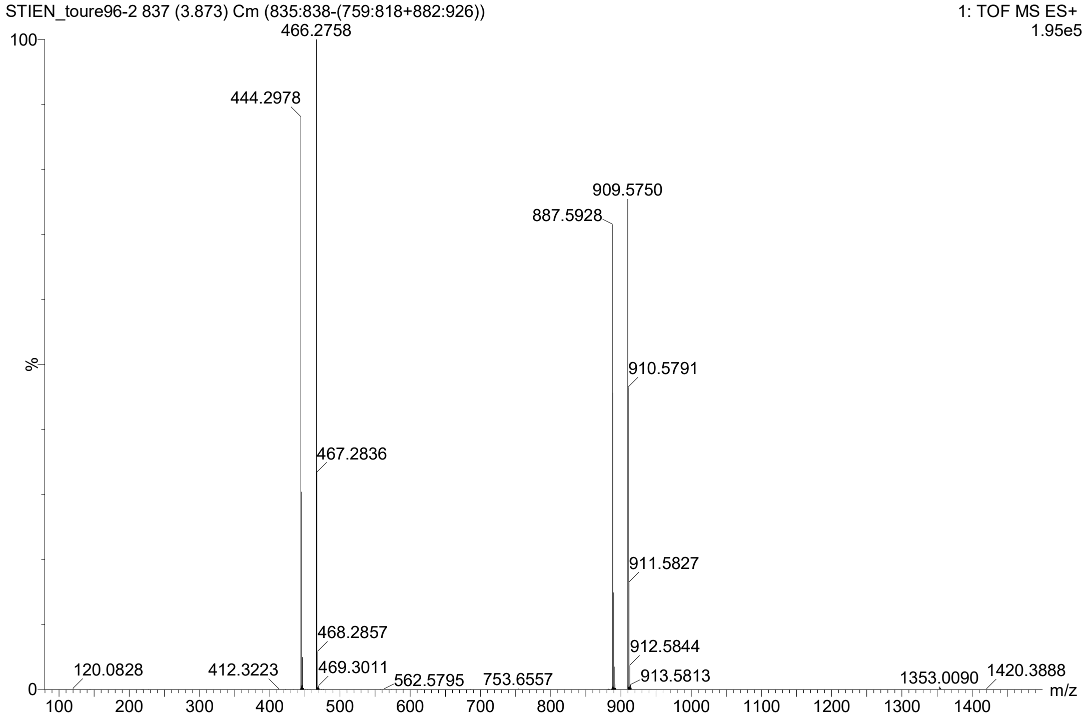

Figure S64. 1H NMR spectrum for compound 17 in CD_3OD

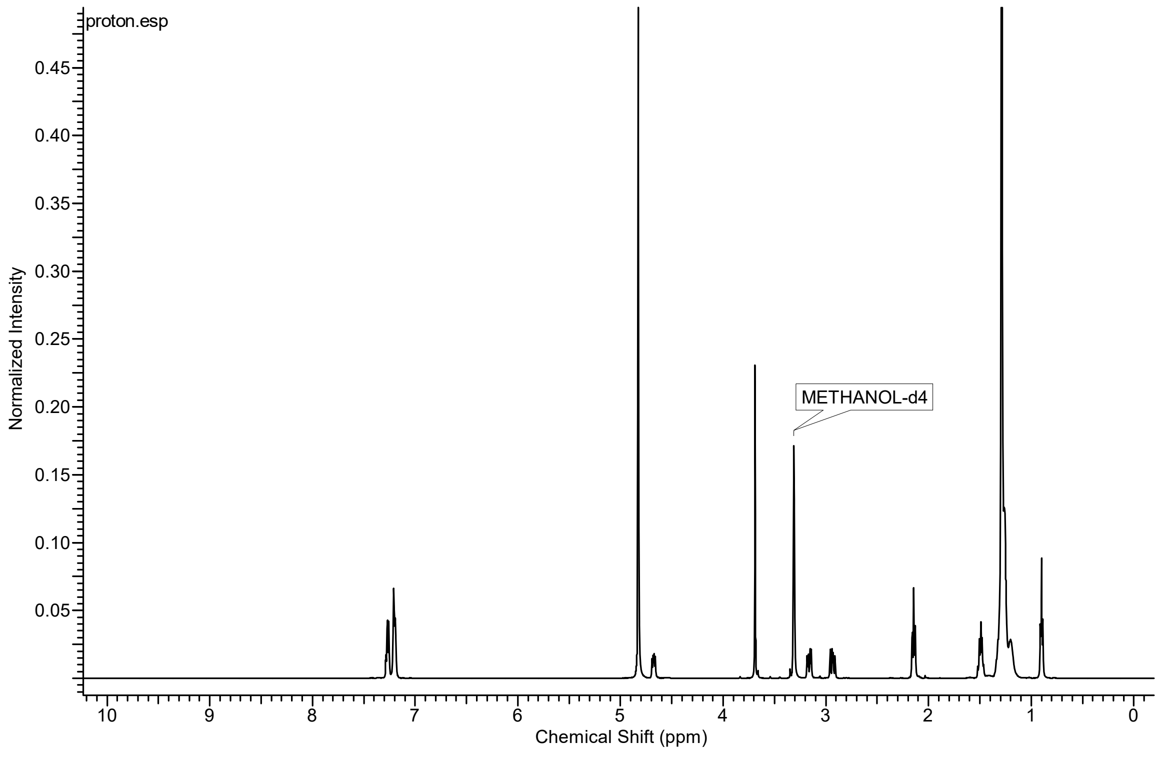

Figure S65. ^{13}C NMR spectrum for compound 17 in CD_3OD

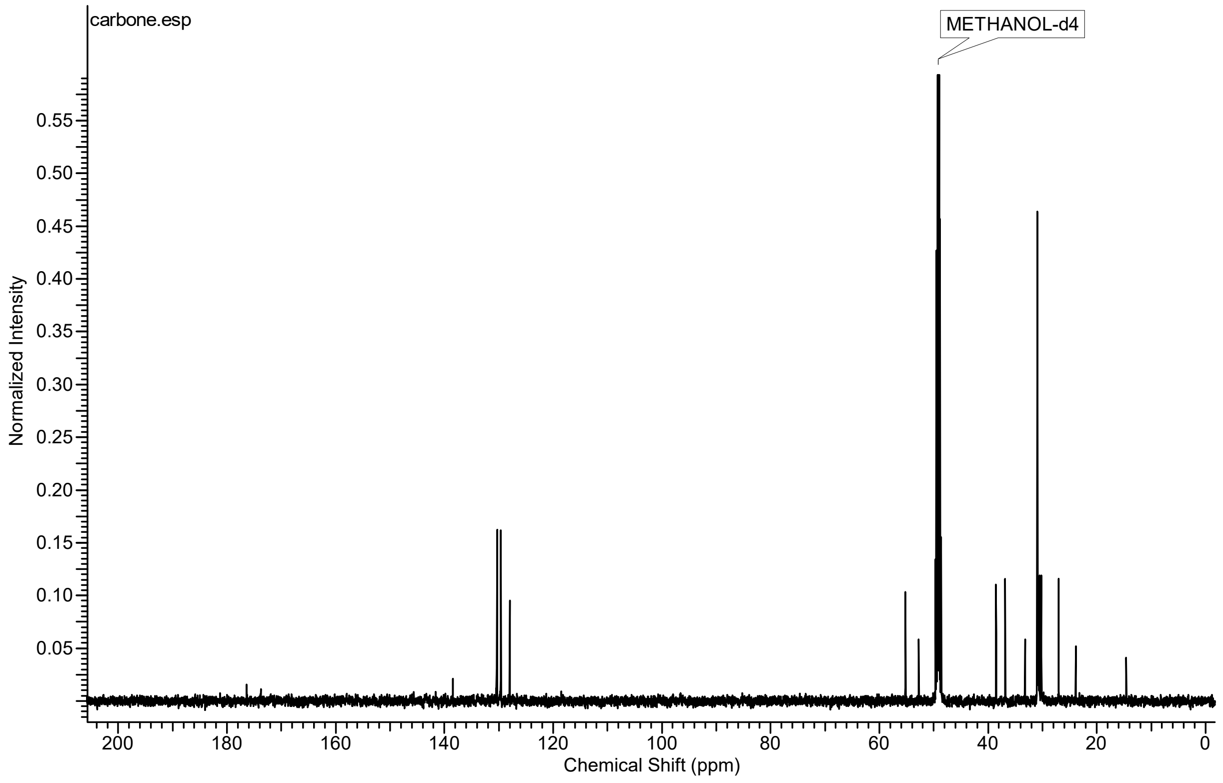

Figure S66. HRMS of compound 17 in MeOH

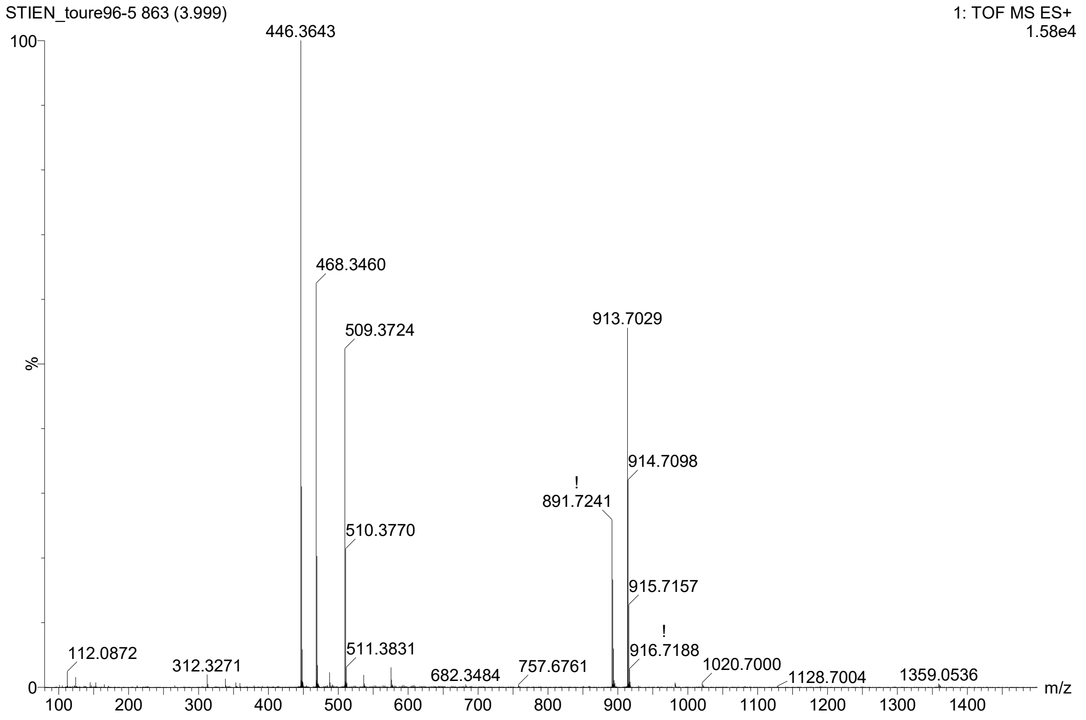

Figure S67. 1H NMR spectrum for compound 18 in CD3OD

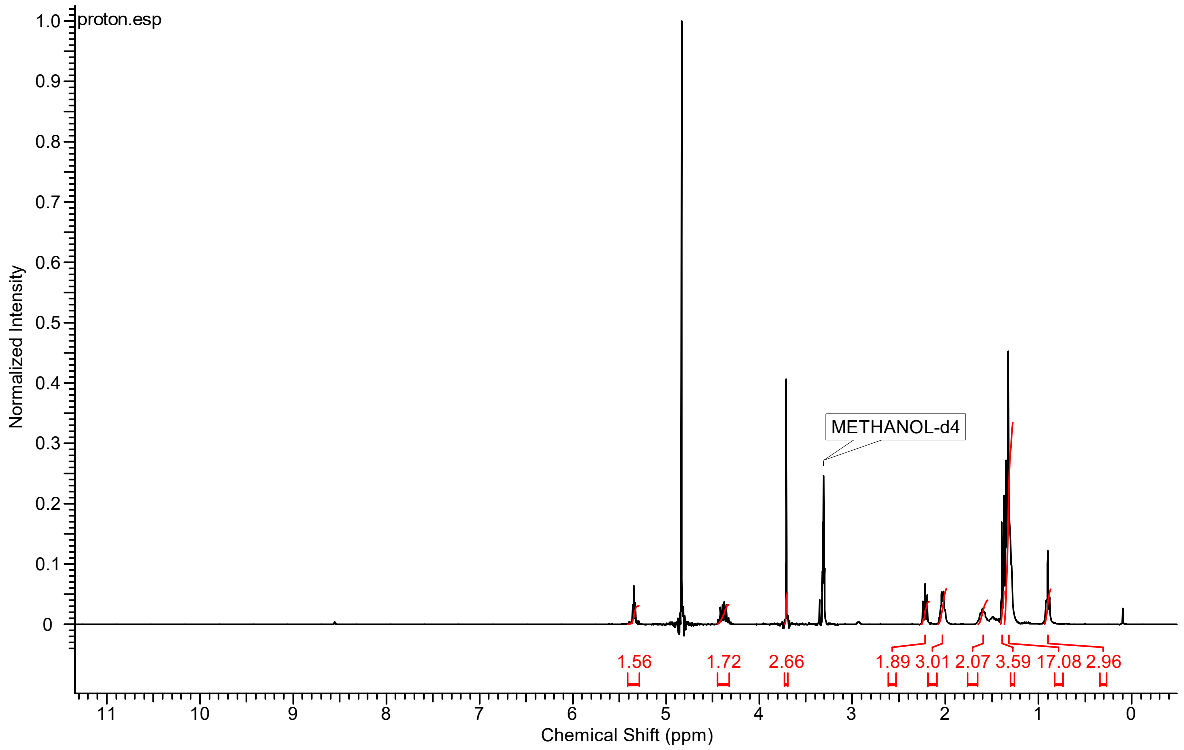

Figure S68. 13C NMR spectrum for compound 18 in CD3OD

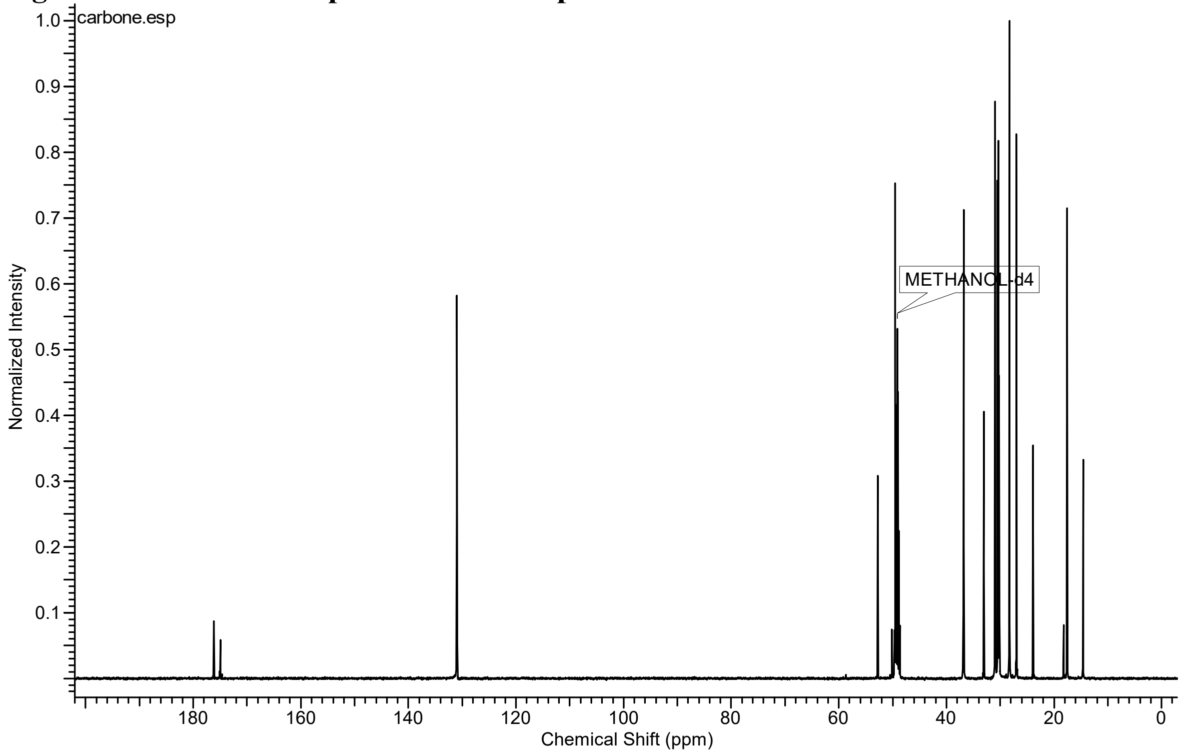

Figure S69. HRMS of compound 18 in MeOH

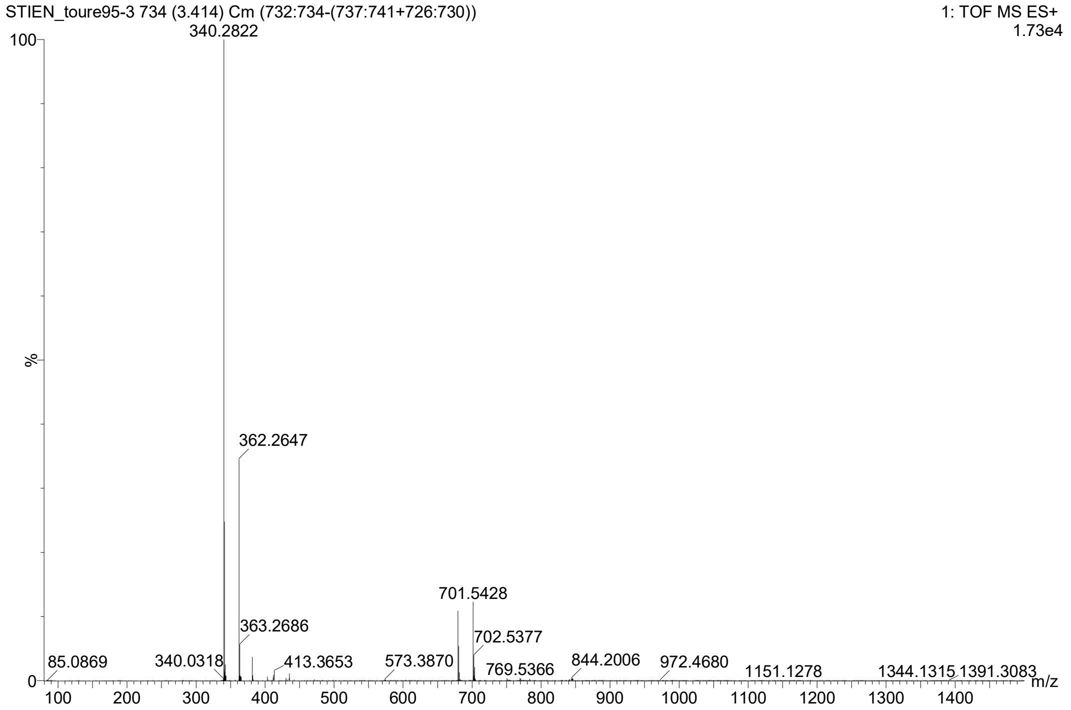

Figure S70. 1H NMR spectrum for compound 19 in CD3OD

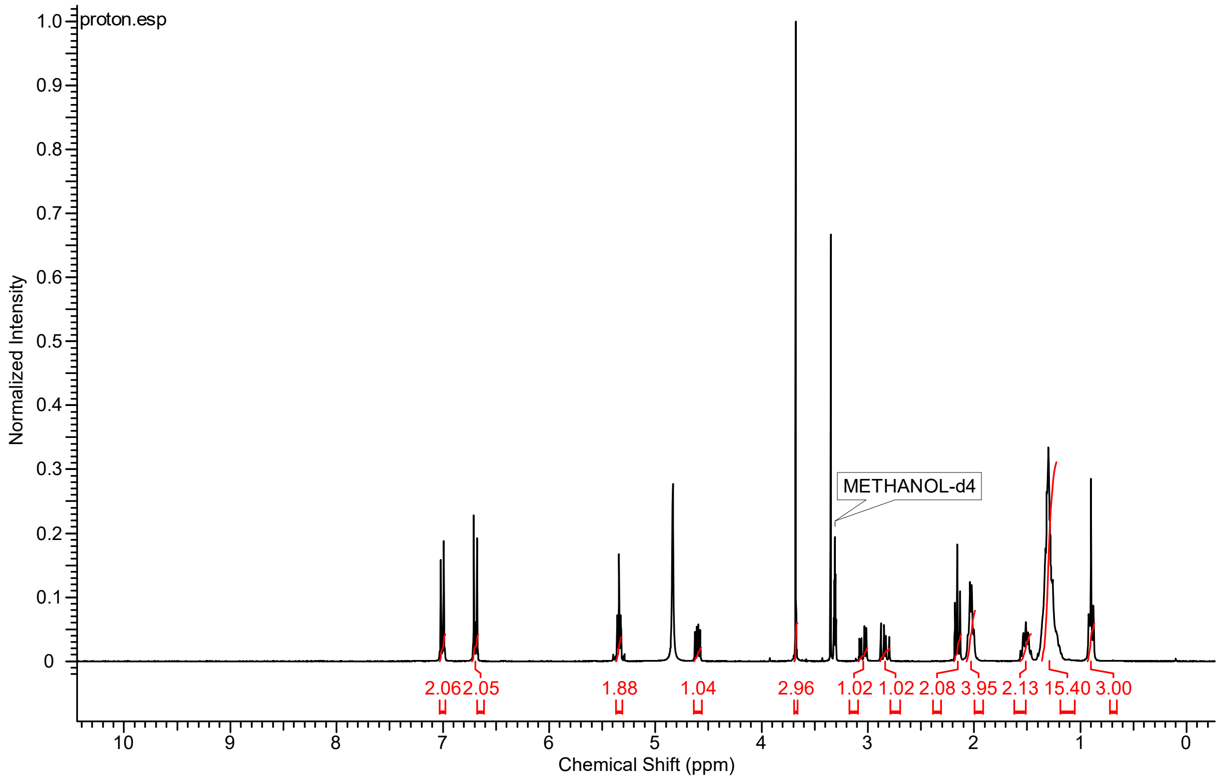

Figure S71. 13C NMR spectrum for compound 19 in CD3OD

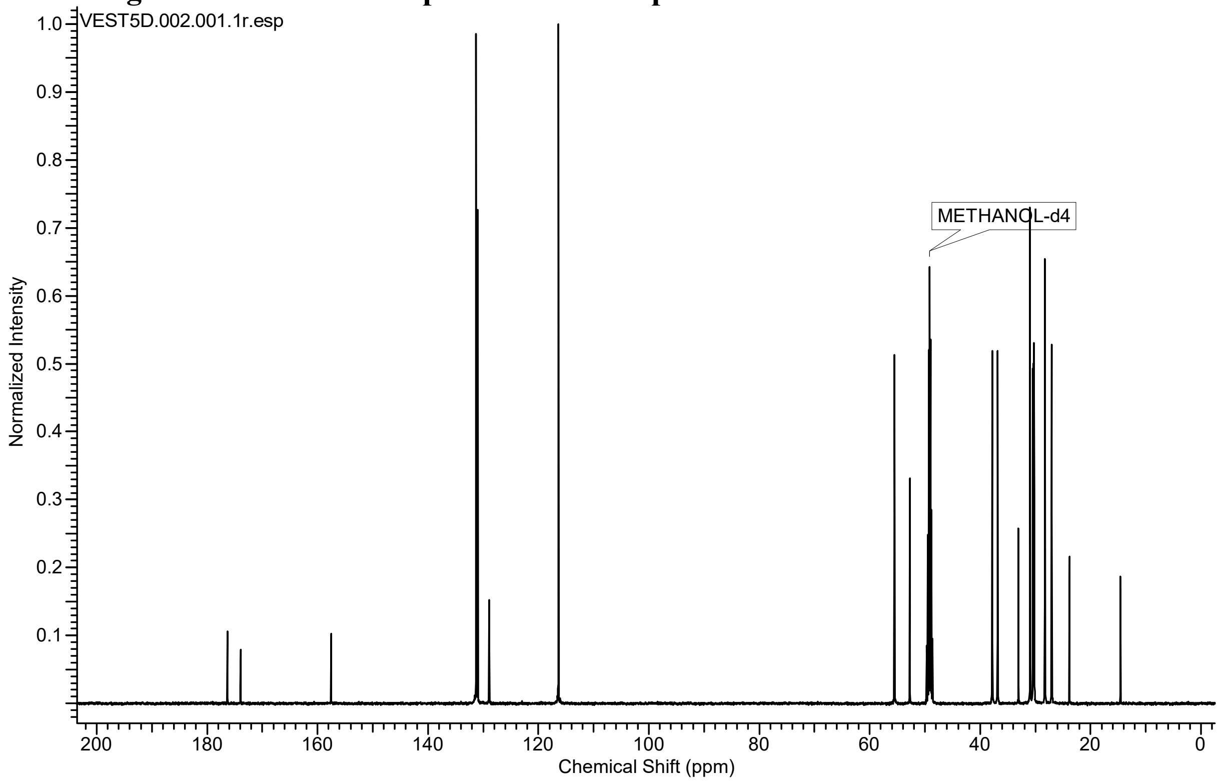

Figure S72. HRMS of compound 19 in MeOH

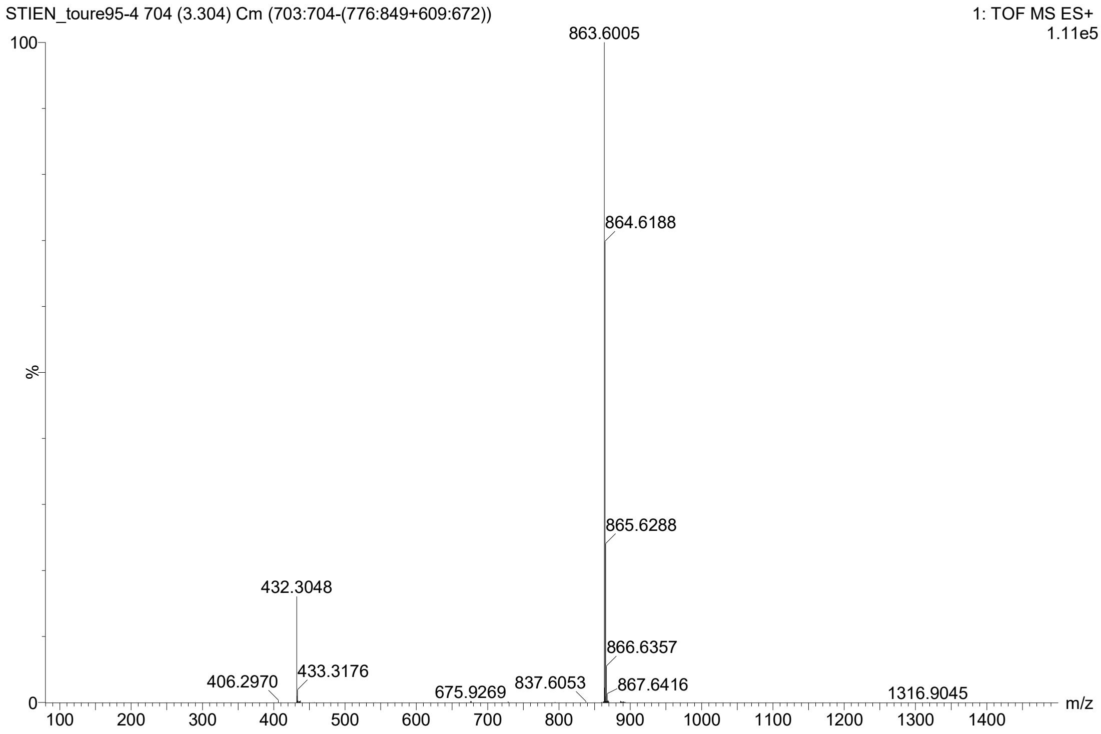

Figure S73. 1H NMR spectrum for compound 20 in CD3OD

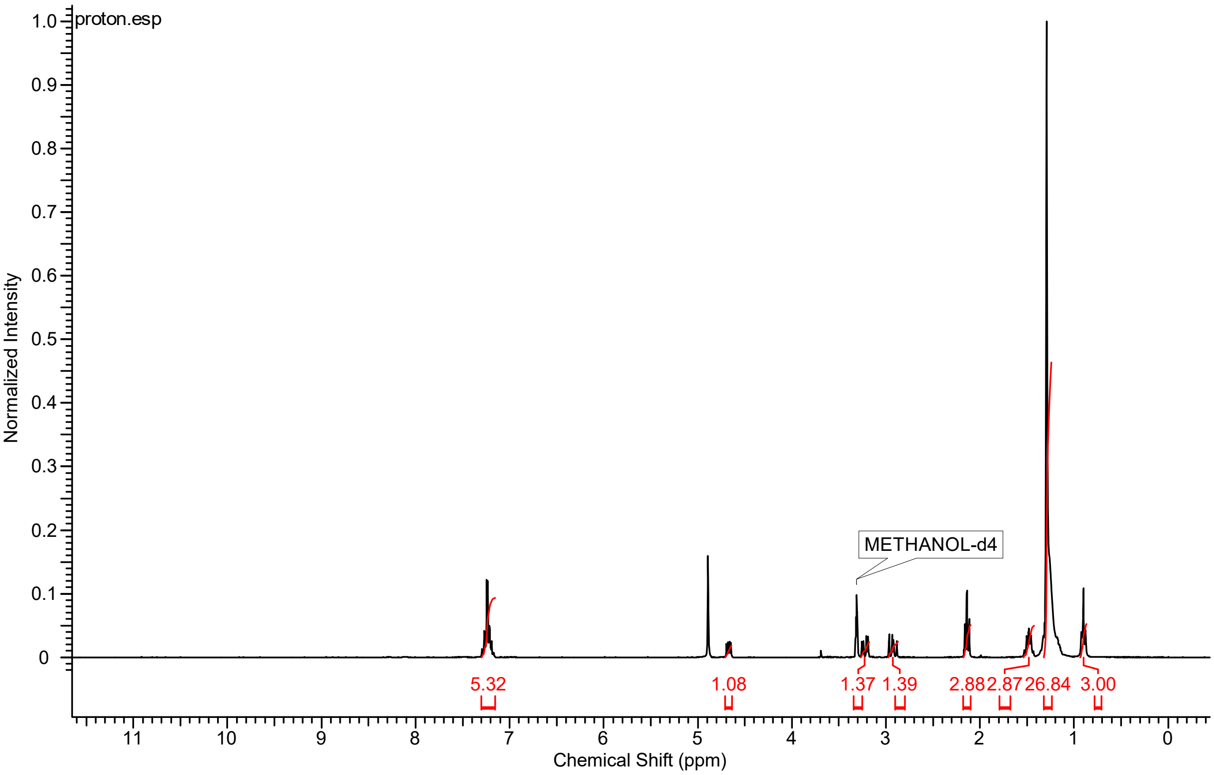

Figure S74. 13C NMR spectrum for compound 20 in CD3OD

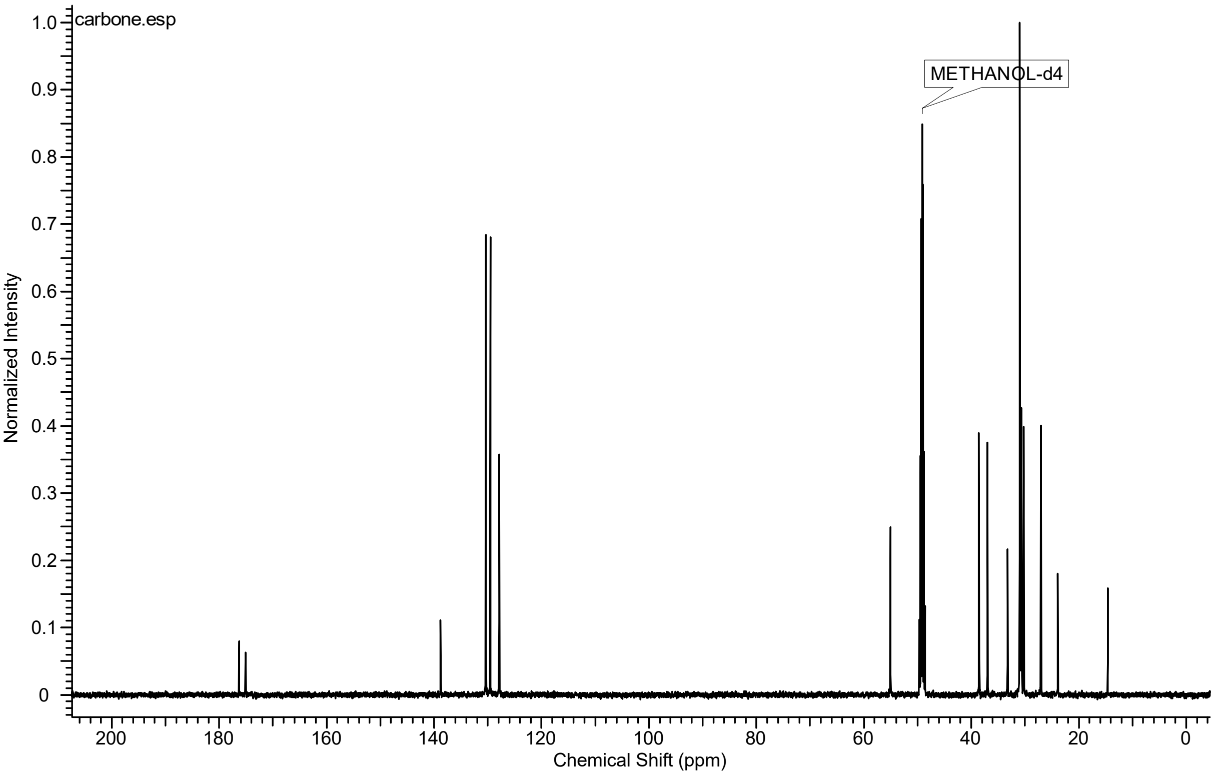

Figure S75. HRMS of compound 20 in MeOH

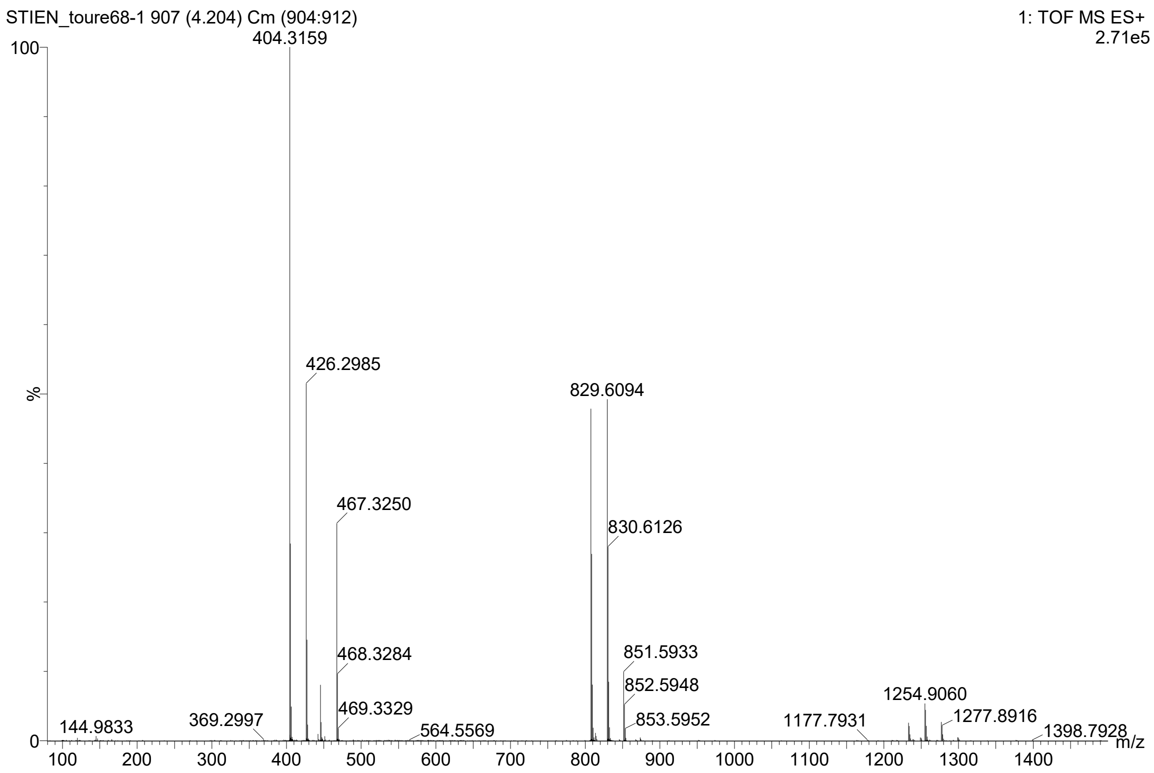

Figure S76. 1H NMR spectrum for compound 3 in CD3OD

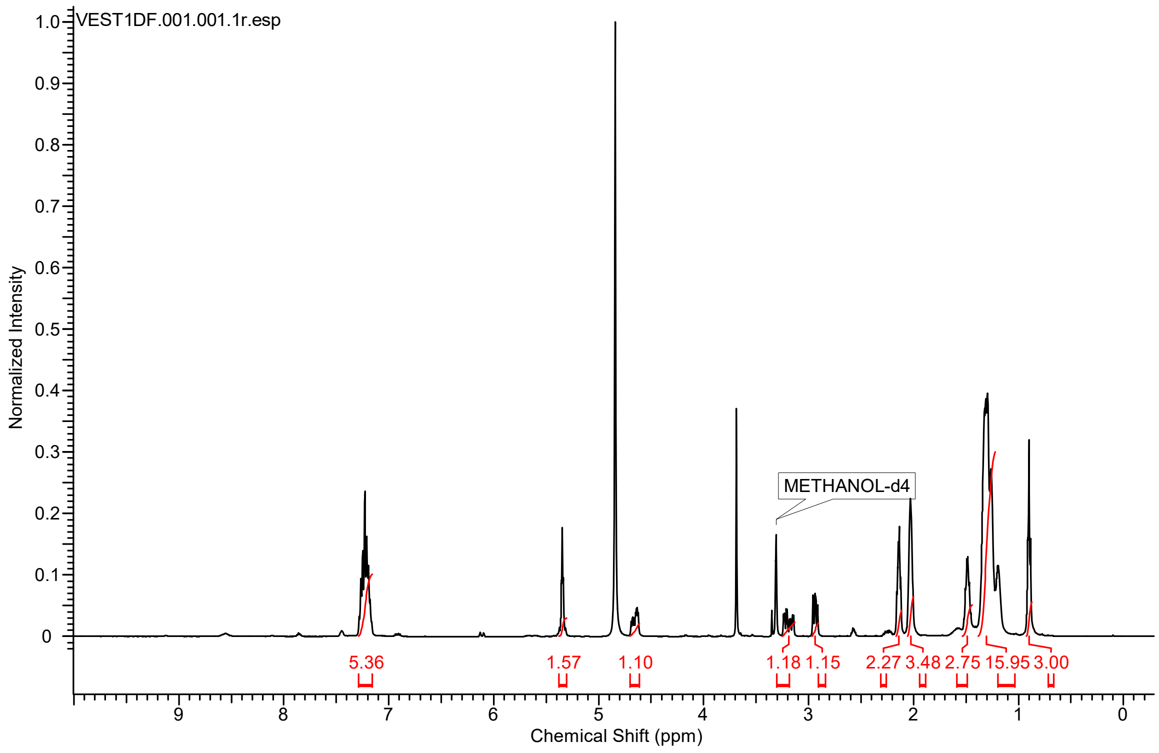

Figure S77. 13C NMR spectrum for compound 3 in CD3OD

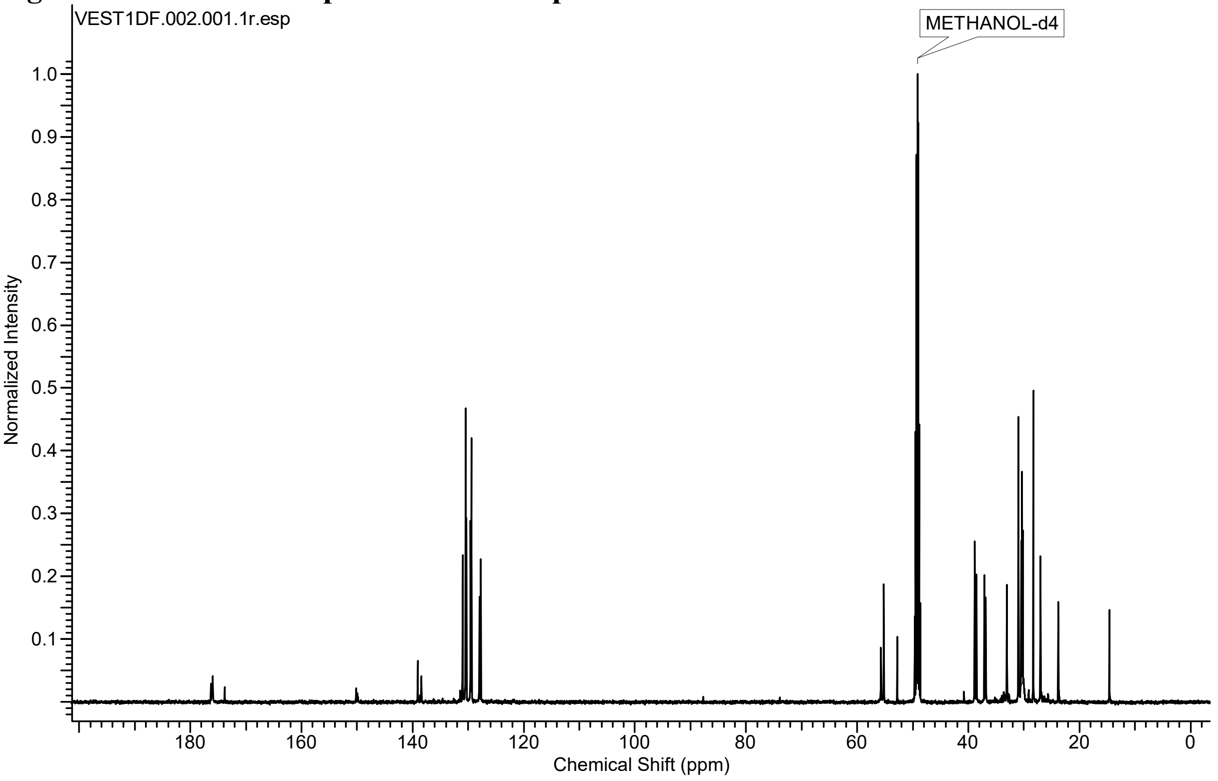

Figure S78. HRMS of compound 3 in MeOH

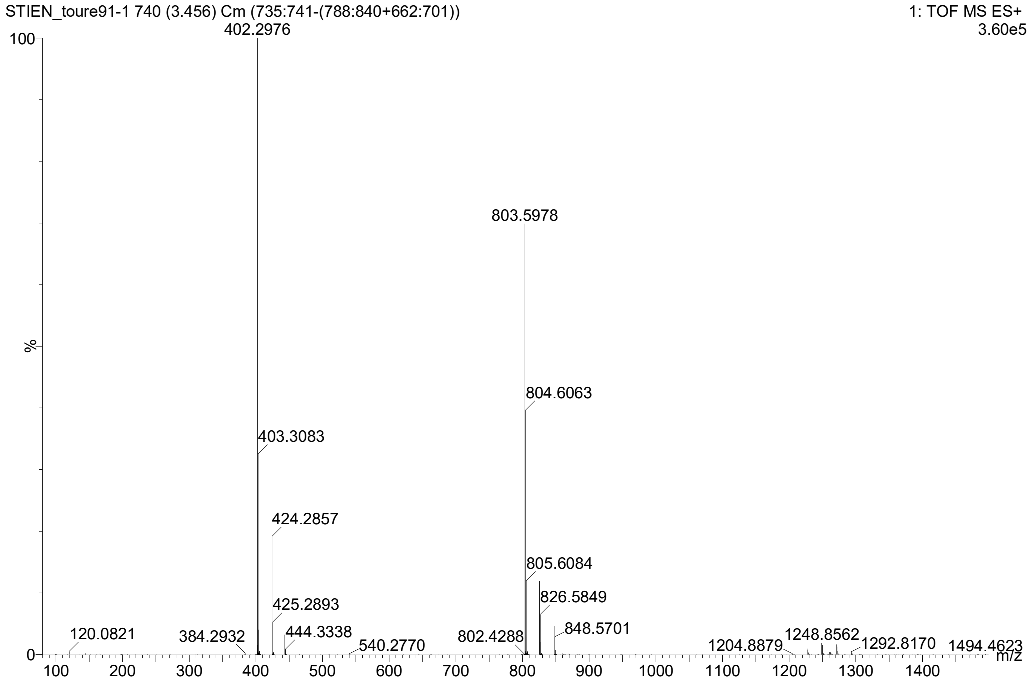

Figure S79. 1H NMR spectrum for compound 21 in CD_3OD

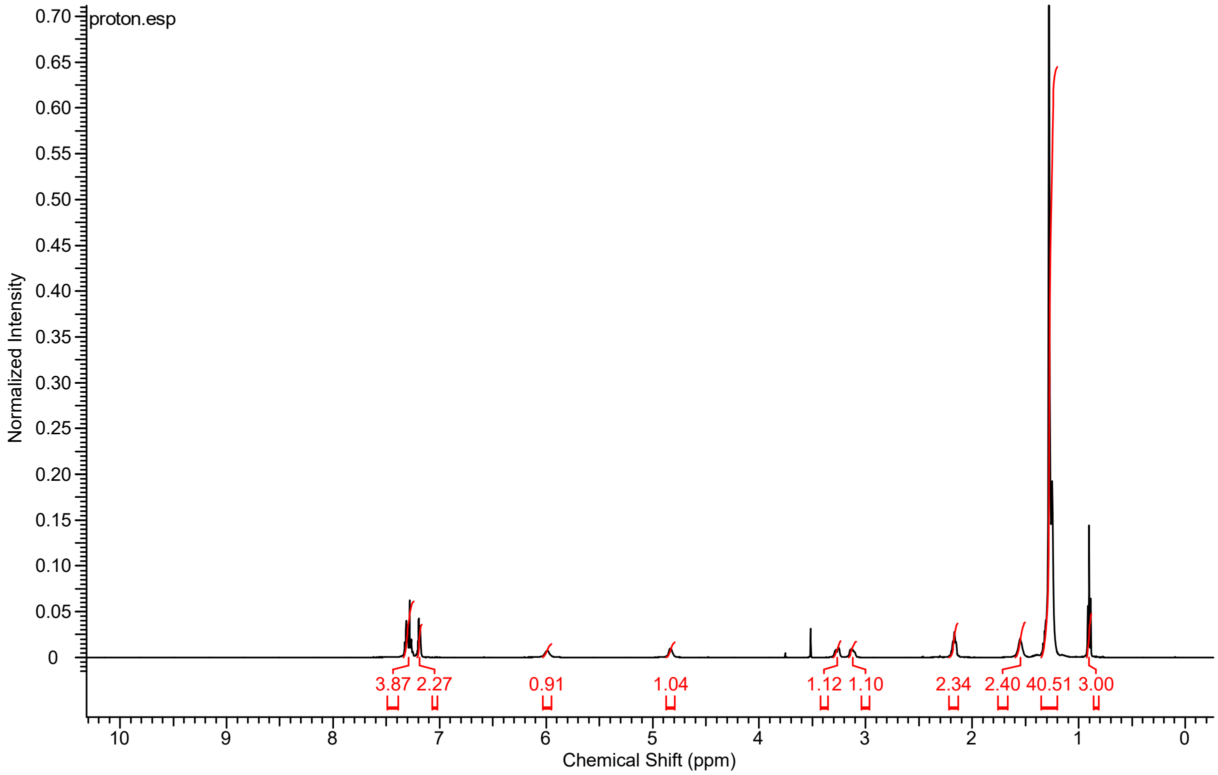

Figure S80. ^{13}C NMR spectrum for compound 21 in CD_3OD

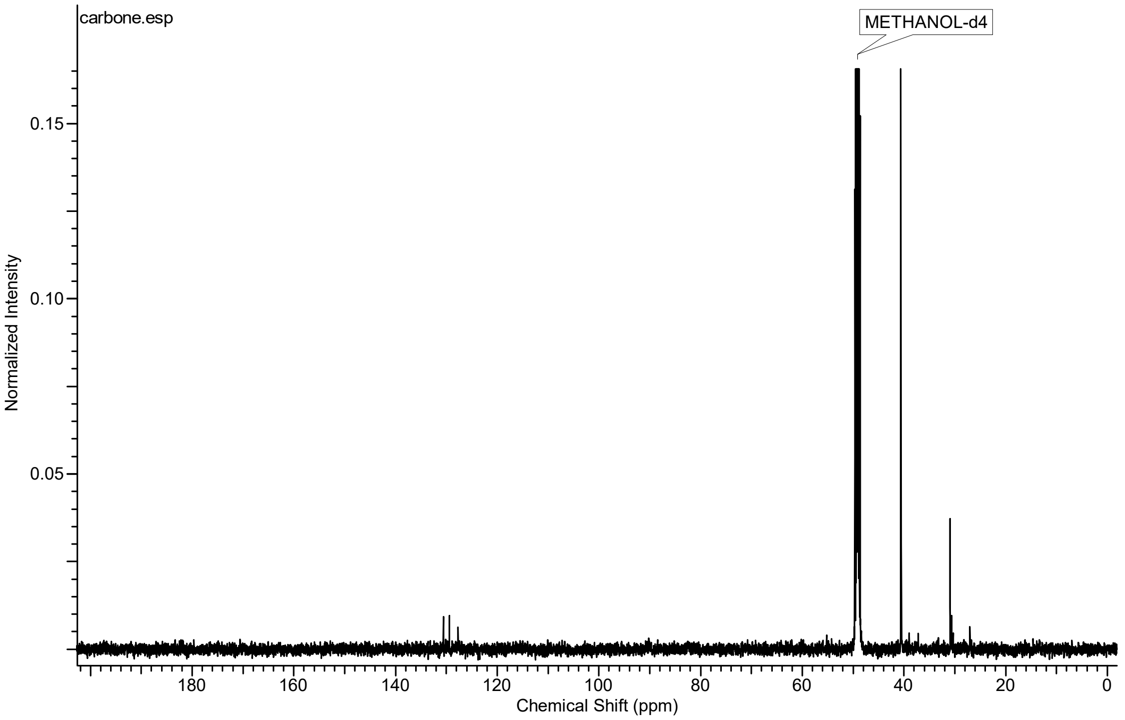

Figure S81. HRMS of compound 21 in MeOH

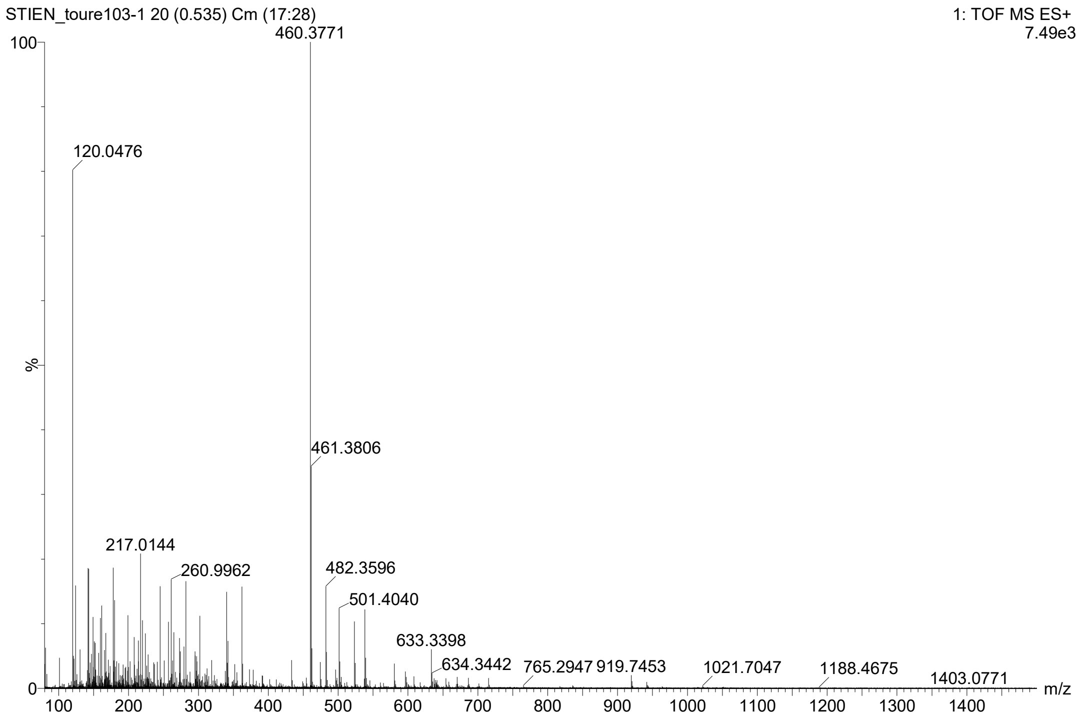

Figure S82. 1H NMR spectrum for compound 22 in CD3OD

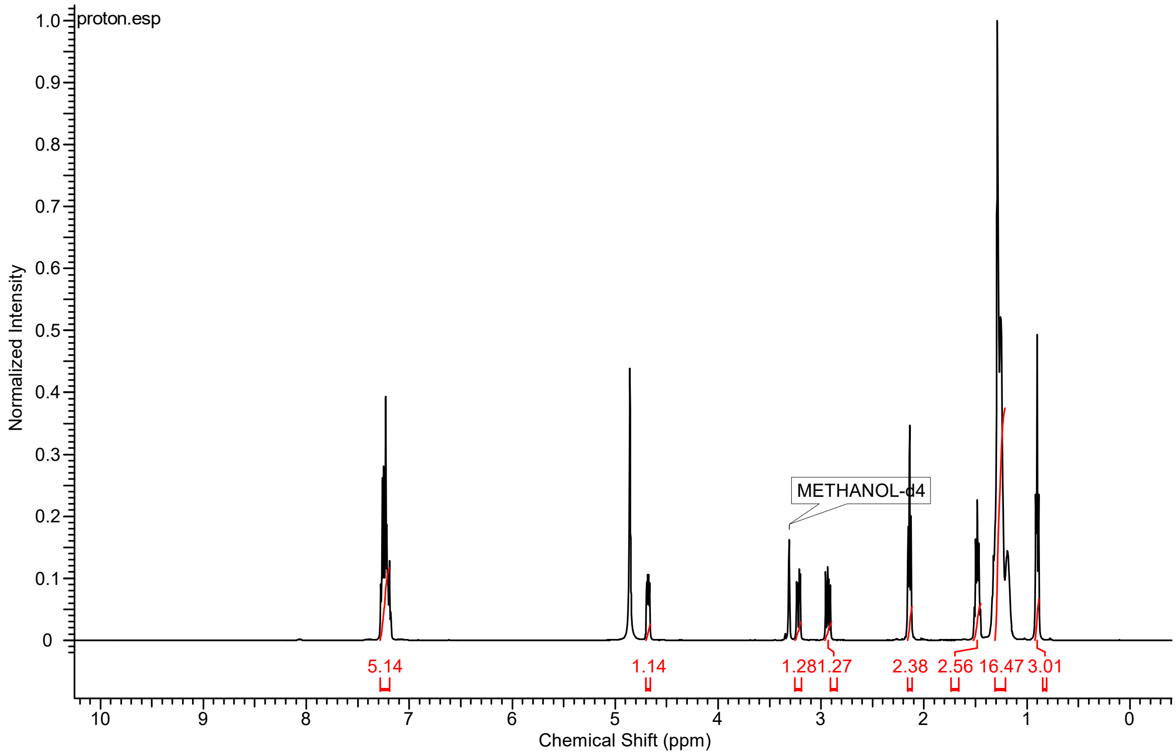

Figure S83. ^{13}C NMR spectrum for compound 22 in CD_3OD

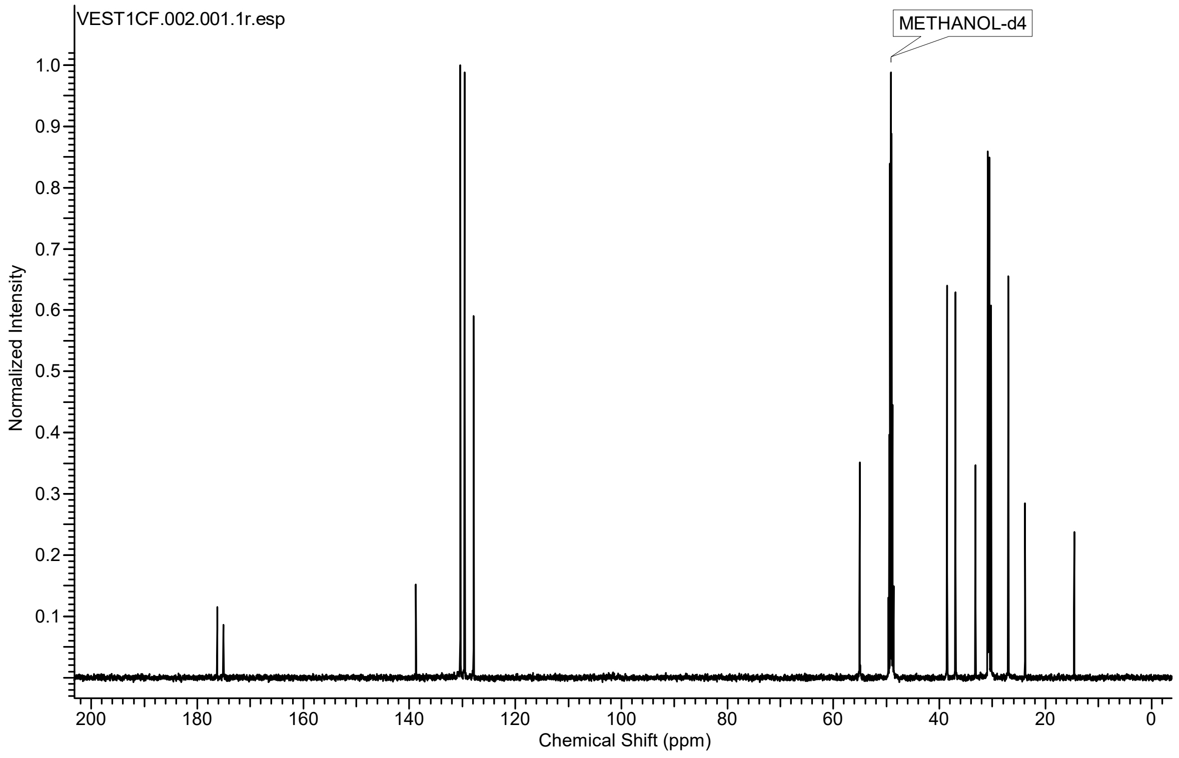

Figure S84. HRMS of compound 22 in MeOH

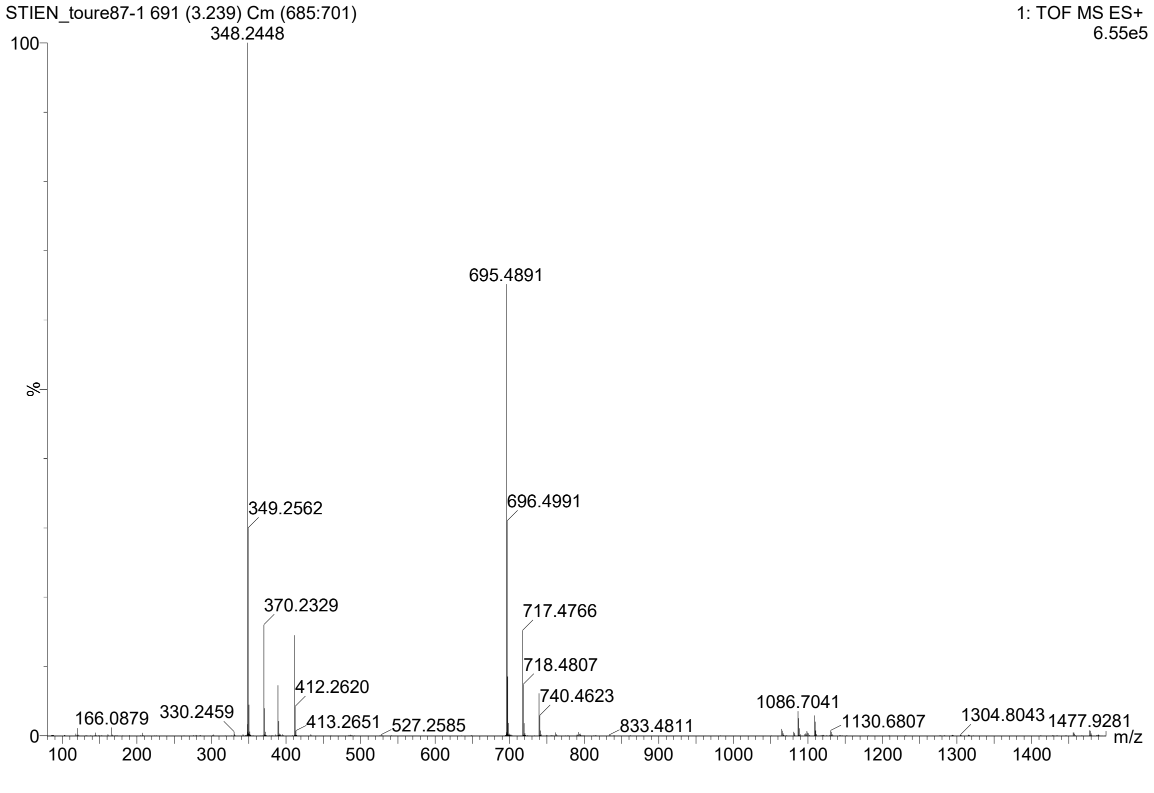

Figure S85. 1H NMR spectrum for compound 23 in CD3OD

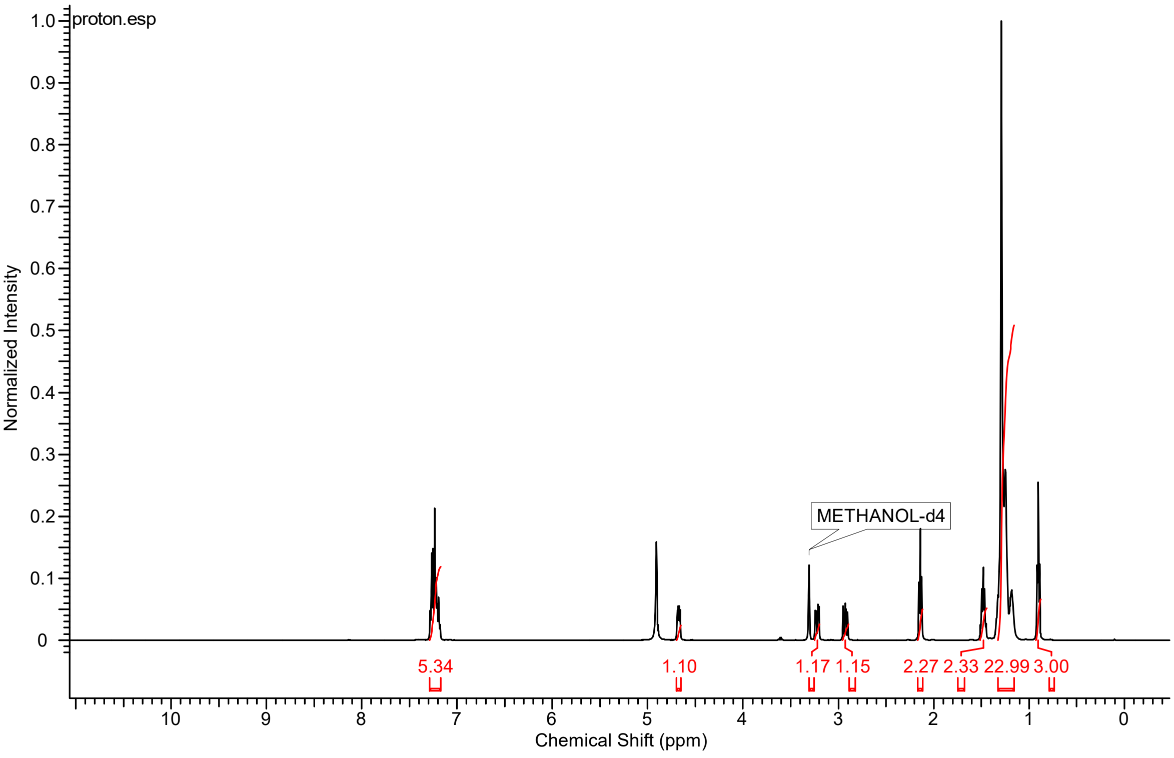

Figure S86. ^{13}C NMR spectrum for compound 23 in CD3OD

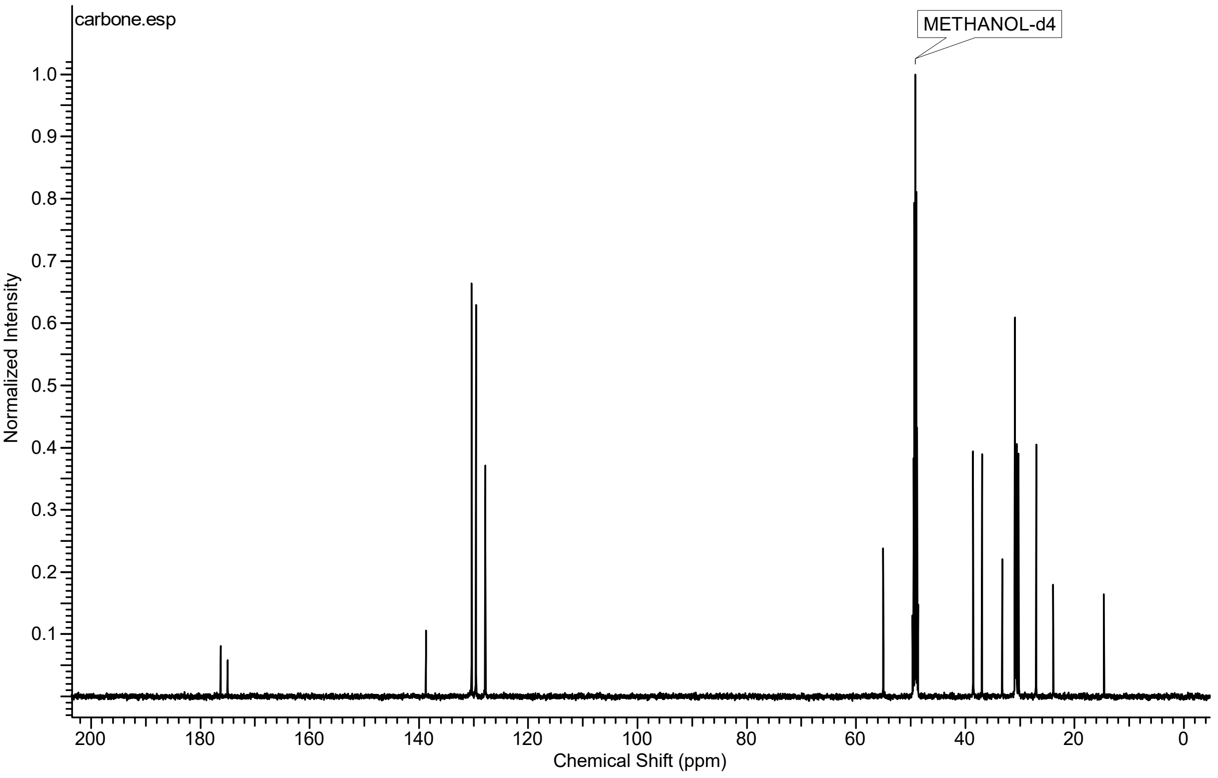

Figure S87. HRMS of compound 23 in MeOH

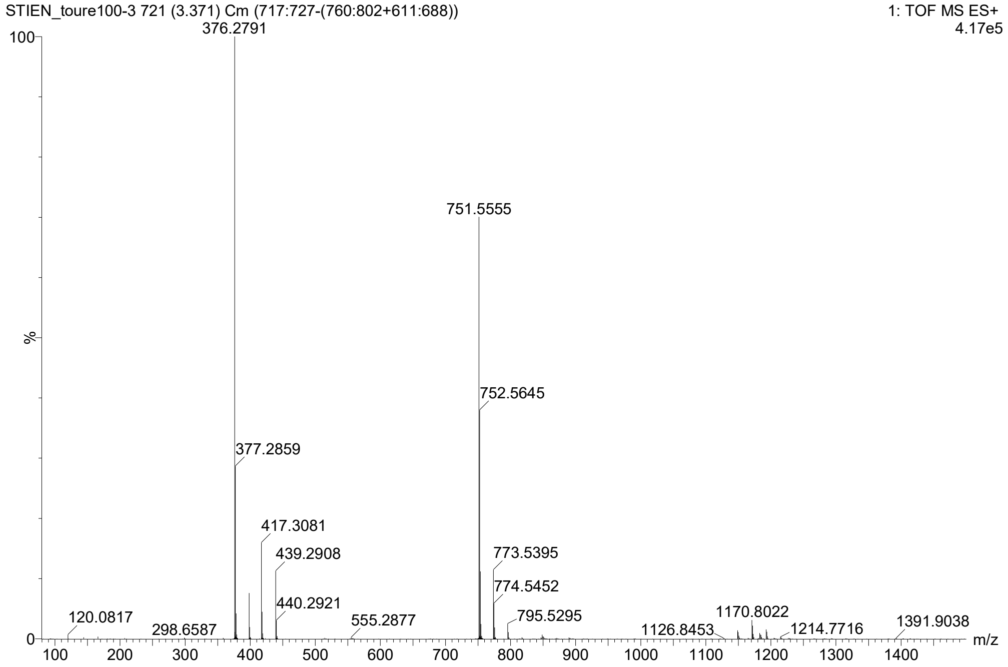

Figure S88. 1H NMR spectrum for compound 24 in CD_3OD

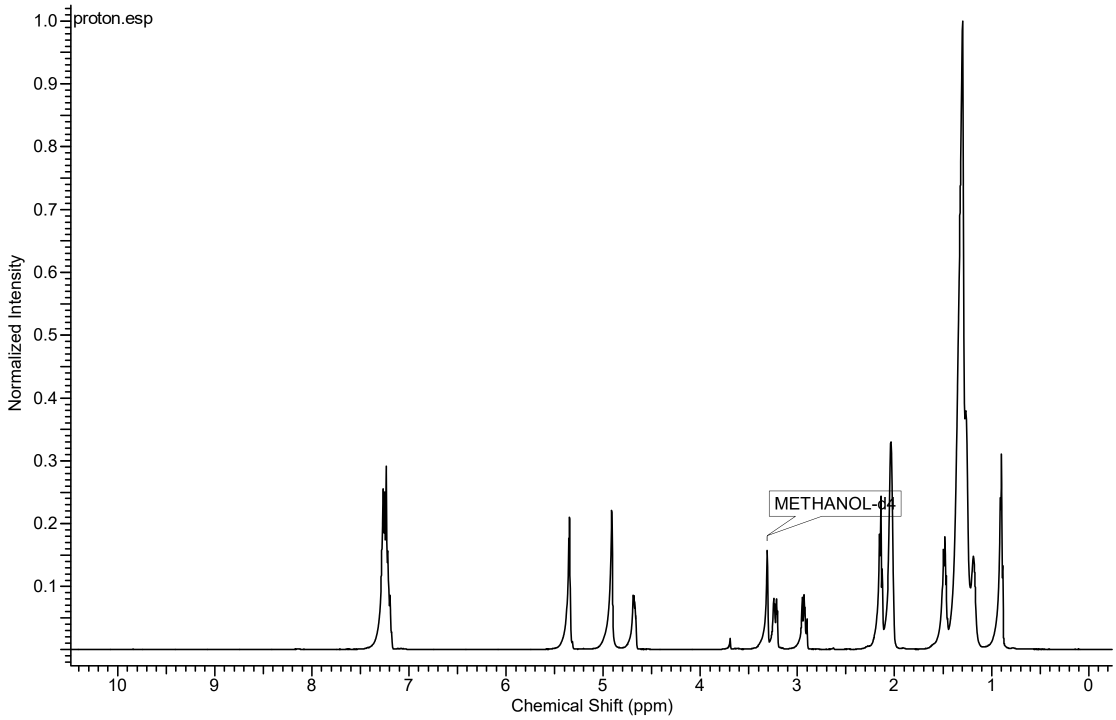

Figure S89. ^{13}C NMR spectrum for compound 24 in CD_3OD

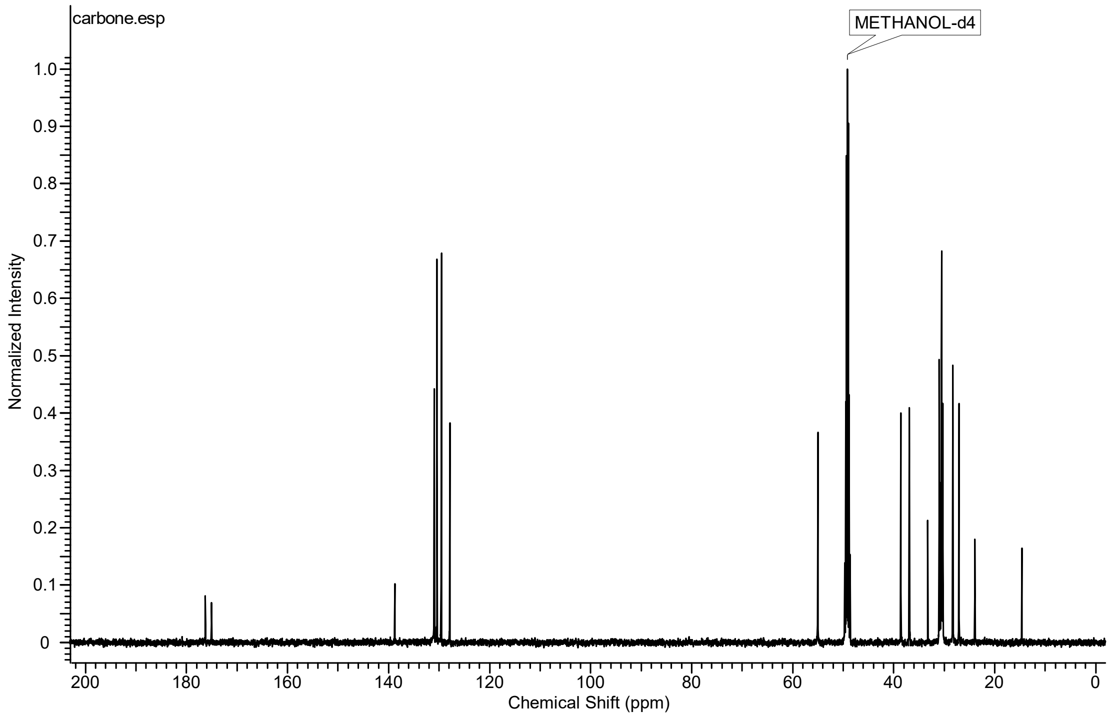

Figure S90. HRMS of compound 24 in MeOH

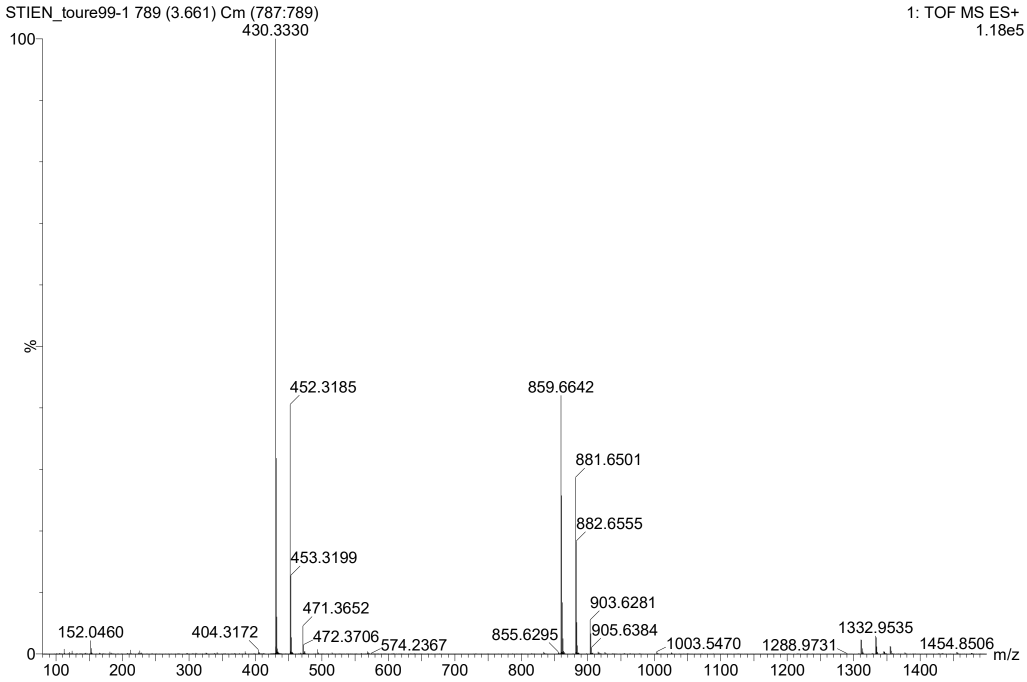

Figure S91. 1H NMR spectrum for compound 25 in CD3OD

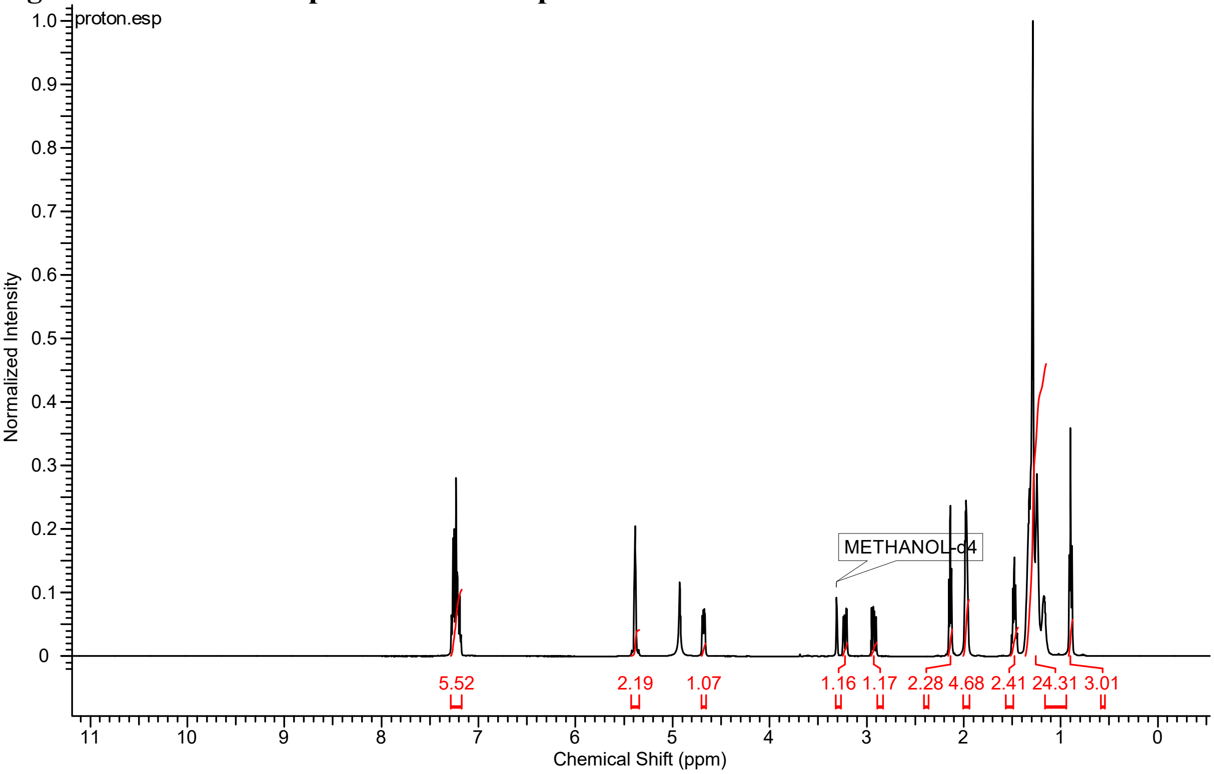

Figure S92. 13C NMR spectrum for compound 25 in CD3OD

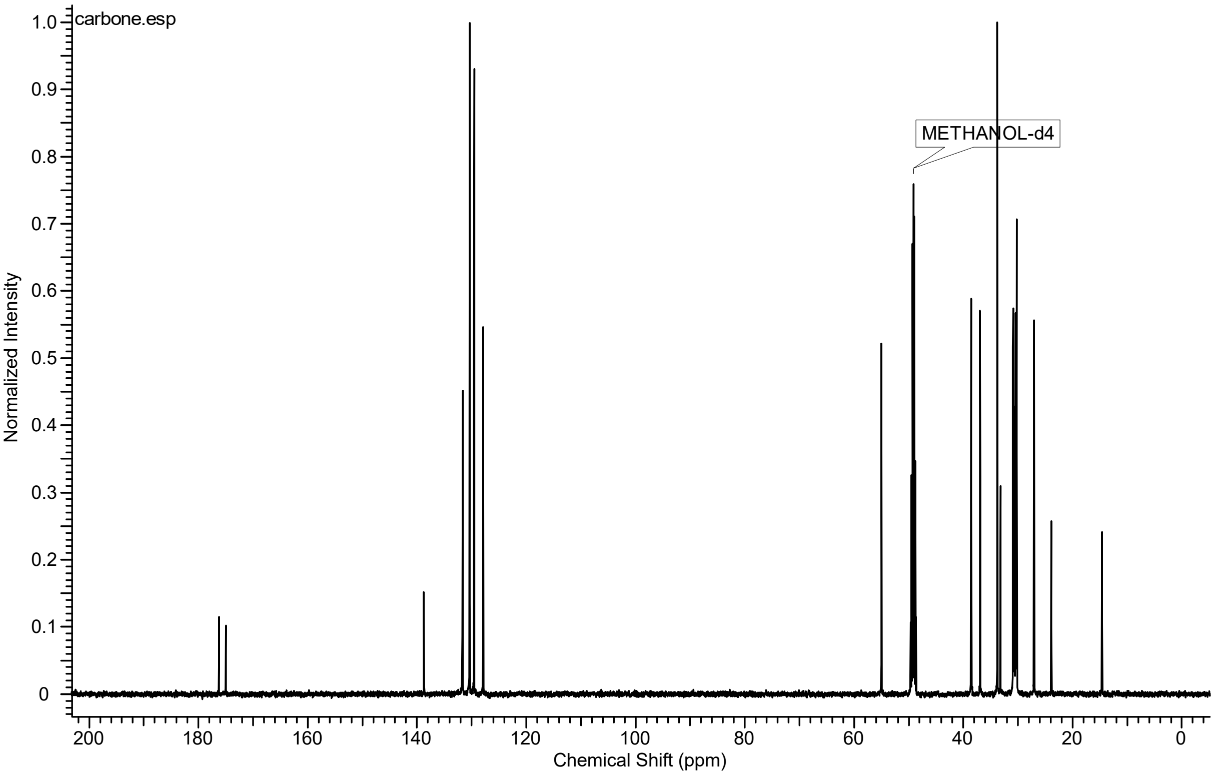

Figure S93. HRMS of compound 25 in MeOH

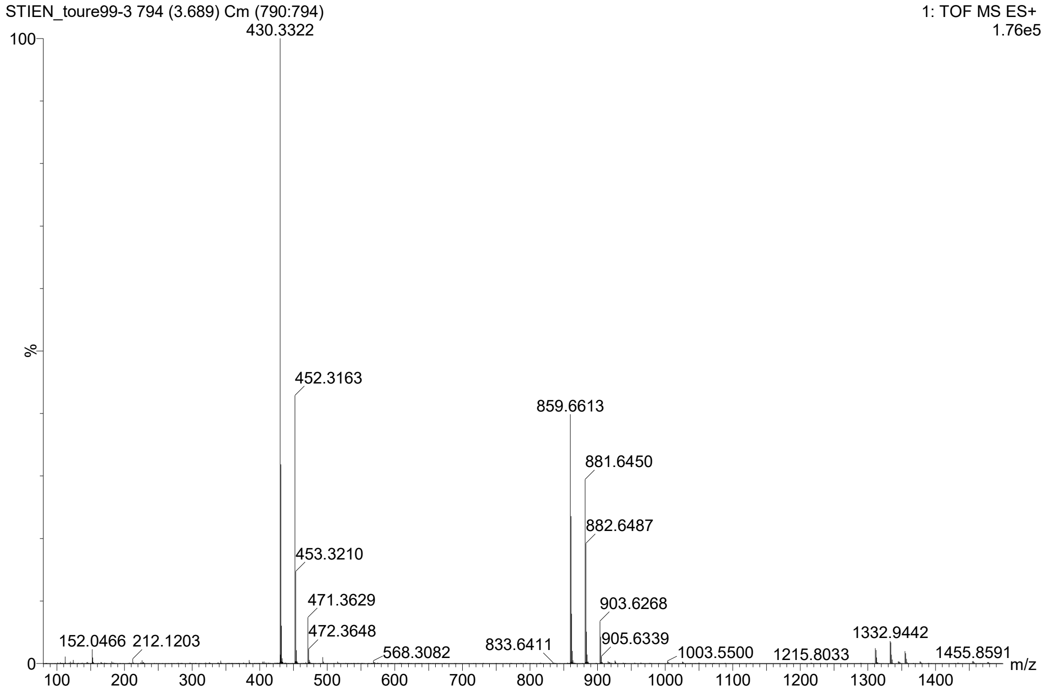

Figure S94. 1H NMR spectrum for compound 26 in CD3OD

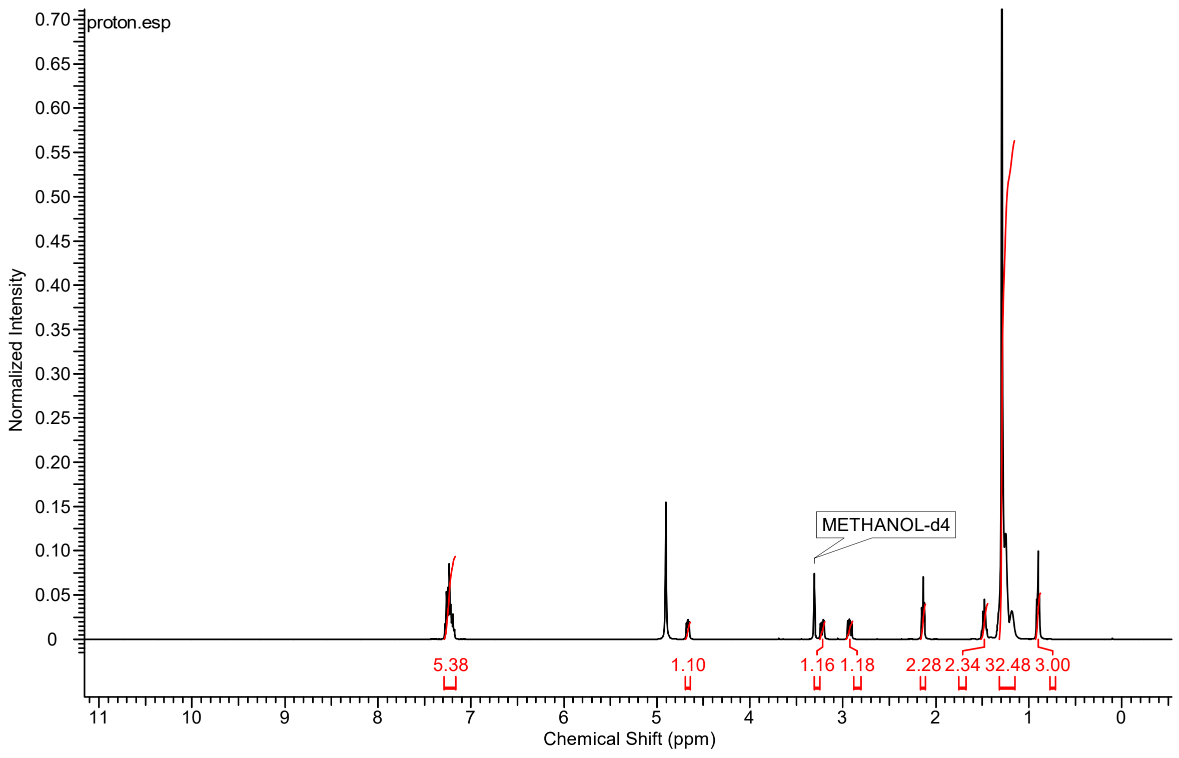

Figure S95. ^{13}C NMR spectrum for compound 26 in CD_3OD

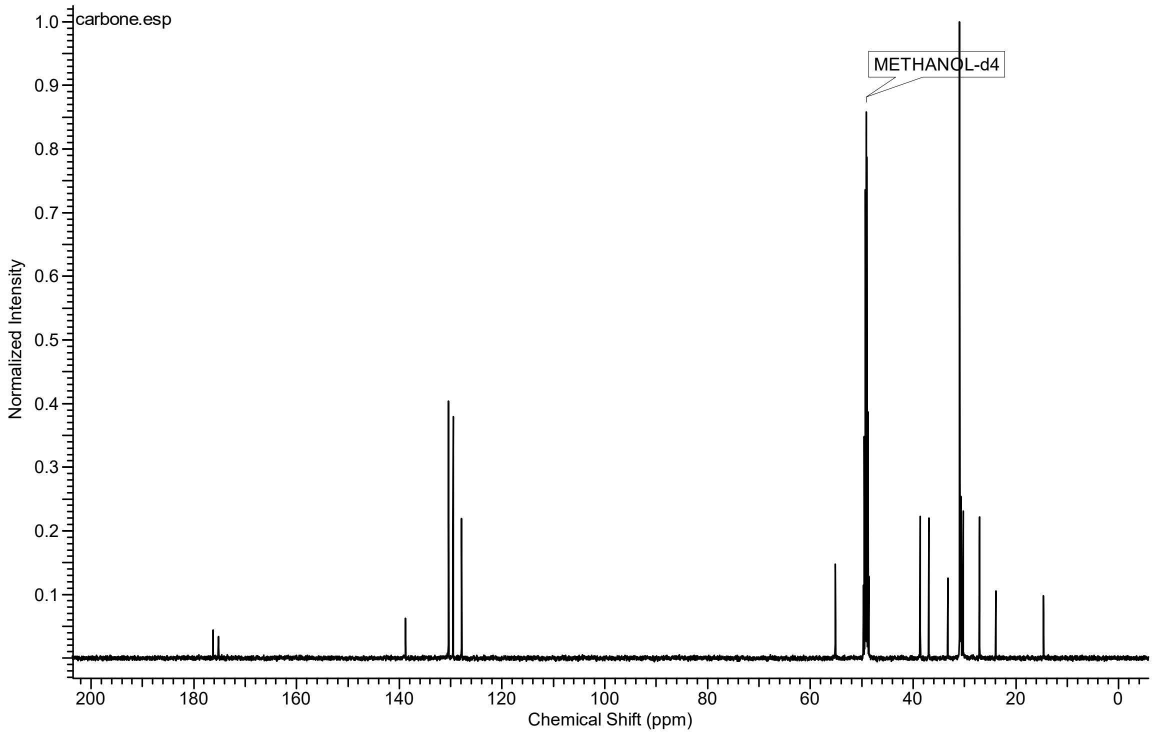

Figure S96. HRMS of compound 26 in MeOH

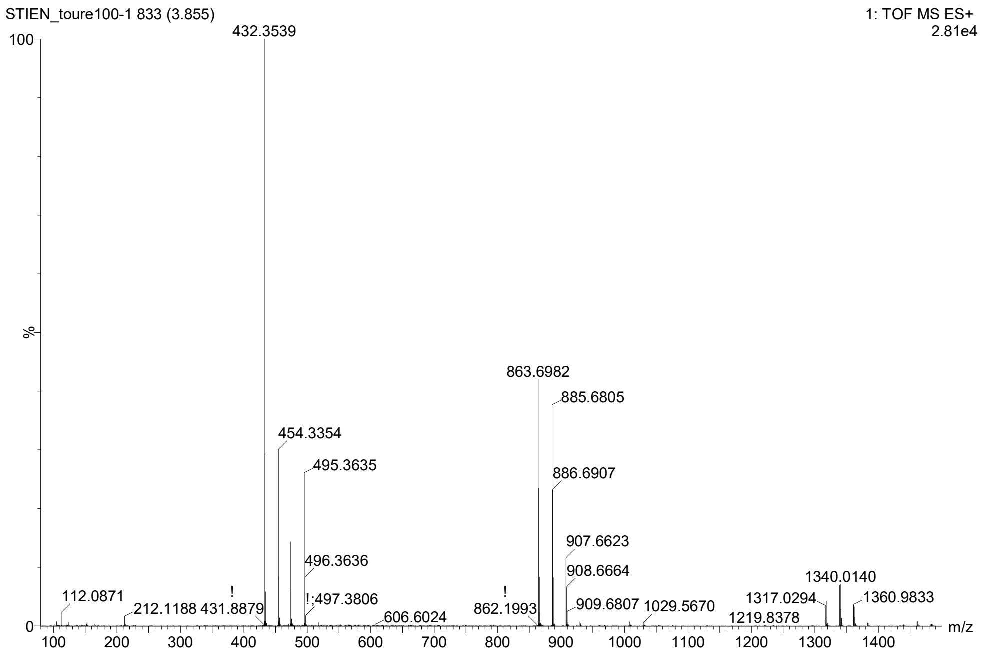

Figure S97. 1H NMR spectrum for compound 27 in CD3OD

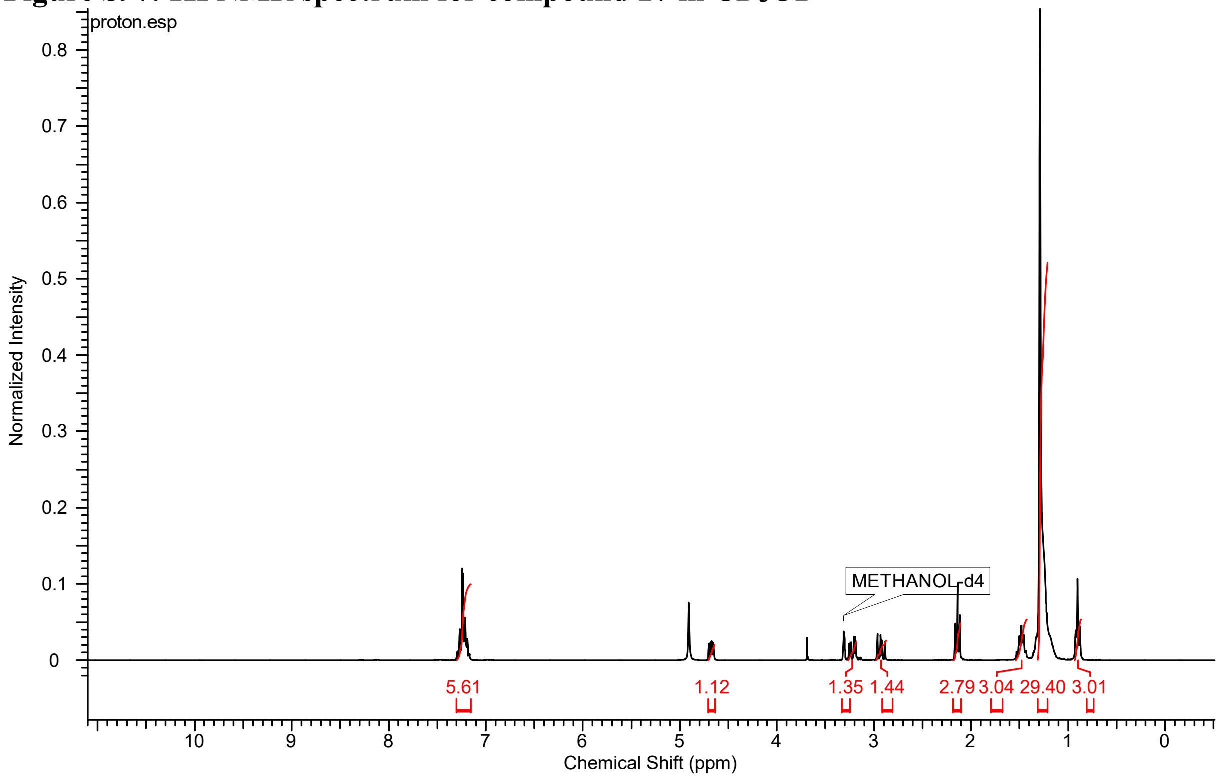

Figure S98. 13C NMR spectrum for compound 27 in CD3OD

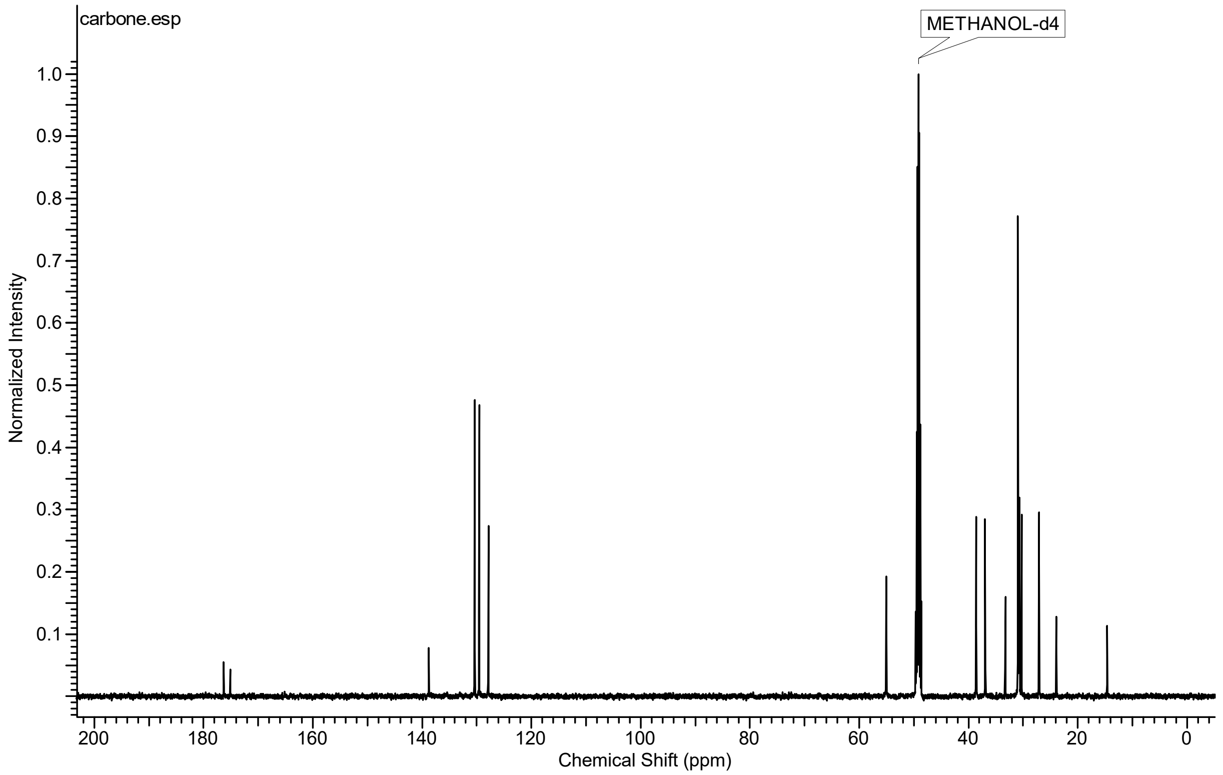

Figure S99. HRMS of compound 27 in MeOH

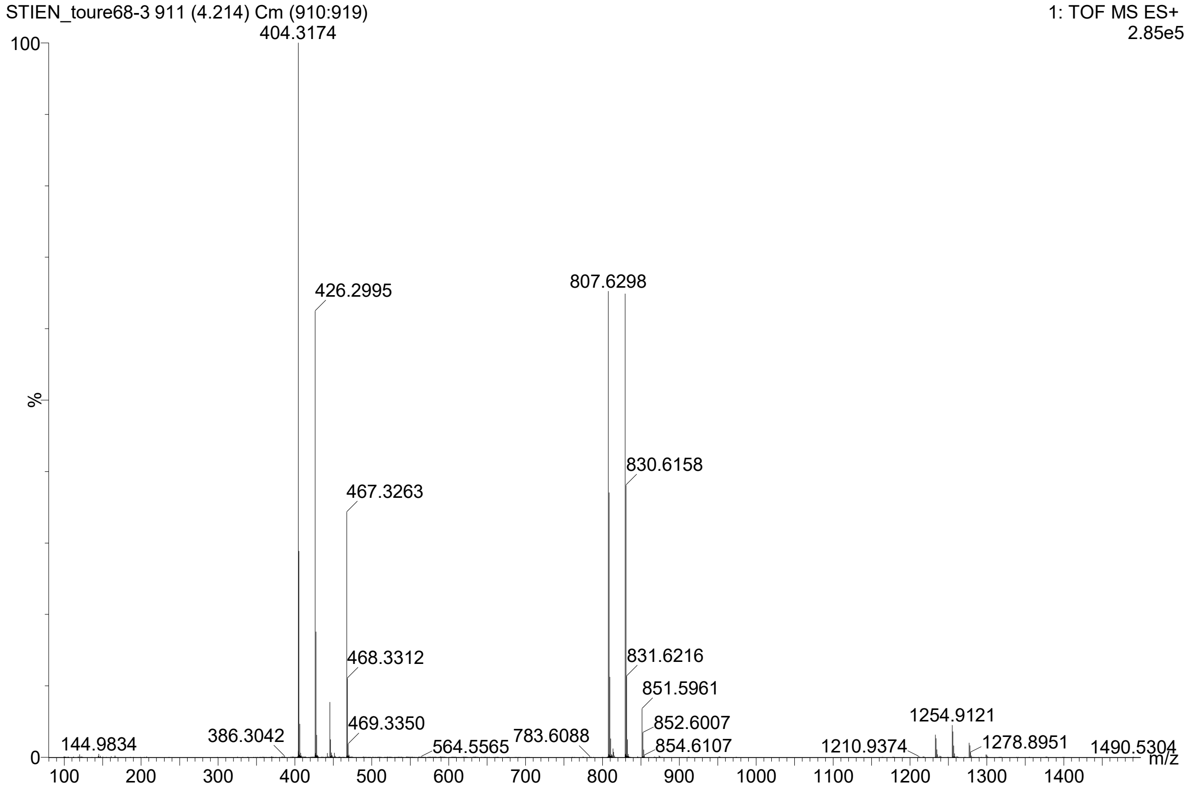

Figure S100. 1H NMR spectrum for compound *ent*-3 in CD3OD

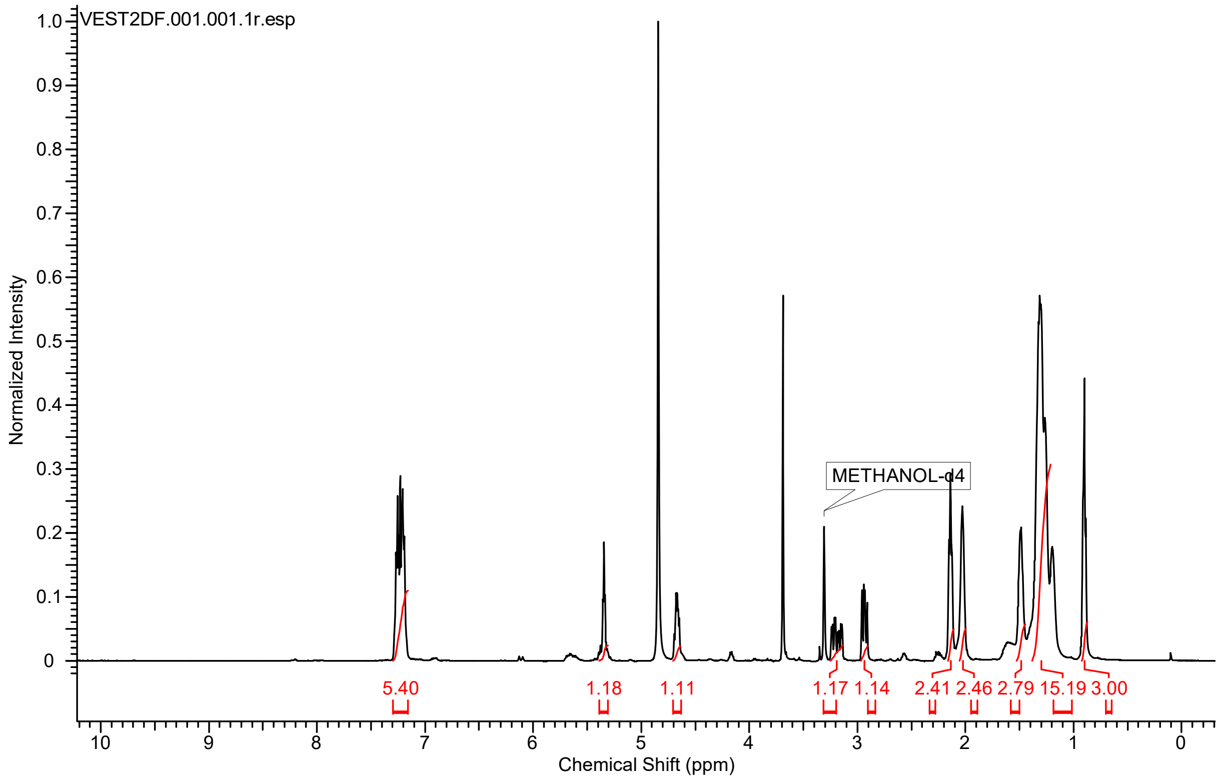

Figure S101. 13C NMR spectrum for compound *ent*-3 in CD_3OD

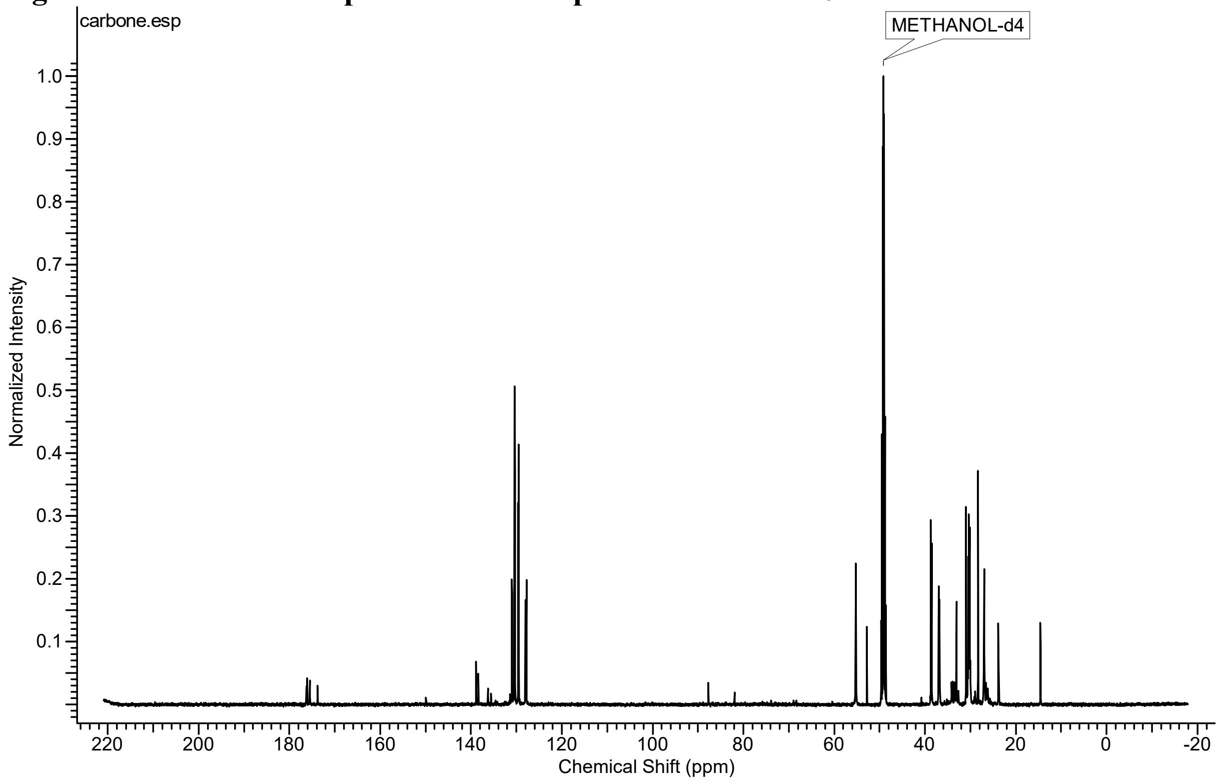

Figure S102. HRMS of compound *ent*-3 in MeOH

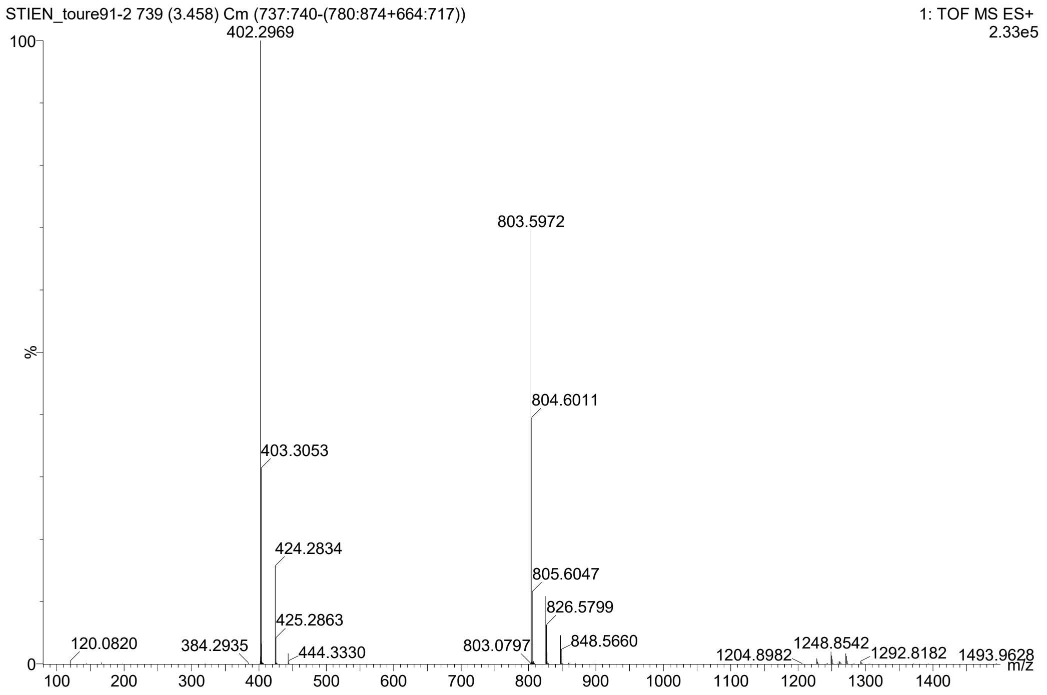

Figure S103. 1H NMR spectrum for compound 28 in CD_3OD

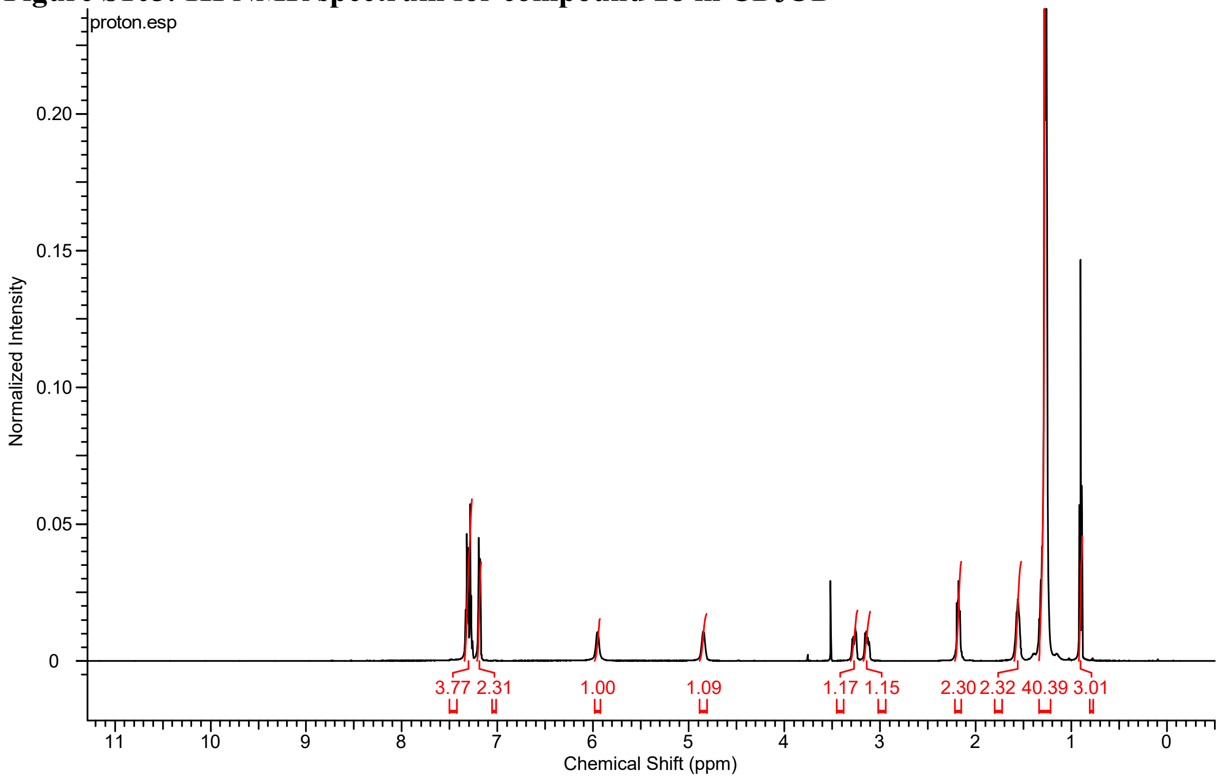

Figure S104. ^{13}C NMR spectrum for compound 28 in CD_3OD

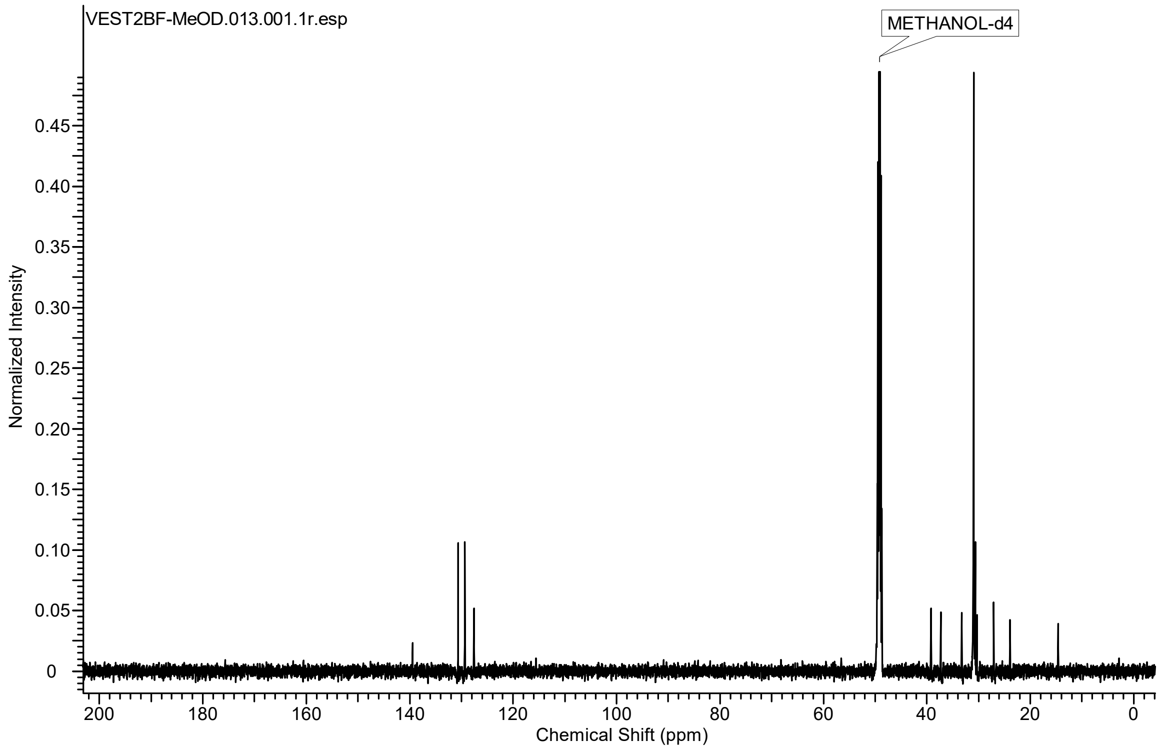

Figure S105. HRMS of compound 28 in MeOH

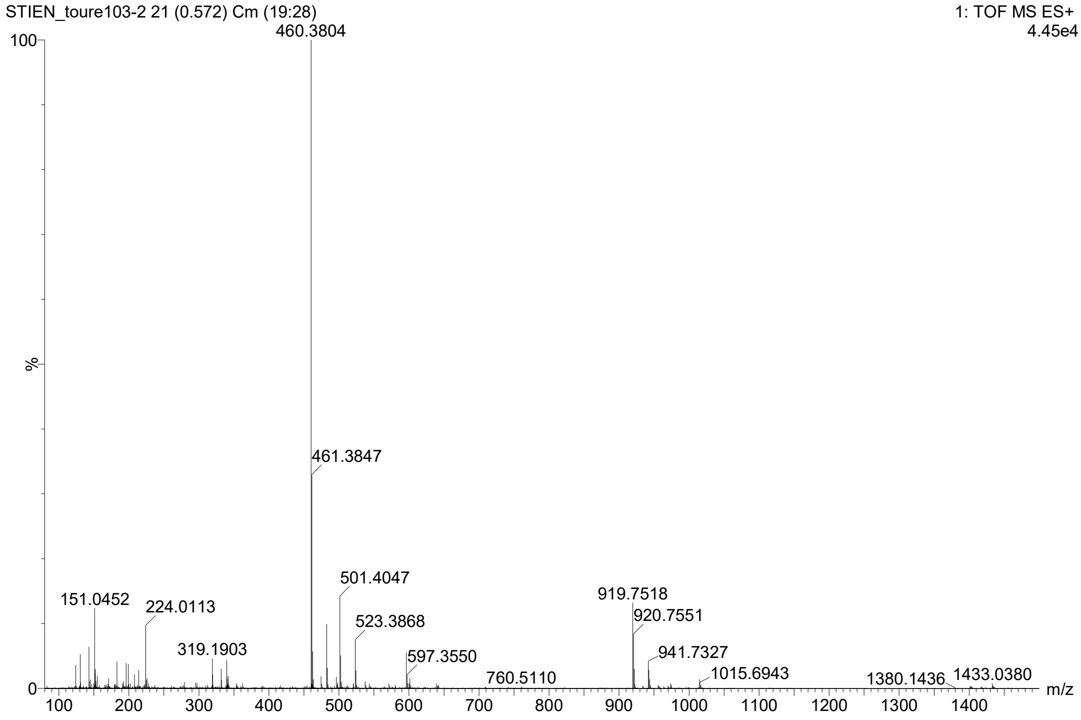

Figure S106. 1H NMR spectrum for compound 29 in CD3OD

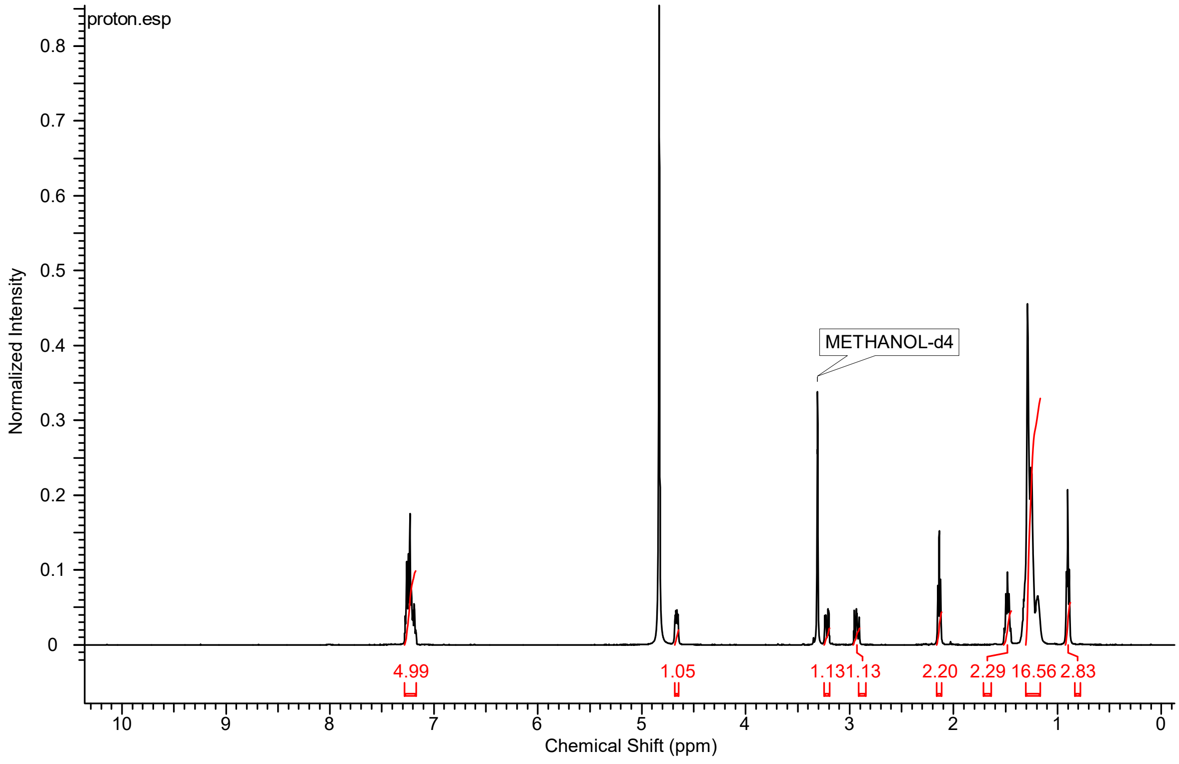

Figure S107. ^{13}C NMR spectrum for compound 29 in CD_3OD

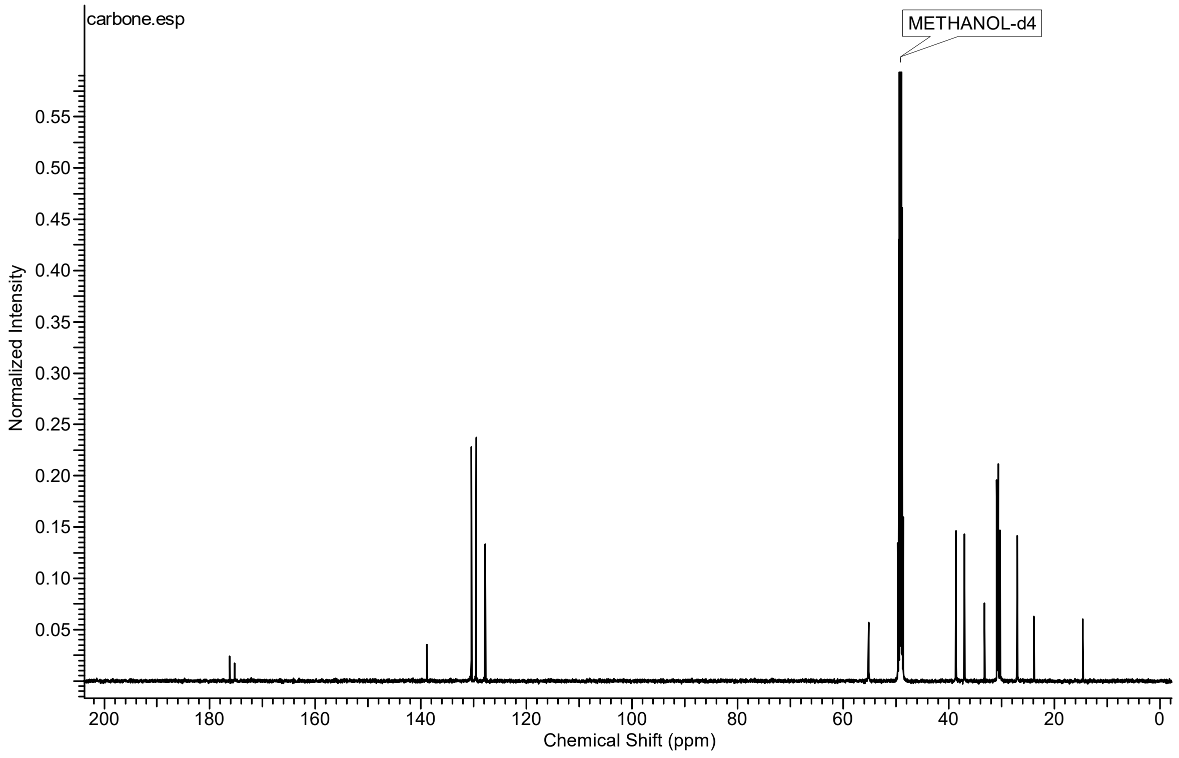

Figure S108. HRMS of compound 29 in MeOH

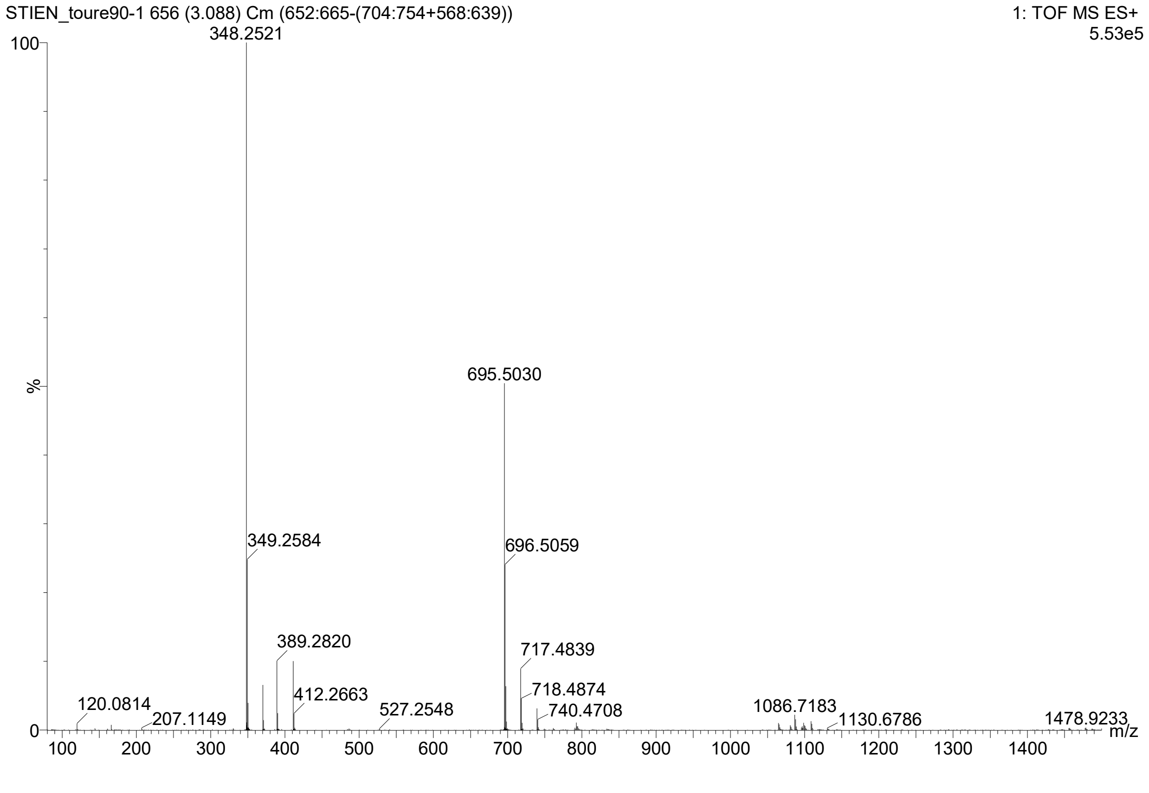

Figure S109. 1H NMR spectrum for compound 30 in CD_3OD

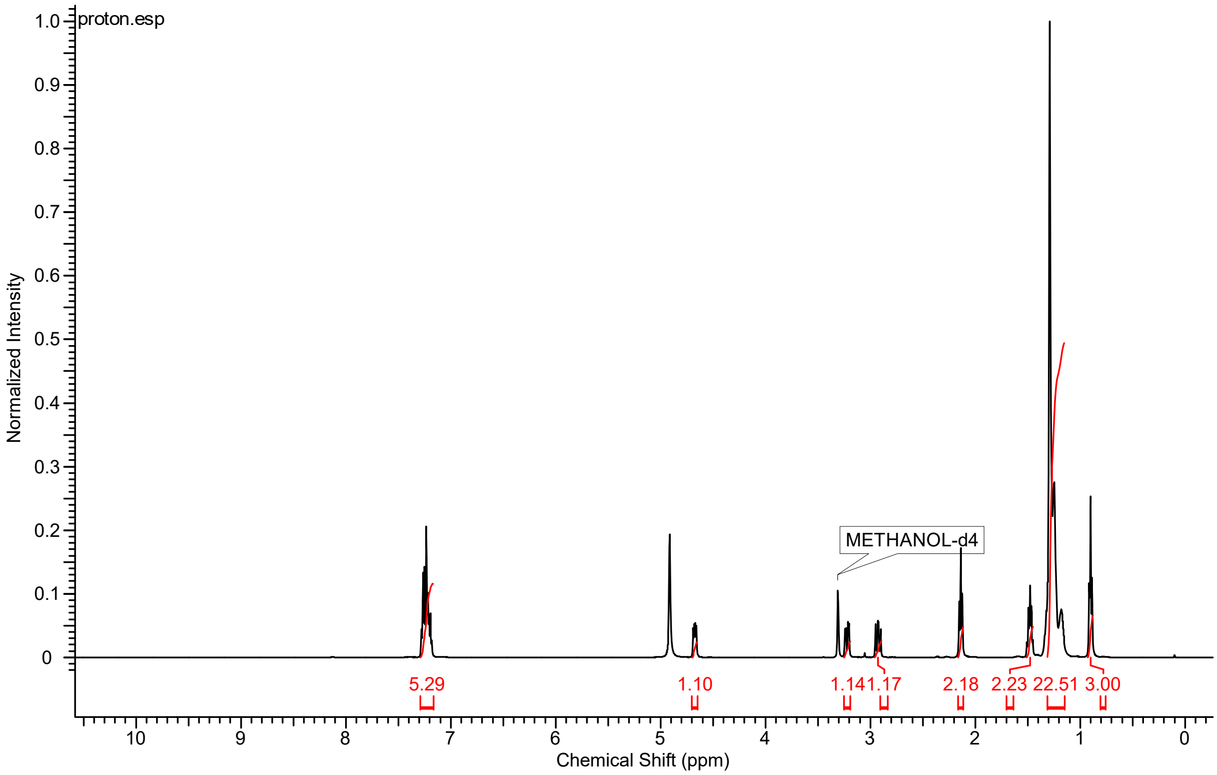

Figure S110. 13C NMR spectrum for compound 30 in CD_3OD

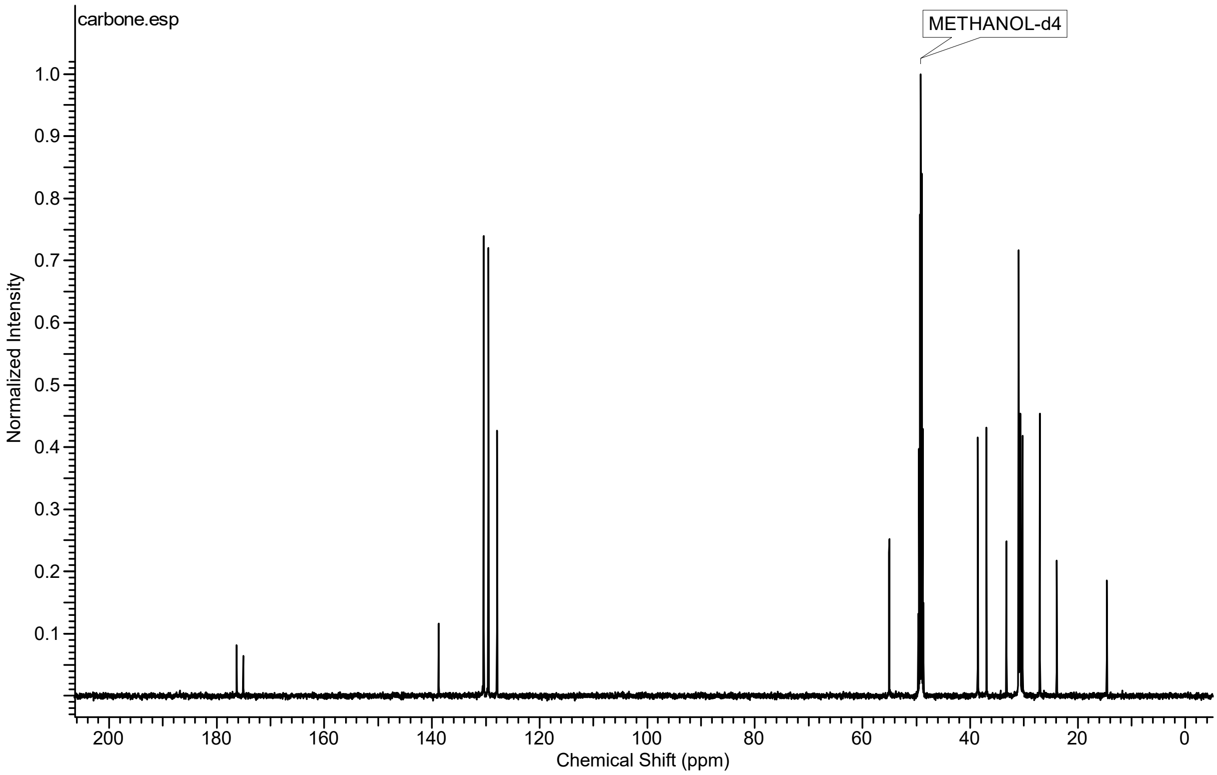

Figure S111. HRMS of compound 30 in MeOH

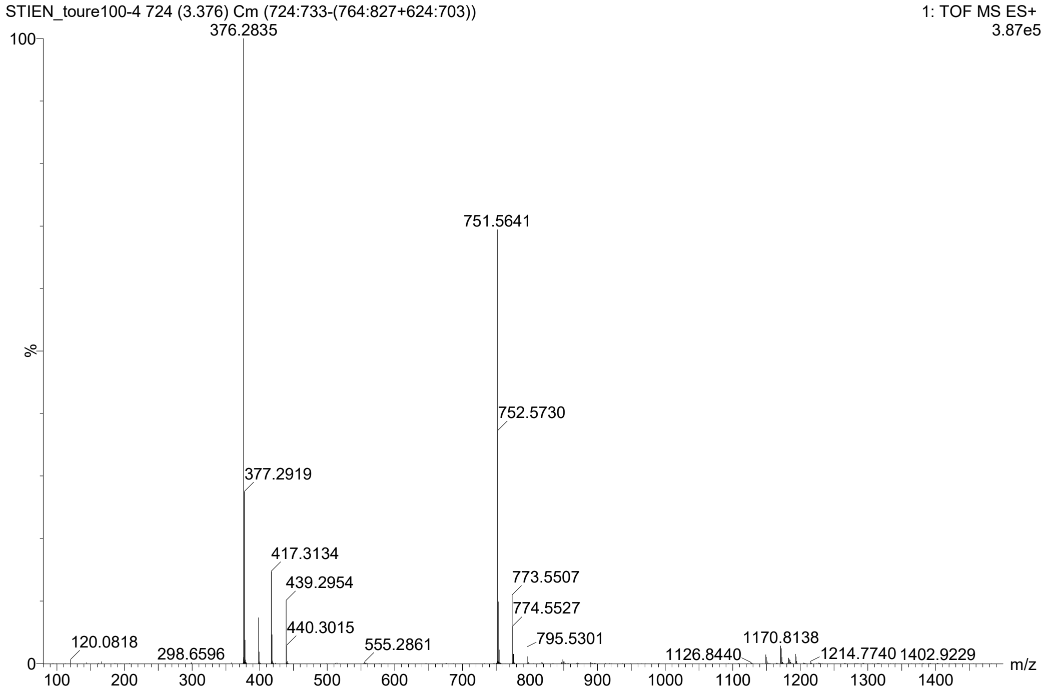

Figure S112. 1H NMR spectrum for compound 31 in CD_3OD

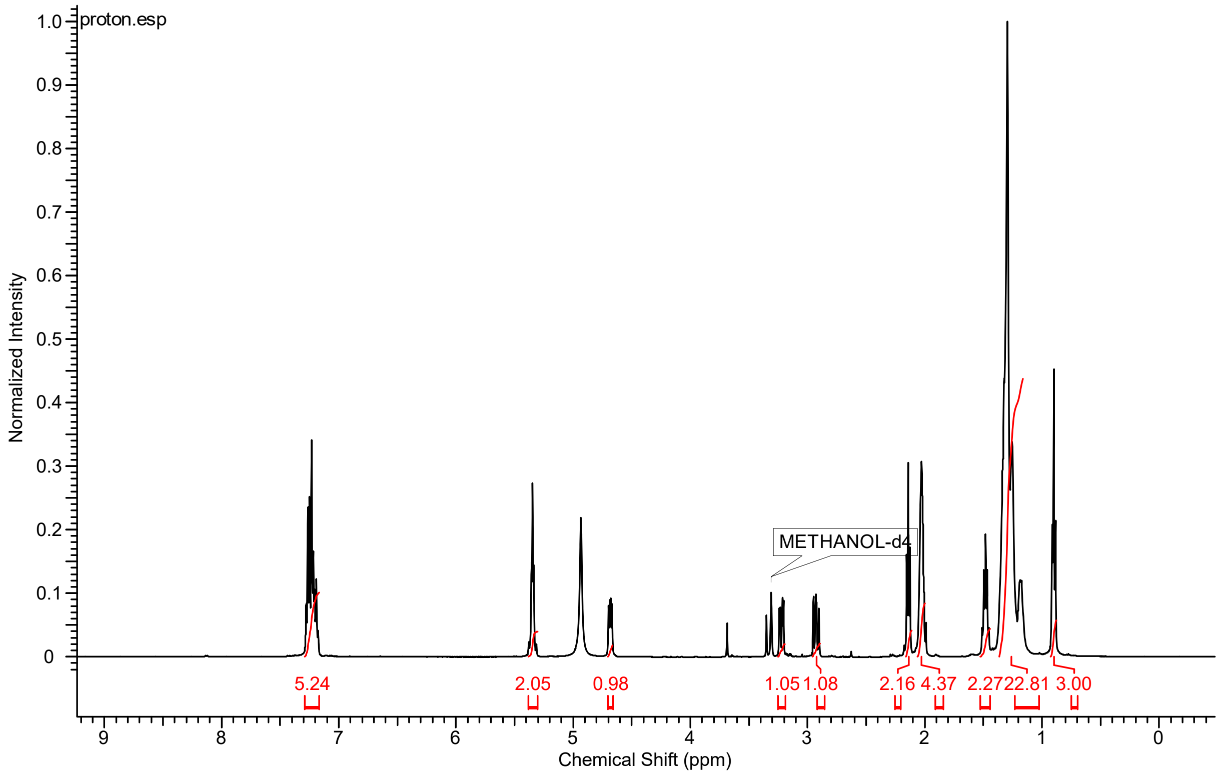

Figure S113. 13C NMR spectrum for compound 31 in CD3OD

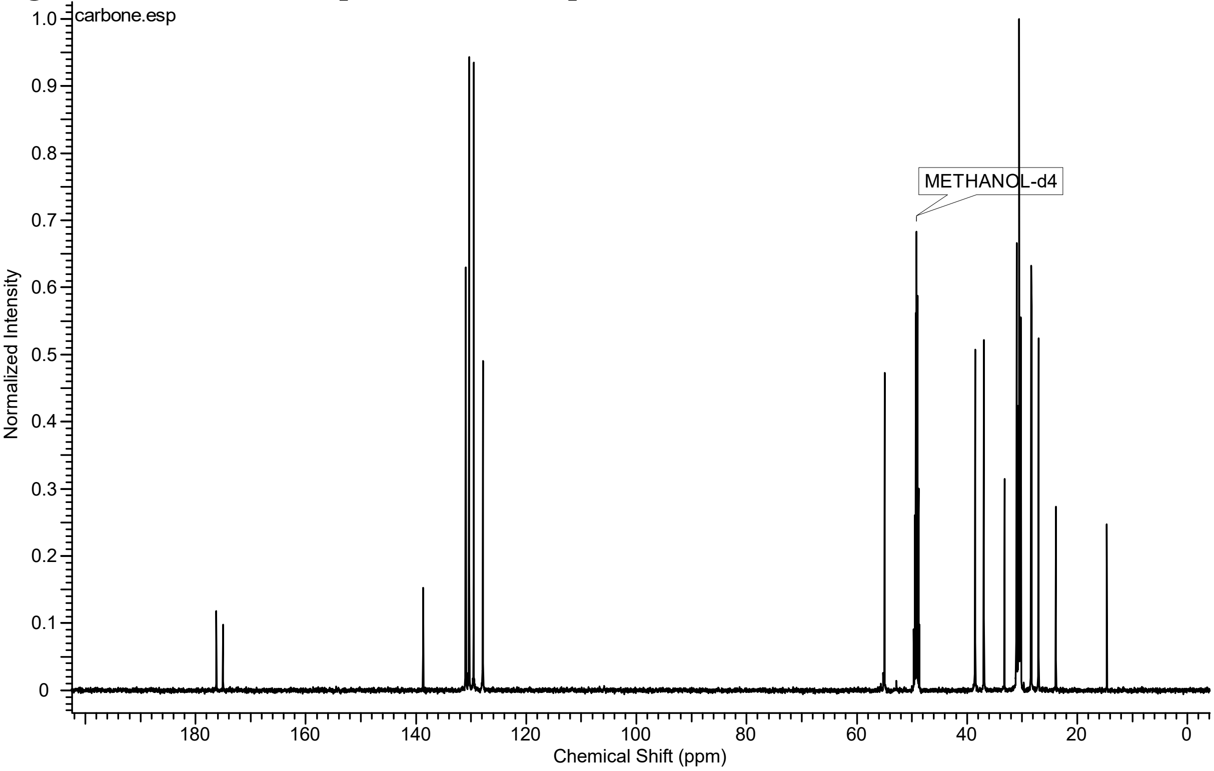

Figure S114. HRMS of compound 31 in MeOH

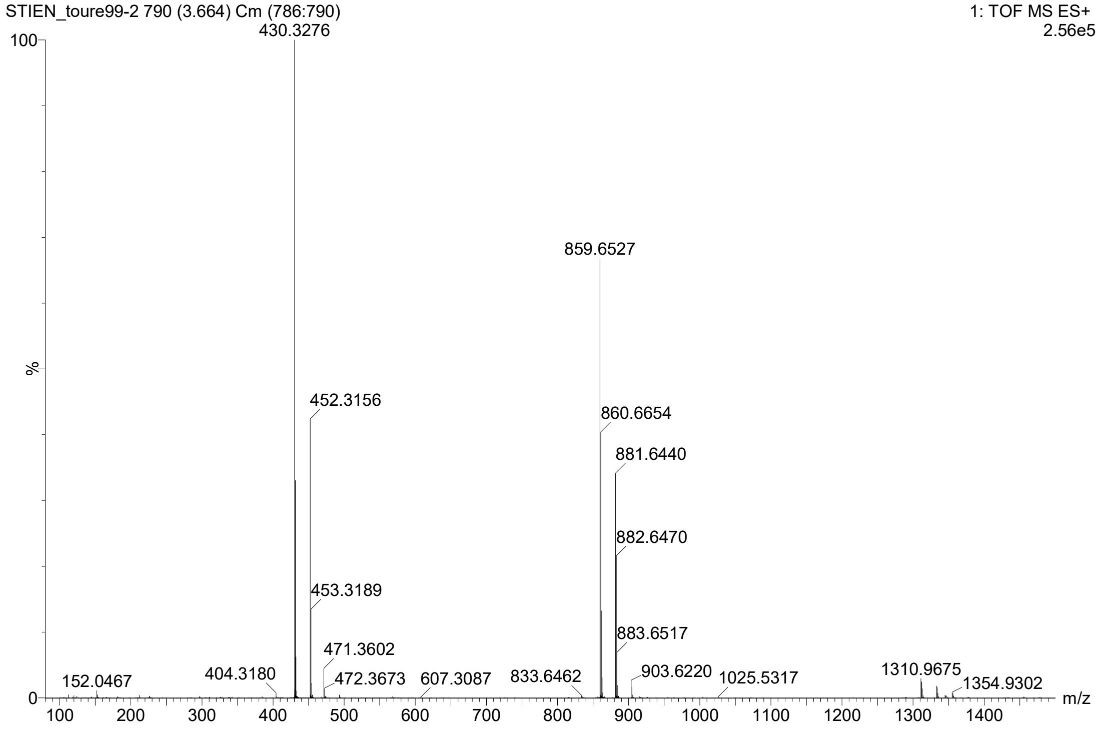

Figure S115. 1H NMR spectrum for compound 32 in CD3OD

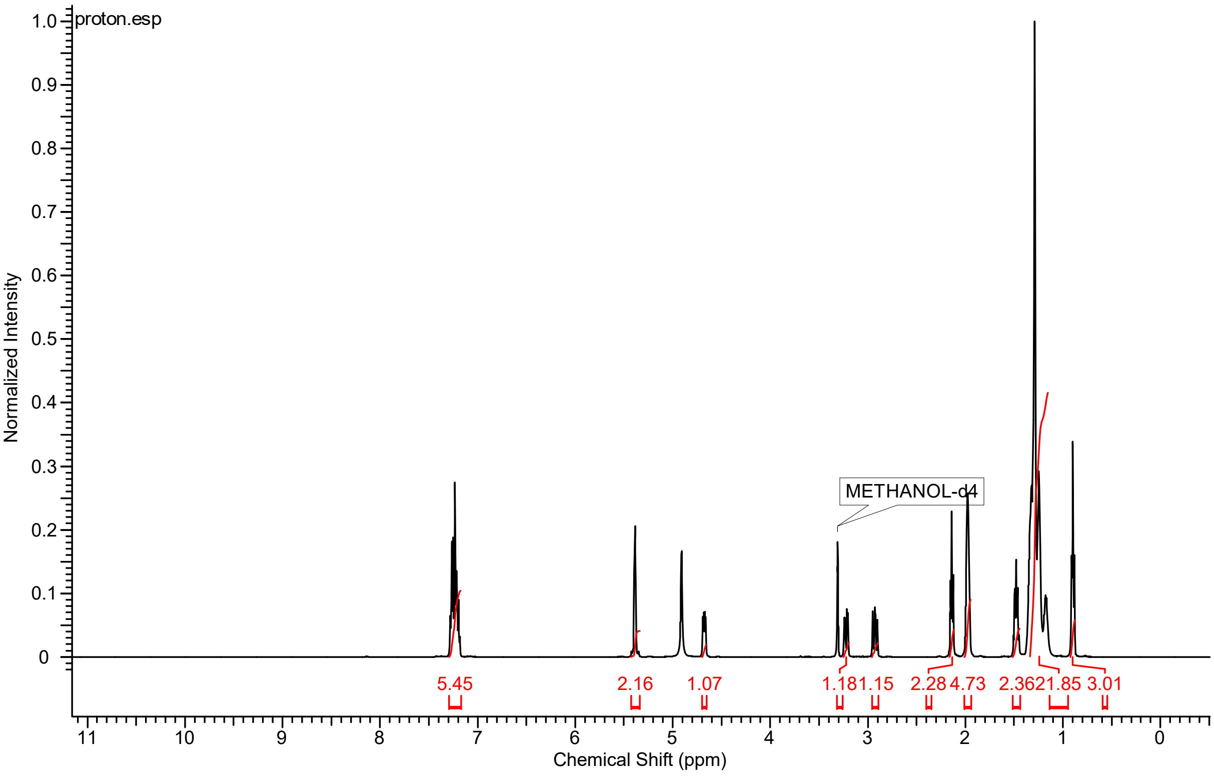

Figure S116. 13C NMR spectrum for compound 32 in CD3OD

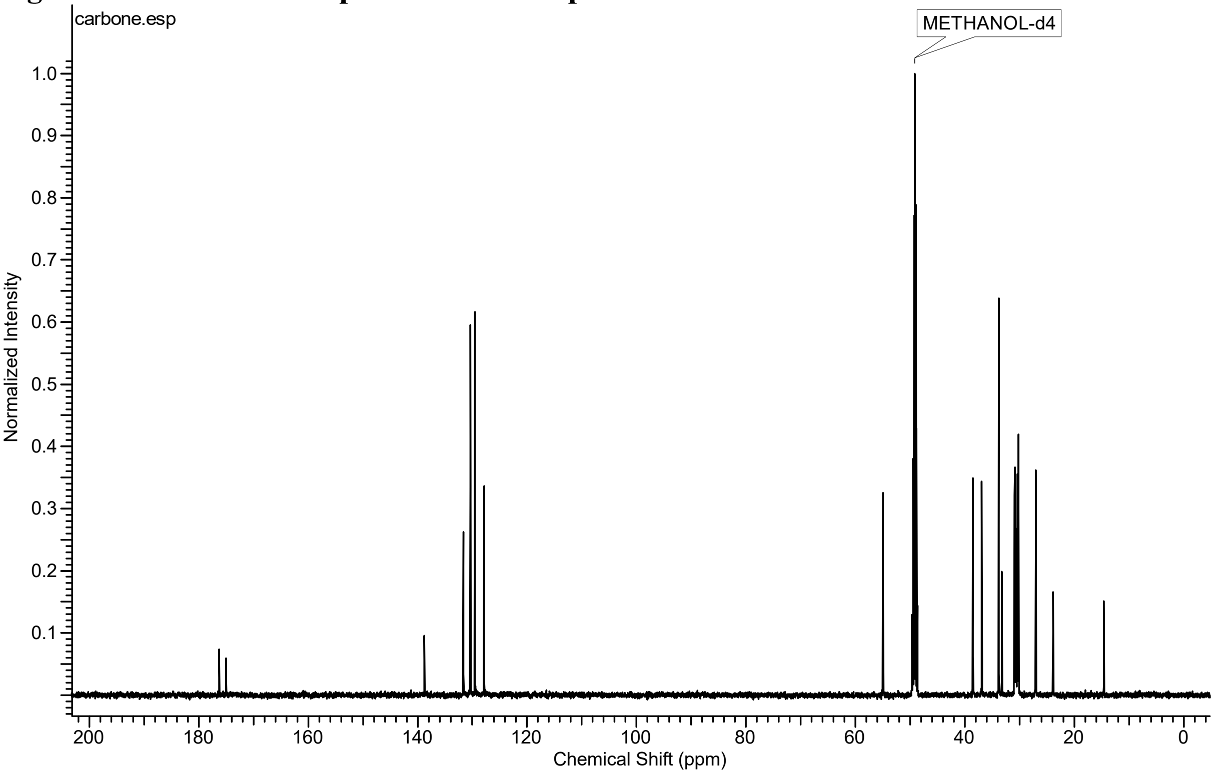

Figure S117. HRMS of compound 32 in MeOH

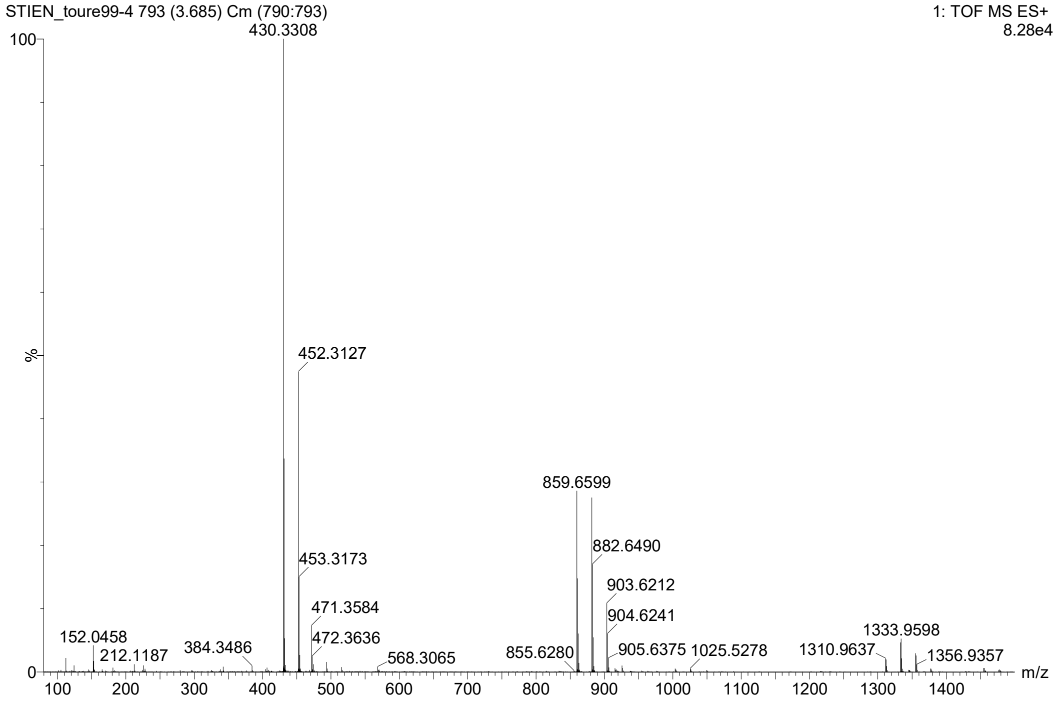

Figure S118. 1H NMR spectrum for compound 33 in CD_3OD

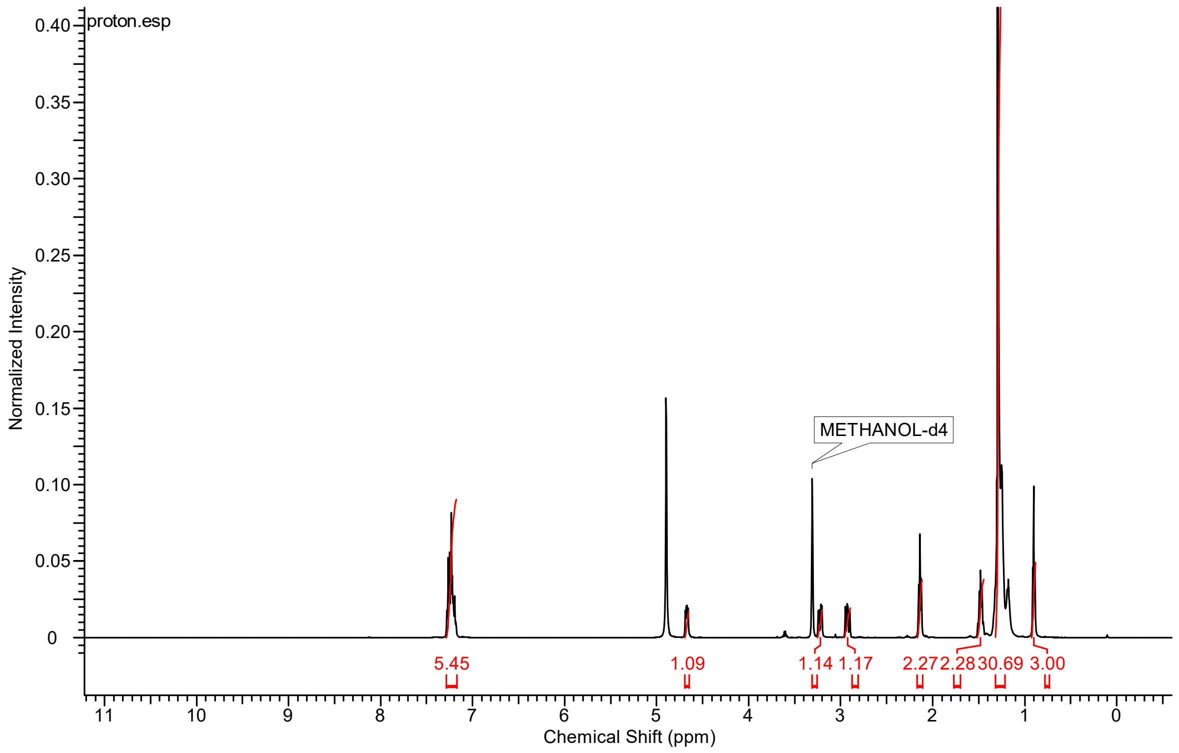

Figure S119. 13C NMR spectrum for compound 33 in CD3OD

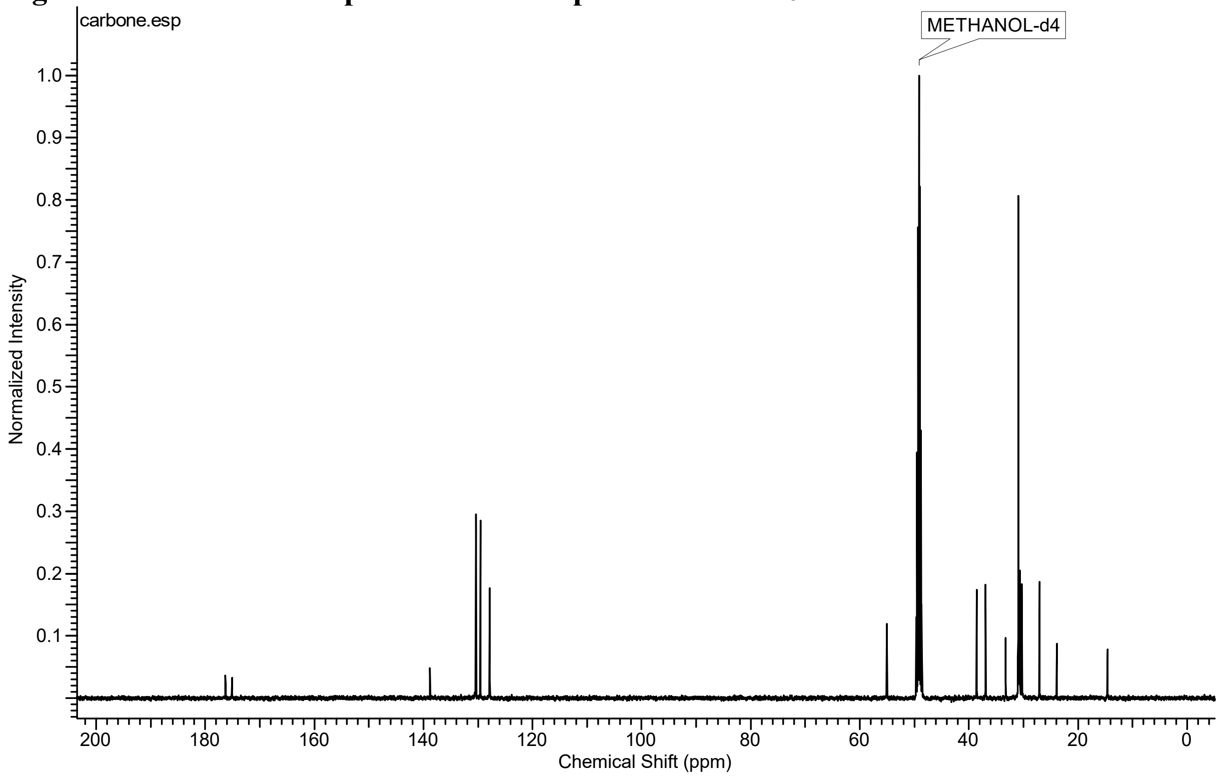

Figure S120. HRMS of compound 33 in MeOH

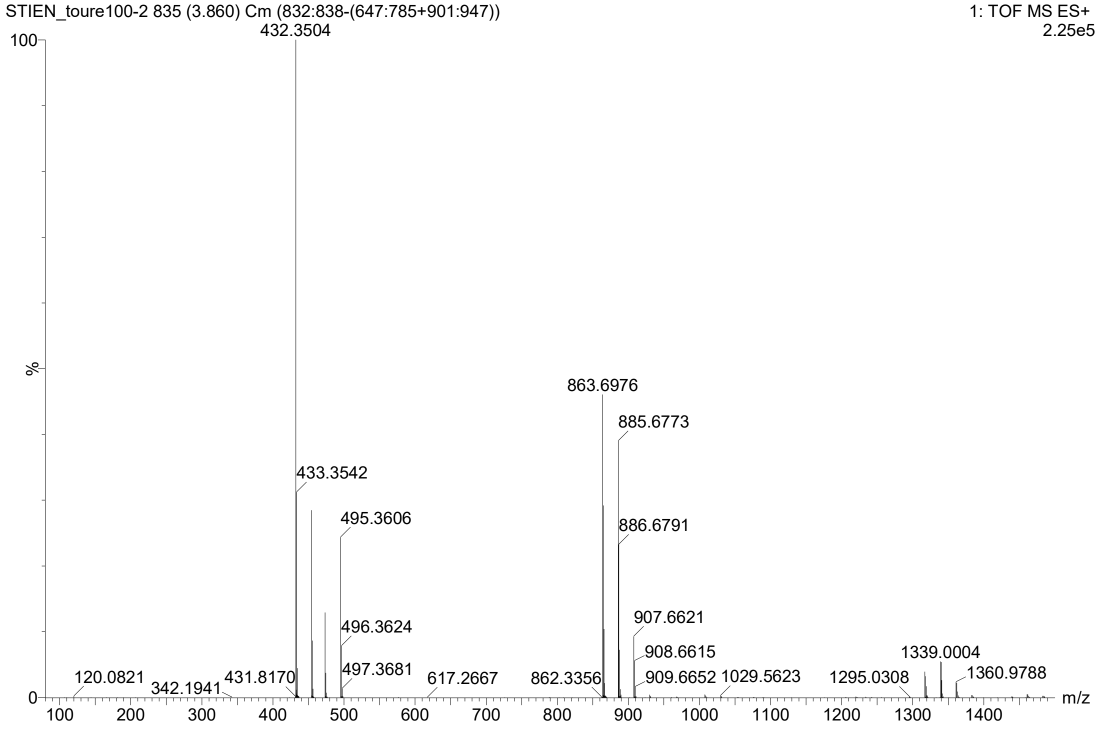

Figure S121. ^{1}H **NMR spectrum for compound 34 in** CD_3OD

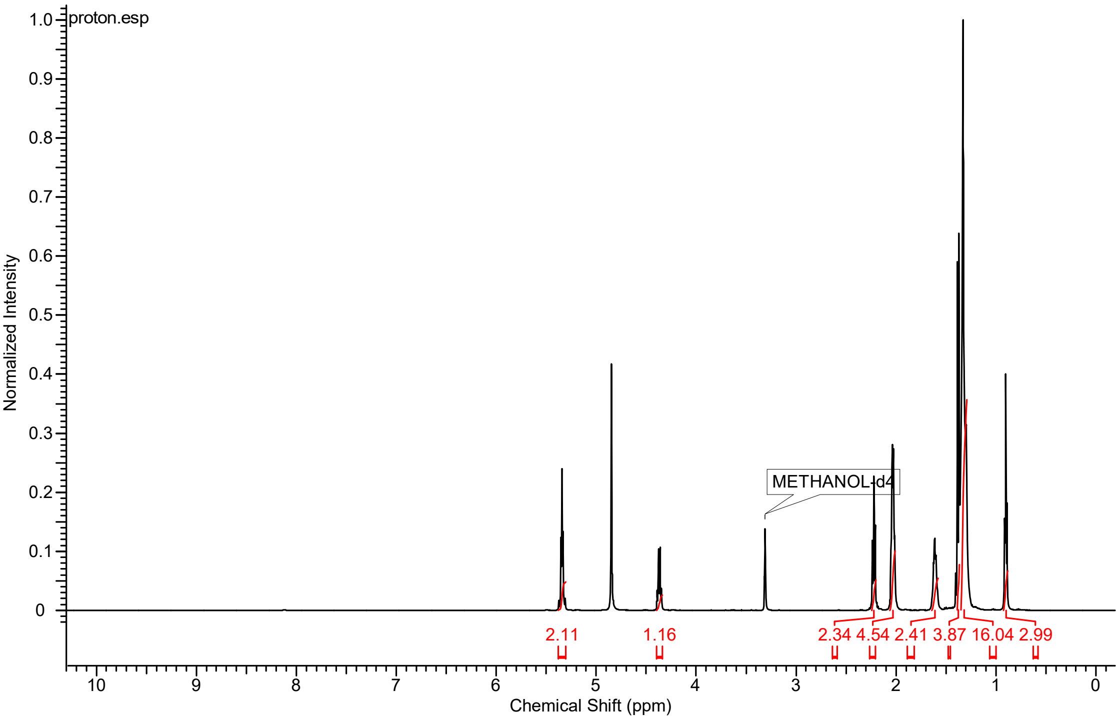

Figure S122. ^{13}C **NMR spectrum for compound 34 in** CD_3OD

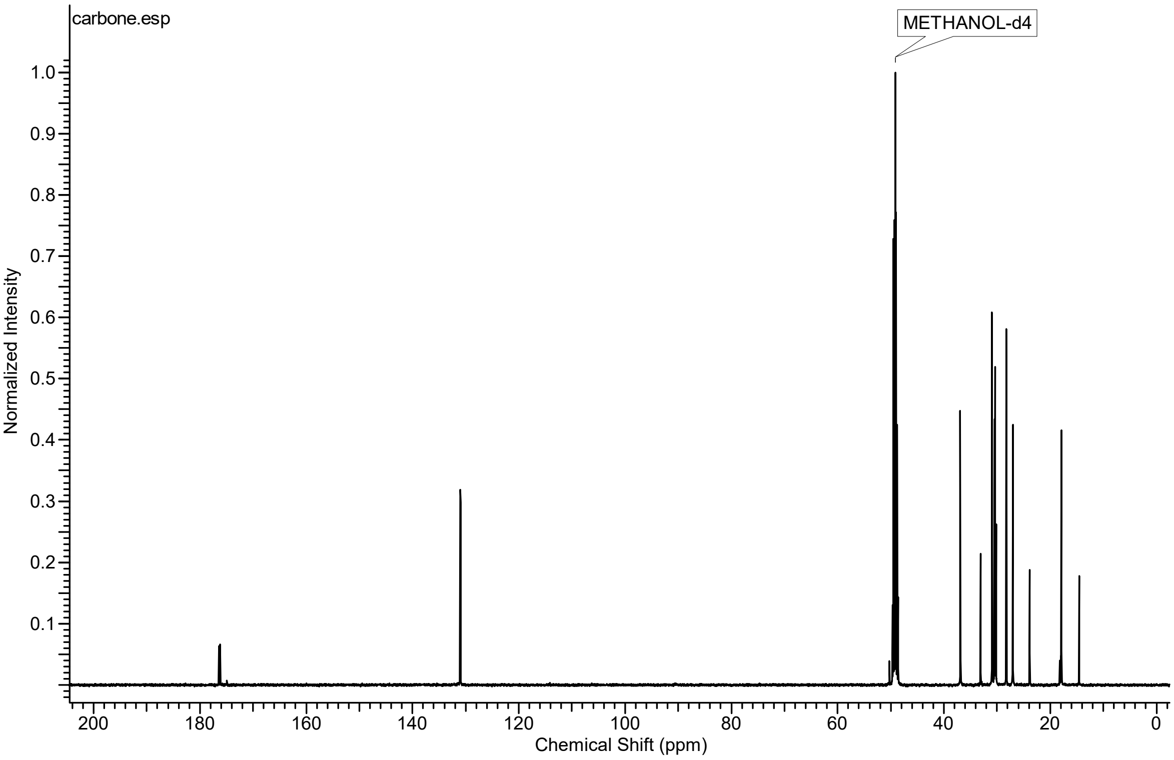

Figure S123. HRMS of compound 34 in MeOH

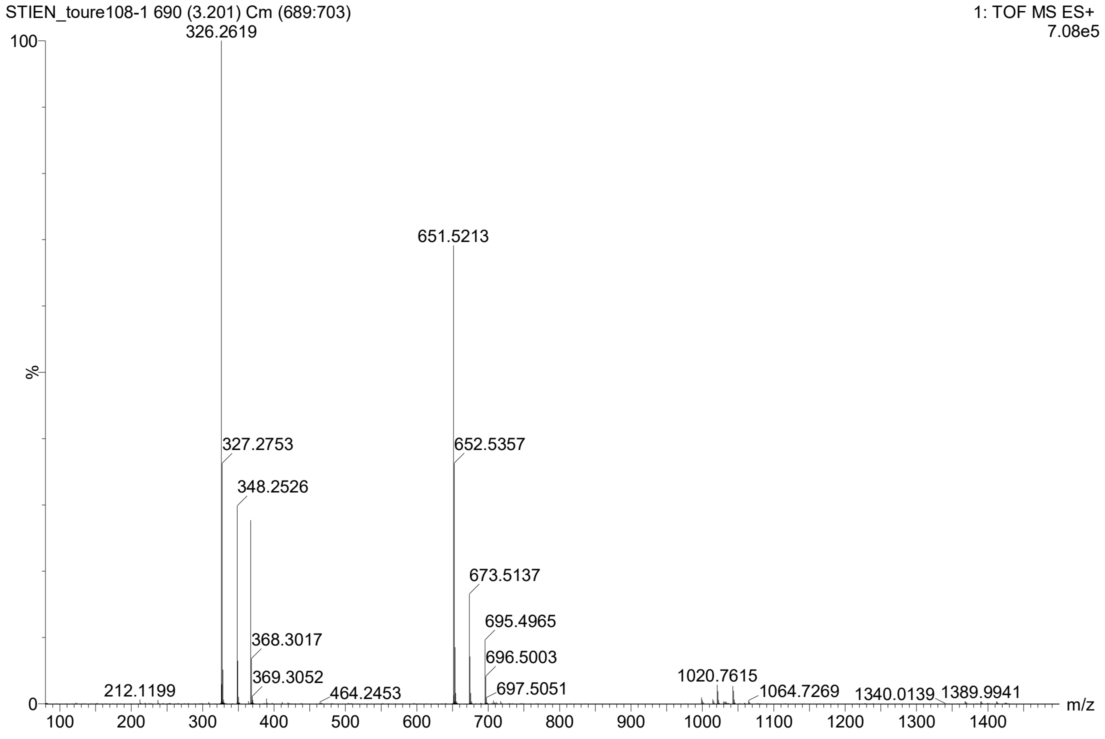

Figure S124. 1H NMR spectrum for compound 35 in CD_3OD

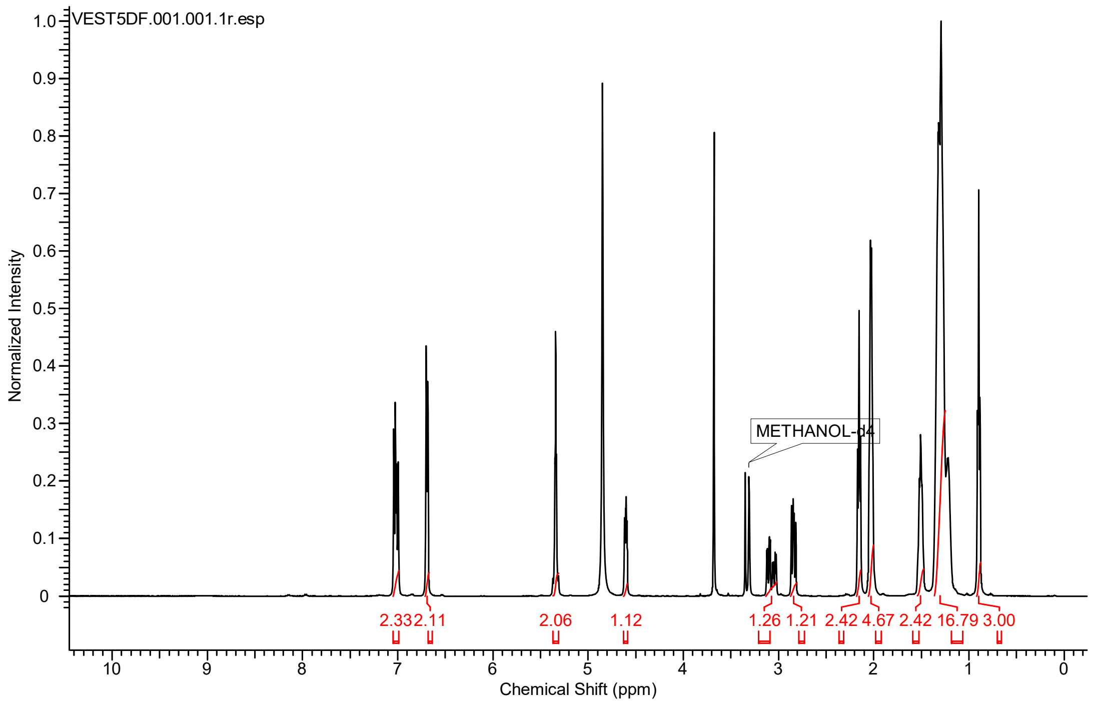

Figure S125. ^{13}C NMR spectrum for compound 35 in CD_3OD

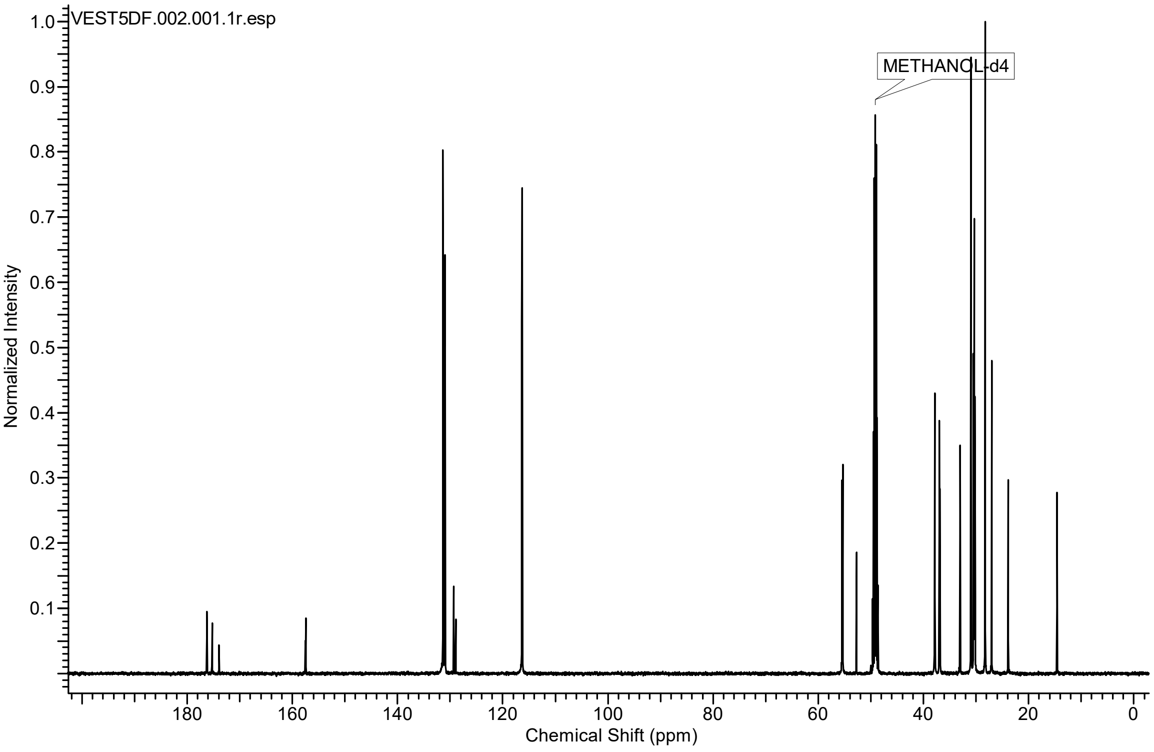

Figure S126. HRMS of compound 35 in MeOH

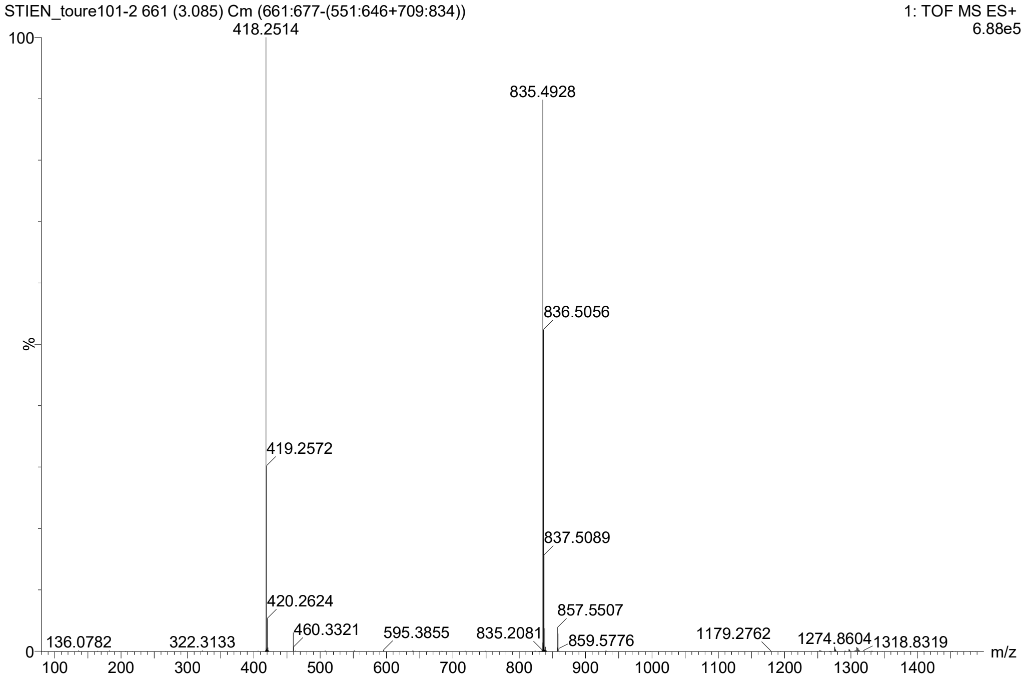

Table S4. Annotation of possible adduct or complexes

Cluster_Pantoea_attributes

SUID	Precursor Intensity in *Pantoea* extract	Precursor mass	Row identity (all IDs)	Row m/z	Row retention time	Molecular formula	Exact mass	RDBE	Mass difference (ppm)	Isotope pattern score	Sirius molecular formula	Phenylalanine typical fragment Exact Mass: 166.09	Phenylalanine methyl ester typical fragment Exact Mass: 180.10	Leucine/Isoleucine typical fragments Exact Mass: 132.10	Leucine/Isoleucine methyl ester typical fragments Exact Mass: 146.12	Valine typical fragments Exact Mass: 118.09
**459**	3.6793E+09	396.348		396.34750	5.7970	C24H45NO3	395.33994	3.00	0.60	98.40	C24H45NO3	-	-	X	-	-
**452**	3.3043E+09	370.332		370.33185	5.7350	C22H43NO3	369.32429	2.00	0.60	99.00	C22H43NO3	-	-	X	-	-
**460**	2.8036E+09	368.321		368.32065	5.3541	C22H41NO3	367.3133	3.00	12.70	-	-	-	-	X	-	-
**522**	1.6071E+09	342.301		342.30096	5.2237	C20H39NO3	341.29299	2.00	2.10	99.10	C20H39NO3	-	-	X	-	-
**464**	8.0000E+08	412.343		412.34286	5.2237	C24H45NO4	411.33486	3.00	1.80	97.10	C24H45NO4	-	-	X	-	-
**446**	6.7868E+08	430.332		430.33170	5.7626	C27H43NO3	429.32429	7.00	0.20	99.30	C27H43NO3	X	-	-	-	-
**521**	6.1632E+08	382.333		382.33270	5.6377	C23H43NO3	381.32429	3.00	2.90	99.40	C23H43NO3	-	-	-	X	-
**543**	5.1876E+08	739.656	Complex of 370.33 and 370.33	739.65619	5.7350			-								

**506**	5.1686E+08	404.316	Compound 22	404.31639	5.6967	C25H41NO3	403.30864	6.00	0.10	96.30	C25H41NO3	X	-	-	-	-
**513**	4.9354E+08	402.305		402.30524	5.3197	C25H39NO3	401.2939	7.00	12.00	-	-	X	-	-	-	-
**541**	4.7154E+08	791.687	Complex of 396.347 and 396.347	791.68744	5.7902	-										
**490**	4.2551E+08	735.634	Complex of 368.321 and 368.321	735.63391	5.3403	-										
**454**	4.2179E+08	340.286		340.28568	4.8731	C20H37NO3	339.27734	3.00	3.10	96.80	C20H37NO3	-	-	X	-	-
**466**	3.4560E+08	368.317		368.31683	5.4021	C22H41NO3	367.30864	3.00	2.30	96.80	C22H41NO3	-	-	X	-	-
**496**	3.2235E+08	446.327		446.32730	5.2579	C27H43NO4	445.31921	7.00	1.80	92.50	C27H43NO4	X	-	-	-	-
**480**	3.0801E+08	384.348		384.34814	6.0759	C23H45NO3	383.33994	2.00	0.90	96.80	C22H41NO4	-	-	-	X	-
**477**	2.9037E+08	358.296		358.29568	4.5795	C20H39NO4	357.28791	2.00	1.70	95.80	C20H39NO4	-	-	X	-	-
**502**	2.7532E+08	376.285		376.28522	5.2237	C23H37NO3	375.27734	6.00	1.50	97.60	C23H37NO3	X	-	-	-	-
**514**	2.4637E+08	410.364		410.36404	6.1193	C25H47NO3	409.35559	3.00	2.70	98.10	C25H47NO3	-	-	-	X	-
**500**	2.4420E+08	354.3		354.30032	5.1758	C21H39NO3	353.29299	3.00	0.00	97.50	C21H39NO3	-	-	-	X	-
**481**	2.3223E+08	356.317		356.31699	5.6377	C21H41NO3	355.30864	2.00	3.00	96.50	C21H41NO3	-	-	X	-	-
**453**	1.9196E+08	314.27		314.26950	4.6819	C18H35NO3	313.26169	2.00	4.60	96.90	C18H35NO3	-	-	X	-	-

501	1.5699E+08	382.333		382.33270	5.7350	C23H43NO3	381.3254	3.00	2.90	96.00	C23H43NO3	-	-	-	X	-
474	1.5298E+08	384.311		384.31125	4.7437	C22H41NO4	383.30356	3.00	0.90	96.00	C22H41NO4	-	-	-	X	-
483	1.4718E+08	825.672	Complex of 396.3475 and 430.3317 m/z	825.67169	5.7694	-										
511	1.3885E+08	765.672	Complex of 394.3319 and 394.3319 m/z	765.67163	5.7558	-										
461	1.1970E+08	356.317		356.31699	5.4824	C21H41NO3	355.3097	2.00	3.00	97.20	C21H41NO3	-	-	X	-	-
488	1.1755E+08	683.594	Complex of 342.3010 and 342.3010 m/z	683.59442	5.2374	-										
523	1.1663E+08	386.326		386.32635	5.1207	C22H43NO4	385.3191	2.00	0.30	96.90	C22H43NO4	-	-	X	-	-
**478**	**1.1572E+08**	**392.28**	**Compound 1**	**392.28015**	**4.6271**	**C23H37NO4**	**391.2729**	**6.00**	**1.60**	**97.80**	**C23H37NO4**	**X**	**-**	**-**	**-**	**-**
484	1.0407E+08	799.656	Complex of 370.3318 and 430.3317 m/z	799.65607	5.7558	-										
527	1.0315E+08	773.641	Complex of 370.3318 and 404.3164 m/z	773.64050	5.7281	-										
489	8.5408E+07	342.305		342.30472	5.2992	C20H39NO3	341.29740	2.00	12.90	-	-	-	-	X	-	-
450	8.4230E+07	328.284		328.28455	5.0452	C19H37NO3	327.2772	2.00	0.40	94.50	C19H37NO3	-	-	-	-	X
467	8.1141E+07	398.364		398.36435	6.1410	C24H47NO3	397.357	2.00	3.50	97.50	C24H47NO3	-	-	X	-	-
473	7.7545E+07	416.318		416.31802	5.6760	C26H41NO3	415.3094	7.00	1.20	97.70	C26H41NO3	-	X	-	-	-

**475**	**6.7081E+07**	**418.295**	**Compound 2**	**418.29517**	**4.7877**	**C25H39NO4**	**417.2875**	**7.00**	**0.00**	**97.60**	**C25H39NO4**	**X**	**-**	**-**	**-**	**-**
**456**	6.5245E+07	374.27		374.26999	4.8731	C23H35NO3	373.26270	7.00	2.70	97.80	C23H35NO3	X	-	-	-	-
**445**	6.2314E+07	444.348	Compound 8 / Compound 9	444.34761	6.0674	C28H45NO3	443.3411	7.00	2.60	92.70	C28H45NO3	-	X	-	-	-
**504**	5.6593E+07	426.358		426.35834	5.6241	C25H47NO4	425.35	3.00	1.20	95.10	C25H47NO4	-	-	-	X	-
**536**	5.2965E+07	426.297	MZ+2Na-2H+ 43.9639 m/z adduct of 382.3327 m/z	426.29663	5.6377											
**493**	5.2741E+07	859.656	Complex of 430.3317 and 430.3317	859.65613	5.7558											
**505**	5.0209E+07	418.332	Compound 4	418.33189	6.0100	C26H43NO3	417.3246	6.00	0.70	98.40	C26H43NO3	-	X	-	-	
**498**	4.9744E+07	420.313		420.31256	5.1549	C25H41NO4	419.30530	6.00	4.10	96.20	C25H41NO4	X	-	-	-	-
**479**	4.6746E+07	446.332	MZ+Na-H+ 21.9819 m/z adduct of 368.3206 m/z	446.33212	5.2992											
**449**	**4.1934E+07**	**402.301**	**Compound 3 / compound *ent*-3**	**402.30092**	**5.3658**	**C25H39NO3**	**401.2938**	**7.00**	**2.00**	**93.40**	**C25H39NO3**	**X**	**-**	**-**	**-**	**-**
**447**	4.1469E+07	368.317		368.31683	5.6377	C22H41NO3	367.3095	3.00	2.30	93.80	C22H41NO3	-	-	X	-	-
**528**	3.9023E+07	717.579	Complex of 342.3009 and 376..2875	717.57904	5.2374											
**495**	3.4215E+07	384.348		384.34814	5.9403	C23H45NO3	383.3408	2.00	2.20	95.10	C23H45NO3	-	-	-	X	-
**526**	3.3885E+07	354.3		354.30032	5.2923	C21H39NO3	353.2930	3.00	0.00	95.60	C21H39NO3	-	-	-	X	-

519	3.2348E+07	348.253	Compound 22	348.25327	4.7068	C21H33NO3	347.2459	6.00	0.40	95.30	C21H33NO3	X	-	-	-	-
469	3.0608E+07	763.658	Complex of 382.33 and 382.33	763.65814	5.6377											
491	3.0448E+07	390.302	Compound 14	390.30176	5.6024	C24H39NO3	389.2951	6.00	5.40	?	C24H39NO3	-	X	-	-	-
503	2.8560E+07	426.297	MZ+Na-H+ 21.9819 m/z adduct of 404.3167 m/z	426.29755	5.6933											
533	2.7014E+07	412.347		412.34729	5.2992	C24H45NO4	411.3	3.00	12.50	-	-	-	-	X	-	-
512	2.6148E+07	354.305		354.30478	5.2992	C21H39NO3	353.2975	3.00	12.50	-	-	-	-	-	X	-
531	2.3163E+07	807.625	2M+H of 404.3167 m/z	807.62521	5.6886											
494	2.1671E+07	803.602	Complex of 402.3052 and 402.3052 m/z	803.60248	5.3266											
509	2.0780E+07	390.302		390.30237	5.4824	C24H39NO3	389.2951	6.00	5.40	93.6	C24H39NO3	X	-	-	-	-
462	1.9570E+07	424.379		424.37918	6.1706	C26H49NO3	423.37190	3.00	1.50	95.80	C26H49NO3	-	-	X	-	-
532	1.8960E+07	455.422		455.42206	6.1262	-										
472	1.8484E+07	460.343		460.34280	5.6172	C28H45NO4	459.33550	7.00	1.40	90.40	C28H45NO4	-	X	-	-	-
463	1.6858E+07	344.279		344.27927	4.3946	C19H37NO4	343.272	2.00	0.80	95.50	C19H37NO4	-	-	-	-	X
540	1.6769E+07	751.656	Complex of 396.3475 and 356.317 m/z	751.65564	5.7350											

**524**	1.5413E+07	414.358	MZ+CH3OH 32.0262 m/z adduct of 382.3327 m/z	414.35843	5.6241	C24H47NO4	413.3511	2.00	1.40	92.00	C24H47NO4	-	-	X	-	-
**451**	1.4940E+07	376.289	MZ+Na-H+ 21.9819 m/z adduct of 354.3048 m/z	376.28894	5.2992	-										
**457**	1.4074E+07	286.238		286.23843	4.1356	C16H31NO3	285.2311	2.00	2.50	95.70	C16H31NO3	-	-	X	-	-
**517**	1.3691E+07	767.689	Complex of 384.3481 and 384.3481 m/z	767.68866	6.0691											
**486**	1.2397E+07	412.285	MZ+2Na-2H+ 43.9639 m/z adduct of 368.3206 m/z	412.28497	5.3884											
**535**	1.1930E+07	429.406		429.40601	6.0691	-										
**485**	1.0689E+07	412.28	MZ+2Na-2H+ 43.9639 m/z adduct of 368.3206 m/z	412.28055	5.4021											
**516**	8.4877E+06	370.296		370.29611	4.5659	C21H39NO4	369.28880	3.00	2.40	94.20	C21H39NO4	-	-	X	-	-
**476**	8.0609E+06	356.28		356.27963	4.2648	C20H37NO4	355.2723	3.00	0.10	95.60	C20H37NO4	-	-	-	-	X
**510**	7.9807E+06	369.298	MZ+Na-H+ 21.9819 m/z adduct of 347.3153 m/z	369.29771	5.5360											
**499**	7.1568E+06	418.332		418.33240	5.9256	C26H43NO3	417.3246	6.00	0.70	98.40	C26H43NO3	-	X	-	-	-
**455**	6.9899E+06	312.253		312.25333	4.4015	C18H33NO3	311.246	3.00	0.10	95.00	C18H33NO3	-	-	X	-	-
**470**	6.5649E+06	300.253		300.25281	4.4909	C17H33NO3	299.246	2.00	0.10	95.10	C17H33NO3	-	-	-	-	-
**537**	6.2433E+06	382.294		382.29422	4.3014	C22H39NO4	381.28690	4.00	2.60	94.10	C22H39NO4	-	-	X	-	-

**448**	6.0605E+06	366.301		366.30118	4.1892	C22H39NO3	365.2939	4.00	2.50	94.00	C22H39NO3	-	-	-	-	-
**518**	5.2887E+06	819.721	Complex of 410.3640 and 410.3640 m/z	819.72046	6.1193											
**465**	5.0036E+06	330.264		330.26404	4.0308	C18H35NO4	329.2567	2.00	0.30	96.10	C18H35NO4	-	-	X	-	-
**458**	4.2644E+06	320.222		320.22226	4.1650	C19H29NO3	319.21540	6.00	2.10	95.60	C19H29NO3	X	-	-	-	-
**482**	4.1600E+06	398.364		398.36435	6.2616	C24H47NO3	397.357	2.00	3.50		C24H47NO3	-	-	X	-	-
**468**	4.1596E+06	412.379		412.37878	6.4385	C25H49NO3	411.3719	2.00	1.60		C25H49NO3	-	-	-	X	
**539**	3.8162E+06	428.312	MZ+2Na-2H+ 43.9639 m/z adduct of 384.3481 m/z	428.31189	5.9403											
**515**	3.5006E+06	463.39	2M+Na of 418.3314 m/z	463.38972	6.0216											
**530**	3.1346E+06	489.405	M+2Na adduct of 444.3472 m/z	489.40532	6.0579											
**542**	3.0963E+06	458.364		458.36368	6.1410	C29H47NO3	457.3564	7.00	1.80	90.50	C29H47NO3	x	-	-	-	-
**492**	1.7258E+06	435.359	M+2Na of 390.3011 m/z	435.35910	5.5954											
**497**	0.0000E+00	831.631	2M+H of 416.31 m/z	831.63123	5.6655											
**507**	0.0000E+00	947.779	2M+H of 474.3936 m/z	947.77942	6.5812											
**508**	0.0000E+00	779.595	2M+H of 390.3011 m/z	779.59497	5.5877											

**520**	0.0000E+00	695.499	2M+H of 348.2533 m/z	695.49933	4.7258											
**525**	0.0000E+00	803.593	2M+H of 402.3006 m/z	803.59332	5.3191											
**529**	0.0000E+00	887.687	2M+H adduct of 444.3473 m/z	887.68680	6.0596											
**534**	0.0000E+00	835.655	2M+H of 418.3314 m/z	835.65515	5.9878											
**538**	0.0000E+00	891.719	2M+H adduct of 446.3633 m/z	891.71893	6.3044											
**471**	0.0000E+00	474.394	Compound 5	474.39355	6.5731	C30H51NO3	473.3863	6.00	1.30	98.50	C30H51NO3	-	X	-	-	-
**487**	0.0000E+00	446.363	Compound 10	446.36331	6.3044	C28H47NO3	445.35600	6.00	0.90	99.00	C28H47NO3	-	X	-S	-	-

Table S5. Optical rotation of all synthetic compounds

**Compound**	Optical rotation $[\alpha]_D^{20}$ (c 0.1, MeOH)
**(L)-methyl (Z)-hexadec-9-enoylphenylalaninate (3-OMe)**	+54
**(L)-methyl palmitoylphenylalaninate (4)**	+48
**(L)-methyl icosanoylphenylalaninate (5)**	+56
**(L)-methyl dodecanoylphenylalaninate (6)**	+78
**(L)-methyl tetradecanoylphenylalaninate (7)**	+97
**(L)-methyl oleoylphenylalaninate (8)**	+60
**(L)-methyl (E)-octadec-9-enoylphenylalaninate (9)**	+58
**(L)-methyl stearoylphenylalaninate (10)**	+63
**(D)-methyl palmitoylphenylalaninate (11)**	–67
**(D)-methyl (*Z*)-hexadec-9-enoylphenylalaninate (*ent*-3-OMe)**	–49
**(D)-methyl icosanoylphenylalaninate (12)**	–50
**(D)-methyl dodecanoylphenylalaninate (13)**	–49
**(D)-methyl tetradecanoylphenylalaninate (14)**	–87
**(D)-methyl oleoylphenylalaninate (15)**	–55
**(D)-methyl-(*E*)-octadec-9-enoylphenylalaninate (16)**	–83
**(D)-methyl stearoylphenylalaninate (17)**	–46
**(L)-methyl (Z)-hexadec-9-enoylalaninate (18)**	–1
**(L)-methyl (*Z*)-hexadec-9-enoyltyrosinate (19)**	+69
**(L)-palmitoylphenylalanine (20)**	+66
**(L)-(*Z*)-hexadec-9-enoylphenylalanine (3)**	+49
**(L)-icosanoylphenylalanine (21)**	+40
**(L)-dodecanoylphenylalanine (22)**	+67
**(L)-tetradecanoylphenylalanine (23)**	+80
**(L)-oleoylphenylalanine (24)**	+56
**(L)-(*E*)-octadec-9-enoylphenylalanine (25)**	+54
**(L)-stearoylphenylalanine (26)**	+49
**(D)-palmitoylphenylalanine (27)**	–54
**(D)-(Z)-hexadec-9-enoylphenylalanine (*ent*-3)**	–49
**(D)-icosanoylphenylalanine (28)**	–22
**(D)-dodecanoylphenylalanine (29)**	–68
**(D)-tetradecanoylphenylalanine (30)**	–64
**(D)-oleoylphenylalanine (31)**	–41
**(D)-(*E*)-octadec-9-enoylphenylalanine (32)**	–70
**(D)-stearoylphenylalanine (33)**	–50
**(L)-(*Z*)-hexadec-9-enoylalanine (34)**	+1
**(L)-(Z)-hexadec-9-enoyltyrosine (35)**	+53
